# YAP mediates compensatory cardiac hypertrophy through aerobic glycolysis in response to pressure overload

**DOI:** 10.1172/JCI150595

**Published:** 2022-03-15

**Authors:** Toshihide Kashihara, Risa Mukai, Shin-ichi Oka, Peiyong Zhai, Yasuki Nakada, Zhi Yang, Wataru Mizushima, Tsutomu Nakahara, Junco S. Warren, Maha Abdellatif, Junichi Sadoshima

**Affiliations:** 1 Department of Cell Biology and Molecular Medicine, Rutgers New Jersey Medical School, Newark, New Jersey, USA.; 2 Department of Molecular Pharmacology, Kitasato University School of Pharmaceutical Sciences, Tokyo, Japan.; 3 Fralin Biomedical Research Institute, Virginia Tech Carilion, Roanoke, Virginia, USA.

**Keywords:** Cardiology, Cardiovascular disease

## Abstract

The heart utilizes multiple adaptive mechanisms to maintain pump function. Compensatory cardiac hypertrophy reduces wall stress and oxygen consumption, thereby protecting the heart against acute blood pressure elevation. The nuclear effector of the Hippo pathway, Yes-associated protein 1 (YAP), is activated and mediates compensatory cardiac hypertrophy in response to acute pressure overload (PO). In this study, YAP promoted glycolysis by upregulating glucose transporter 1 (GLUT1), which in turn caused accumulation of intermediates and metabolites of the glycolytic, auxiliary, and anaplerotic pathways during acute PO. Cardiac hypertrophy was inhibited and heart failure was exacerbated in mice with YAP haploinsufficiency in the presence of acute PO. However, normalization of GLUT1 rescued the detrimental phenotype. PO induced the accumulation of glycolytic metabolites, including l-serine, l-aspartate, and malate, in a YAP-dependent manner, thereby promoting cardiac hypertrophy. YAP upregulated the GLUT1 gene through interaction with TEA domain family member 1 (TEAD1) and HIF-1**α** in cardiomyocytes. Thus, YAP induces compensatory cardiac hypertrophy through activation of the Warburg effect.

## Graphical abstract

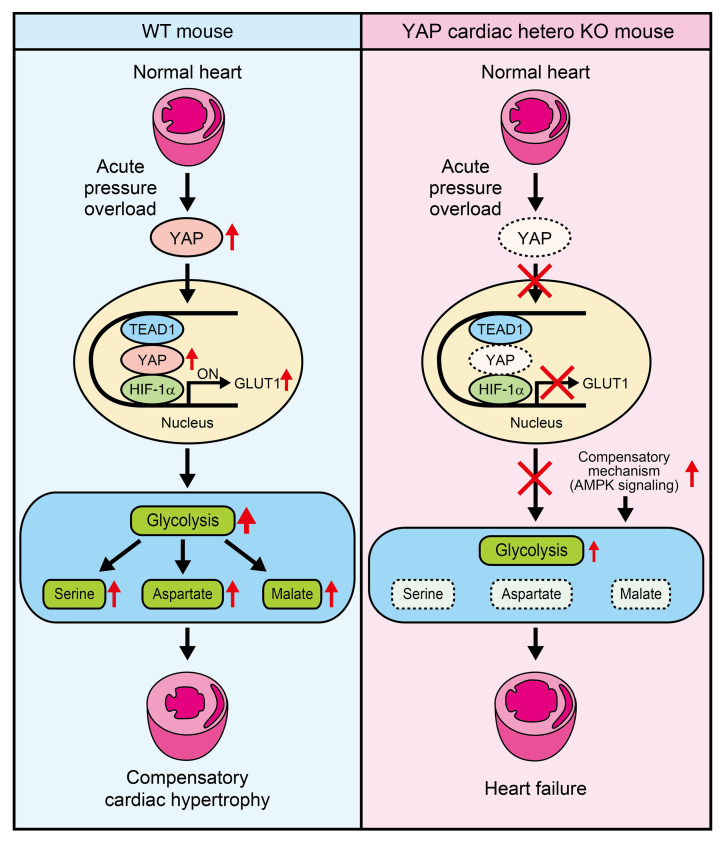

## Introduction

Cardiac hypertrophy is an initial response of the heart to hemodynamic overload, including high blood pressure, termed pressure overload (PO). Long-term cardiac PO also often elicits other pathological effects in cardiac muscle, including cell death, inflammation, and fibrosis, thereby causing contractile dysfunction (1). However, cardiac hypertrophy alone is not always pathological; increasing wall thickness produces more contractile force and reduces wall stress and oxygen consumption. Therefore, hypertrophy can remain adaptive without progressing to failure (1). Indeed, activation of signaling mechanisms that stimulate adaptive hypertrophy can alleviate pathological hypertrophy (1, 2). Conversely, failure to activate adaptive mechanisms during hemodynamic overload is devastating (3, 4). It is thus clinically important to elucidate the signaling mechanism mediating the adaptive form of cardiac hypertrophy.

The Hippo pathway is an evolutionarily conserved signaling pathway that controls organ size and tumorigenesis by regulating cell growth and death (5). Each pathway component is intimately involved in the pathogenesis of heart failure (6–9). Yes-associated protein 1 (YAP), the nuclear effector of the Hippo pathway, is transiently activated in response to PO but then downregulated during chronic PO and heart failure (3, 7). Cardiac-specific downregulation of YAP inhibits cardiac hypertrophy but promotes heart failure during acute PO, indicating that endogenous YAP is salutary and mediates compensatory hypertrophy during acute PO (3). However, the underlying mechanism of this salutary effect remains unknown.

Cancer cells activate glycolysis even under aerobic conditions to optimize the flux of glucose and amino acids into biosynthetic pathways. This phenomenon, termed the Warburg effect, may promote cell proliferation and survival (10). A close similarity between YAP-mediated glycolysis and the Warburg effect has been observed in some cancer cells (11). The glycolytic mechanism also plays an important role in mediating cardiac hypertrophy (12). However, the detailed molecular mechanism through which cardiomyocytes (CMs) upregulate glycolysis, leading to hypertrophy in response to PO, is not well understood (13). We hypothesized that YAP mediates compensatory cardiac hypertrophy during acute PO through activation of the Warburg effect. Here, we investigated whether upregulation of glycolysis in response to acute PO is preserved when upregulation of endogenous YAP is inhibited and sought to determine which downstream mechanisms mediate YAP-induced compensatory hypertrophy during PO in the heart.

## Results

### Endogenous YAP plays an essential role in mediating glucose transporter 1 upregulation and glycolysis in response to PO.

We have shown previously (3) that YAP is activated in response to acute PO, and that cardiac-specific heterozygous downregulation of YAP (YAPch-KO) normalizes nuclear YAP levels, inhibits hypertrophy despite the presence of cardiac dilation, and promotes heart failure during acute PO. This suggests that endogenous YAP promotes compensatory (adaptive) cardiac hypertrophy and protects the heart against heart failure during acute PO.

**Figure 1:**
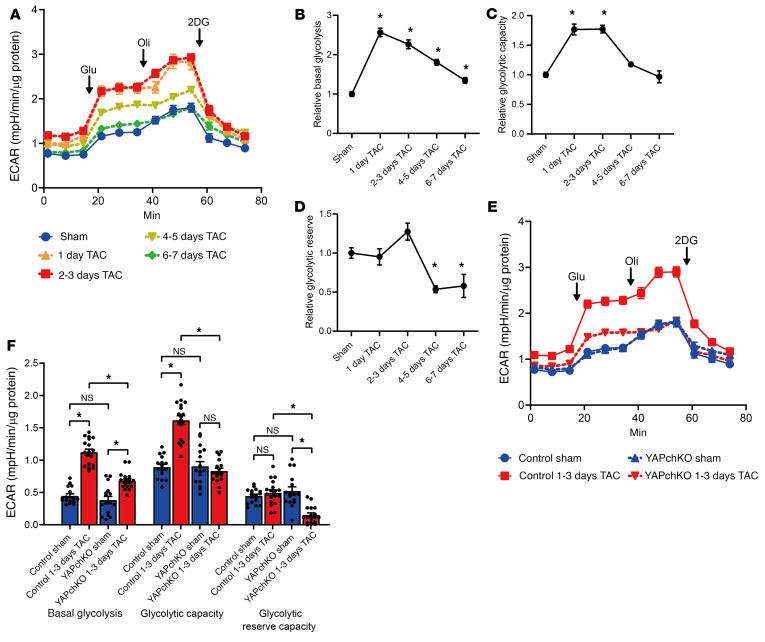
YAP promotes CM glycolysis in response to PO. (**A**–**D**) Time-course analysis of glycolytic flux after PO in freshly isolated AMVMs from control mice. Glycolysis was evaluated by measuring the ECAR. Overall ECAR in Seahorse experiments (**A**), relative basal glycolysis (**B**), relative glycolytic capacity (**C**), and relative glycolytic reserve capacity (**D**). *n =* 11 –20 wells from 3 mice at each time point. *P < 0.05 versus sham, by 1-way ANOVA with Dunnett’s test (**B**–**D**). (**E** and **F**) Haploinsufficiency of YAP in AMVMs attenuated PO-induced glycolysis. Overall Seahorse experiment (ECAR) (**E**) and summary of glycolytic function (**F**). *n =* 16 to 18 wells from 3 mice. *P < 0.05, by 1-way ANOVA with Tukey’s test (**F**). Data represent the mean ± SEM. Glu, glucose; Oli, oligomycin.

Since activation of glycolysis is intimately involved in cell growth, including cardiac hypertrophy (12, 14), we first tested whether YAP is involved in the activation of glycolysis during acute PO in the heart. Mice were subjected to transverse aortic constriction (TAC), which induces PO in the heart (3), for various durations. Using the Seahorse XF analyzer, we evaluated the extracellular acidification rate (ECAR) in freshly isolated adult mouse ventricular myocytes (AMVMs) as a measure of glycolysis. Both the relative basal glycolysis and relative glycolytic capacity, but not the relative glycolytic reserve capacity, were increased by TAC, peaking at 1–3 days, and the relative basal glycolysis remained elevated until 7 days after TAC (Figure 1, A–D). These changes corresponded to an upregulation of YAP in response to TAC (3). TAC-induced increases in relative basal glycolysis and relative glycolytic capacity at 1–3 days were significantly attenuated in YAPch-KO hearts (Figure 1, E and F), suggesting that endogenous YAP plays a critical role in mediating the activation of glycolysis in response to PO.

To determine which step or steps of glycolysis are stimulated in response to acute PO, we harvested hearts 2 days after TAC, when the left ventricular (LV) wall stress marker *Nppb* was increased (Supplemental Figure 1A; supplemental material available online with this article; https://doi.org/10.1172/JCI150595DS1) and YAP signaling was activated (Supplemental Figure 1, B–D, G–I, and Q), but neither control nor YAPch-KO mice developed signs of cardiac hypertrophy or heart failure (Supplemental Figure 1, E and F). We evaluated both mRNA and protein levels of transporters and enzymes of the glycolytic, auxiliary, and anaplerotic pathways in the heart (Supplemental Figure 2). Total YAP was significantly increased, whereas Ser127 phosphorylated YAP/total YAP was significantly decreased in Cre-negative control mice 2 days after TAC (Supplemental Figure 1, G–I and Q). In YAPch-KO mice, total YAP and Ser127 phosphorylated YAP/total YAP were significantly lower at baseline and did not change 2 days after TAC. Compensatory changes in the protein levels of WW domain–containing transcription regulator 1 (TAZ), a functional homolog of YAP, were not observed in YAPch-KO mice either at baseline or after TAC (Supplemental Figure 1, J and R). TAC significantly increased mRNA expression of *Glut1*, *Pgam1*, and *Pkm2* but decreased mRNA expression of *Glut4* and *Pkm1* in control mouse hearts (Supplemental Table 1 and Supplemental Figure 3). At the protein level, TAC only significantly altered glucose transporter 1 (GLUT1), which was upregulated 1.89-fold compared with the levels observed after sham operation, in the control mouse heart (Table 1, Supplemental Figure 4, and Supplemental Figure 5). In YAPch-KO hearts, *Glut4*, *Pkm1*, *Ldha*, *Pc*, and *Me1* mRNA levels were significantly lower at baseline, and *Glut4*, *Hk2*, *Pfk1*, *Gapdh*, *Pgk1*, *Pkm1*, *Mpc1*, *Mpc2*, *Ldha*, *Pdh*, *Pc*, and *Me1* mRNA levels were significantly lower after TAC, than were levels in control hearts at baseline. *Glut1*, *Pgam1*, *Ldha*, and *Me1* mRNA levels after TAC were significantly lower in YAPch-KO hearts than in control hearts (Supplemental Table 1 and Supplemental Figure 3). Interestingly, other than glucose transporter 4 (GLUT4), pyruvate dehydrogenase kinase 4 (PDK4), and malic enzyme 1 (ME1), glucose metabolic protein levels were not significantly altered in the YAPch-KO hearts at baseline or after TAC compared with levels in control hearts at baseline (Table 1, Supplemental Figure 4, and Supplemental Figure 5). Importantly, the TAC-induced upregulation of GLUT1 observed in the control mouse hearts was abolished in YAPch-KO hearts (Table 1, Supplemental Figure 4, and Supplemental Figure 5). These results suggest that endogenous YAP plays an essential role in mediating the upregulation of GLUT1, a major mechanism of glucose uptake and the first step of glycolysis, in response to PO.

**Figure 2:**
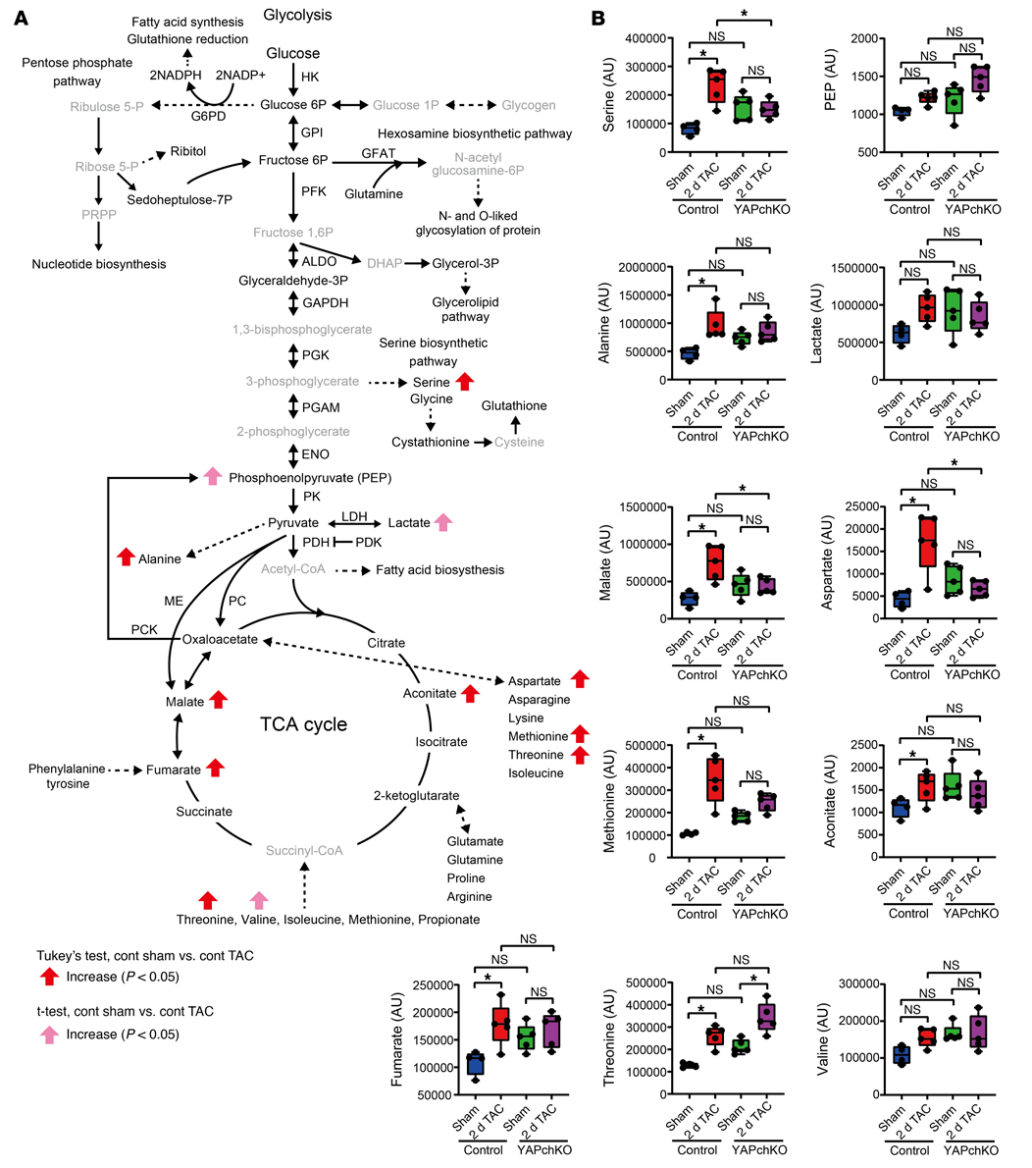
Metabolomics analysis of glucose metabolism in response to PO. (**A** and **B**) Summary of the metabolomics analysis of glucose metabolism after sham operation or 2 days of TAC in control and YAPch-KO mice. Black and gray indicate detectable and undetectable, respectively (**A**). Summary of intermediates of glucose metabolism, shown as box plots (**B**). *n =* 4–5 mice. *P < 0.05, by 1-way ANOVA with Tukey’s test. Data represent the mean ± SEM. See also Supplemental Table 2.

To evaluate how PO affects steady-state levels of the metabolic intermediates of the glycolytic, auxiliary, and anaplerotic pathways, we harvested hearts 2 days after TAC and conducted metabolomic analyses (Figure 2, A and, B, and Supplemental Table 2). TAC significantly (*P <* 0.05) increased the levels of l-serine, a metabolite of the serine biosynthetic pathway; malate, fumarate, aconitate, intermediates of the TCA cycle; and l-aspartate, l-alanine, l-methionine, and l-threonine in control mouse hearts. Phosphoenolpyruvate (PEP) and lactate, intermediates or metabolites of glycolysis, and l-valine were also slightly increased by TAC in the control hearts, although the increase did not reach statistical significance. Among the metabolites that were significantly elevated in response to TAC, the increases in l-serine, l-aspartate, and malate were significantly reversed in YAPch-KO mice subjected to TAC compared with in control mice subjected to TAC. These results suggest that PO induces the accumulation of intermediates of the glycolytic, auxiliary, and anaplerotic pathways and that endogenous YAP is critically involved in the accumulation of some, if not all, of the metabolic intermediates.

### YAP increases glycolysis in CMs in a cell-autonomous manner.

**Figure 3:**
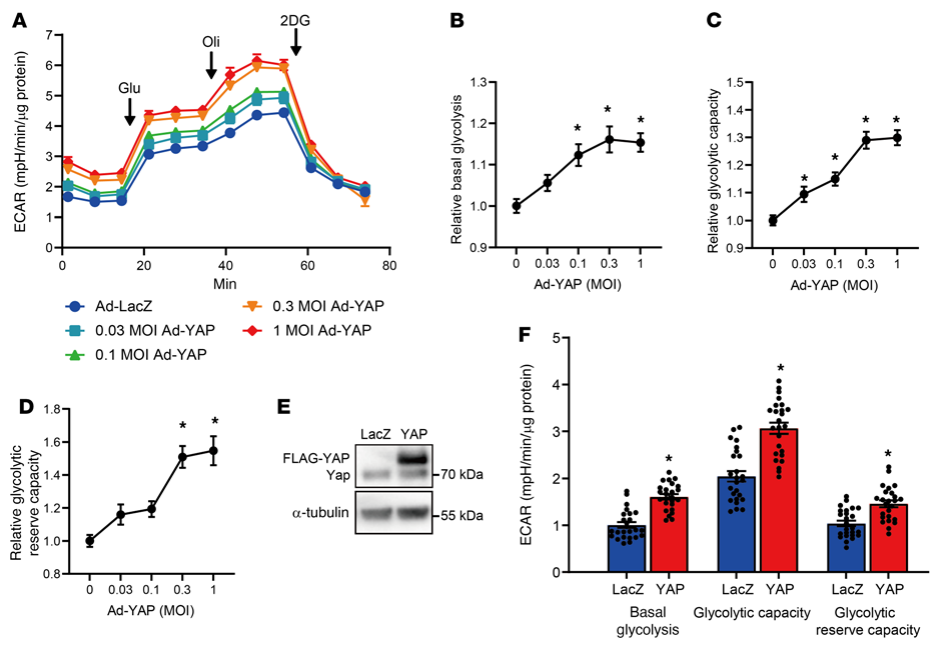
YAP facilitates glycolysis in CMs. (**A**–**D**) Seahorse glycolytic flux analysis was performed in NRVMs transduced with Ad-LacZ or Ad-FLAG-YAP for 6 days in serum-free DMEM/F12 medium. Overall ECAR in Seahorse experiments (**A**), relative basal glycolysis (**B**), relative glycolytic capacity (**C**), and relative glycolytic reserve capacity (**D**). *n =* 12 wells from 3 independent experiments. *P < 0.05 versus LacZ, by 1-way ANOVA with Dunnett’s test (**B**–**D**). (**E** and **F**) Confirmation of YAP expression (**E**) and a summary of glycolytic function in NRVMs transduced with 1 MOI Ad-FLAG-YAP (**F**). An α-tubulin blot, serving as a loading control, was run in parallel and contemporaneously with the other blot (**E**). *n =* 25 wells from 3 independent experiments. *P < 0.05 by 2-tailed, unpaired Student’s *t* test (**F**). Data represent the mean ± SEM.

To test whether YAP stimulates glycolysis in a cell-autonomous manner, we transduced cultured neonatal rat ventricular myocytes (NRVMs) with adenovirus (Ad) harboring either LacZ or YAP. Ad-YAP concentration-dependently increased relative basal glycolysis, glycolytic capacity, and glycolytic reserve capacity in NRVMs (Figure 3, A–D). Transduction of NRVMs with 1 MOI Ad-YAP caused an approximately 3-fold overexpression of YAP and significantly increased basal glycolysis, glycolytic capacity, and glycolytic reserve capacity (Figure 3, E and F).

Overexpression of YAP in NRVMs increased both mRNA (*Glut1*, *Glut4*, *Hk2*, *Gpi*, *Pfk1*, *Aldoa*, *Gapdh*, *Pgk1*, *Pgam1*, *Eno1*, *Pkm1*, *Ldha*, and *Pdh*) and protein (GLUT1, hexokinase 1 [HK1], HK2, glucose-6-phosphate isomerase [GPI], phosphofructokinase 1 [PFK1], aldolase A [ALDOA], GAPDH), phosphoglycerate kinase 1 (PGK1), 

phosphoglycerate mutase 1 (PGAM1), enolase 1 (ENO1), lactate dehydrogenase A (LDHA), and PDK4) levels of many glycolytic factors, whereas in the auxiliary pathway, protein expression of the enzymes, including glutamine-fructose-6-phosphate aminotransferase (GFAT), glycerol-3-phosphate dehydrogenase (GPD), and phosphoglycerate dehydrogenase (PHGDH), was not affected and that of glucose-6-phosphate dehydrogenase (G6PD) was decreased (Table 2 and Supplemental Figures 6 and 7). Overexpression of YAP in NRVMs decreased mRNA expression of components of the anaplerotic pathway (*Pc* and *Me1*) without affecting their protein levels of pyruvate carboxylase (PC) or ME1. shRNA-mediated downregulation of YAP in NRVMs significantly decreased basal glycolysis, glycolytic capacity, and glycolytic reserve capacity compared with in NRVMs expressing control shRNA, probably because of decreased expression of PGK1, PGAM1, and ENO1 proteins (Supplemental Figure 8 and Supplemental Table 3). Taken together, these results suggest that YAP induces aerobic glycolysis, namely the Warburg effect, in CMs.

**Figure 4:**
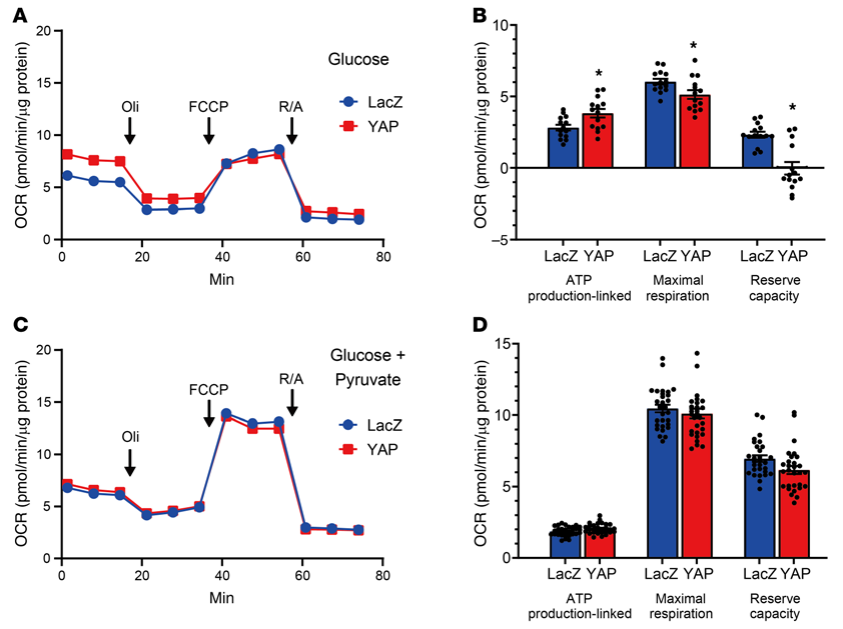
YAP increases glucose oxidation in NRVMs. (**A**–**D**) A Seahorse Mito Stress Test was performed in NRVMs transduced with Ad-LacZ or Ad-FLAG-YAP in 10 mM glucose medium in the absence (**A** and **B**) or presence (**C** and **D**) of 1 mM pyruvate. Overall Seahorse experiment (OCR) (**A** and **C**) and summary of mitochondrial function (**B** and **D**). *n =* 14 (**A** and **B**) and *n* = 31 (**C** and **D**) wells from 3 independent experiments. *P < 0.05, by 2-tailed, unpaired Student’s *t* test. Data represent the mean ± SEM.

Overexpression of YAP in NRVMs significantly increased the ATP production–linked oxygen consumption rate (OCR) when the medium contained 10 mM glucose, but not 10 mM glucose plus 1 mM pyruvate, suggesting that YAP-induced increases in glycolysis contribute to increased ATP production through aerobic oxidation of glucose (Figure 4, A–D). YAP did not increase the maximal respiration or mitochondrial complex proteins, except for mitochondrial complex II, in NRVMs (Figure 4, C and D, and Supplemental Figure 9), suggesting that YAP has negligible effect on oxidative phosphorylation.

### Metabolomic analyses in CMs overexpressing YAP.

**Figure 5:**
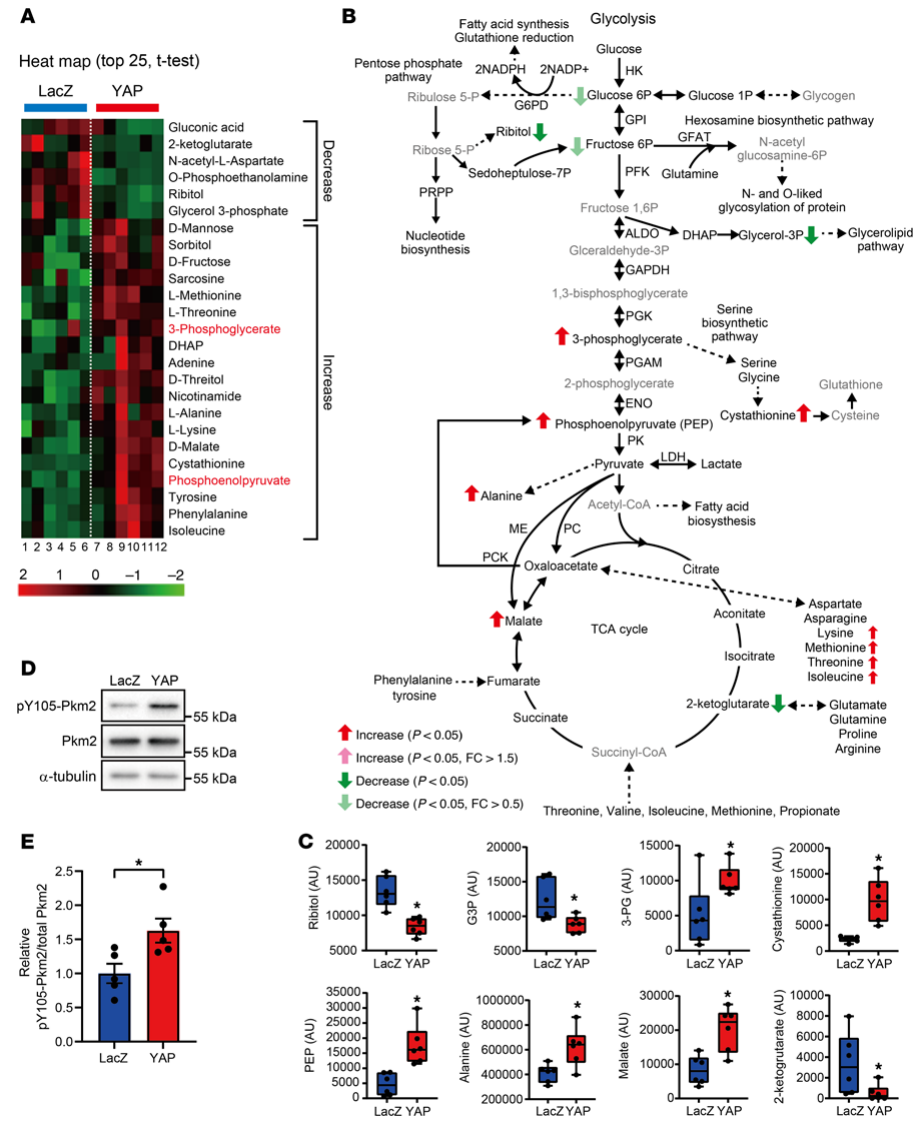
Glucose metabolism is regulated by YAP in CMs. (**A** and **B**) Metabolomics analysis of glucose metabolism was performed in NRVMs transduced with Ad-LacZ or Ad-FLAG-YAP for 6 days in serum-free DMEM/F12 medium. (**A**) Heatmap represents the expression profile of intermediates of glucose metabolism. (**B** and **C**) Summary of the metabolomics analysis of glucose metabolism. Black and gray represent detectable and undetectable, respectively (**B**). Box plots show a summary of intermediates of glucose metabolism (**C**). *n =* 6 dishes from 3 independent experiments. See also Supplemental Table 4. (**D** and **E**) YAP increased Tyr105 phosphorylated Pkm2 in NRVMs transduced with Ad-LacZ or Ad-FLAG-YAP. Representative immunoblots (**D**) and a summary of quantification (**E**) are shown. α-Tubulin and PKM2 blots, serving as loading controls, were run in parallel and contemporaneously with other blots (**D**). *n =* 5 dishes from 5 independent experiments. *P < 0.05, by 2-tailed, unpaired Student’s *t* test (**C** and **E**). Data represent the mean ± SEM.

To investigate how the activation of YAP affects the CM metabolic landscape, we conducted metabolomic analyses using cultured NRVMs transduced with either Ad-LacZ or Ad-YAP (Supplemental Table 4). Several metabolic intermediates in the upstream glycolytic pathway, including glucose-6-phosphate and fructose-6-phosphate, and products of the auxiliary pathways, including ribitol and glycerol-3-phosphate, were lower in Ad-YAP–transduced CMs than in Ad-LacZ–transduced CMs (Figure 5, A–C). In contrast, metabolic intermediates in the downstream glycolytic pathway, including PEP and 3-phosphoglycerate (3-PG), malate, cystathionine (a serine biosynthetic pathway product), l-alanine, and amino acids synthesized from l-aspartate, including l-lysine, l-methionine, l-threonine, and l-isoleucine, were significantly elevated in Ad-YAP–transduced CMs compared with levels in Ad-LacZ–transduced CMs (Figure 5, A–C). It has been suggested that a less active form of pyruvate kinase M2 (PKM2) causes accumulation of upstream intermediates of the glycolytic pathway, thereby increasing substrate availability for the auxiliary pathways (13). Phosphorylation of PKM2 at Tyr105, which reduces PKM2 activity and induces cell growth, was significantly induced by YAP in CMs (Figure 5, D and E).

**Figure 6:**
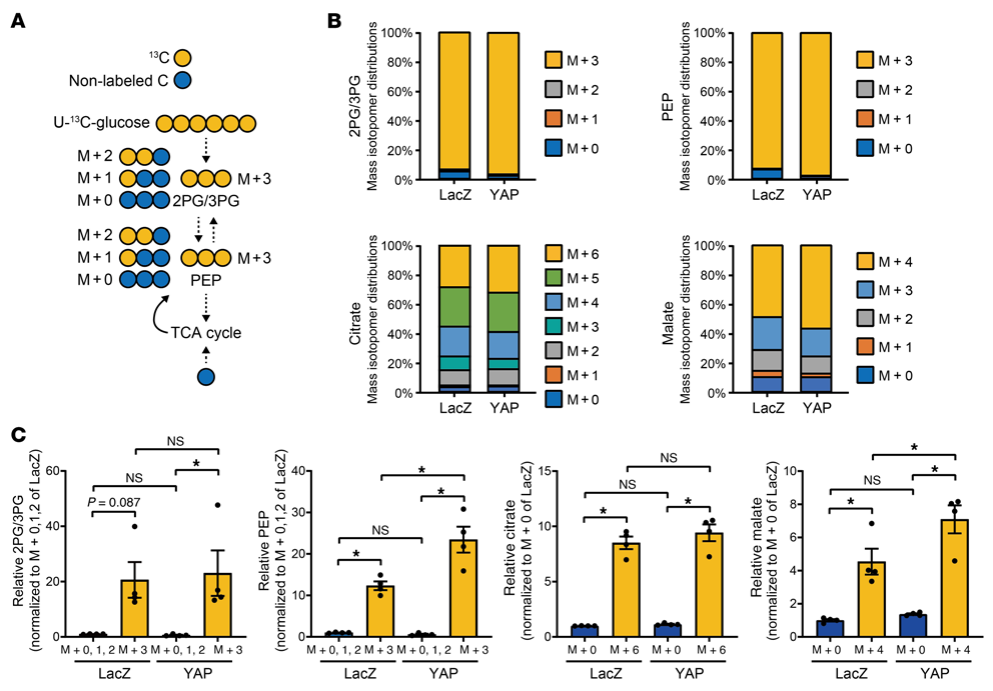
Overexpressed YAP increases PEP and malate through glycolysis. NRVMs were transduced with Ad-LacZ or Ad-FLAG-YAP for 5 days in serum-free DMEM/F12 medium, and then the medium was replaced with DMEM containing U-^13^C-glucose. (**A**–**C**) Schematic of metabolism of U-^13^C-glucose (**A**), mass isotopomer distributions of 2-PG/3-PG, PEP, citrate, and malate (**B**), and relative levels of 2-PG/3-PG, PEP, citrate, and malate (**C**). *n =* 4 dishes from 2 independent experiments. *P < 0.05, by 1-way ANOVA with Tukey’s test. Data represent the mean ± SEM.

To evaluate whether glycolysis contributes to the YAP-induced accumulation of metabolic intermediates detected above, we conducted stable isotope–labeling experiments using uniformly labeled ^13^C-glucose (U-^13^C-glucose). Approximately 94% of 2-PG/3-PG (M+3) and 93% of PEP (M+3) were derived from extracellular glucose in cultured NRVMs transduced with Ad-LacZ, and the proportion and amount of PEP (M+3) were increased significantly in the presence of YAP overexpression (Figure 6, A–C). The proportion and amount of malate (M+4), whose carbons are highly dependent on extracellular glucose, were also significantly increased in the presence of YAP overexpression (Figure 6, B and C). These results suggest that YAP induces the accumulation of PEP and malate by promoting glycolysis and/or the anaplerotic pathway (15).

PEP, malate, the serine biosynthetic pathway, l-alanine, and amino acids synthesized from l-aspartate were upregulated in both mouse hearts after TAC in vivo (Figure 2B) and cultured NRVMs overexpressing YAP in vitro (Figure 5C). Thus, these intermediates may be involved in the protective effect of YAP in the heart during acute PO.

### YAP-induced cardiac hypertrophy in cultured neonatal CMs is inhibited by the downregulation of glucose metabolic genes.

**Figure 7:**
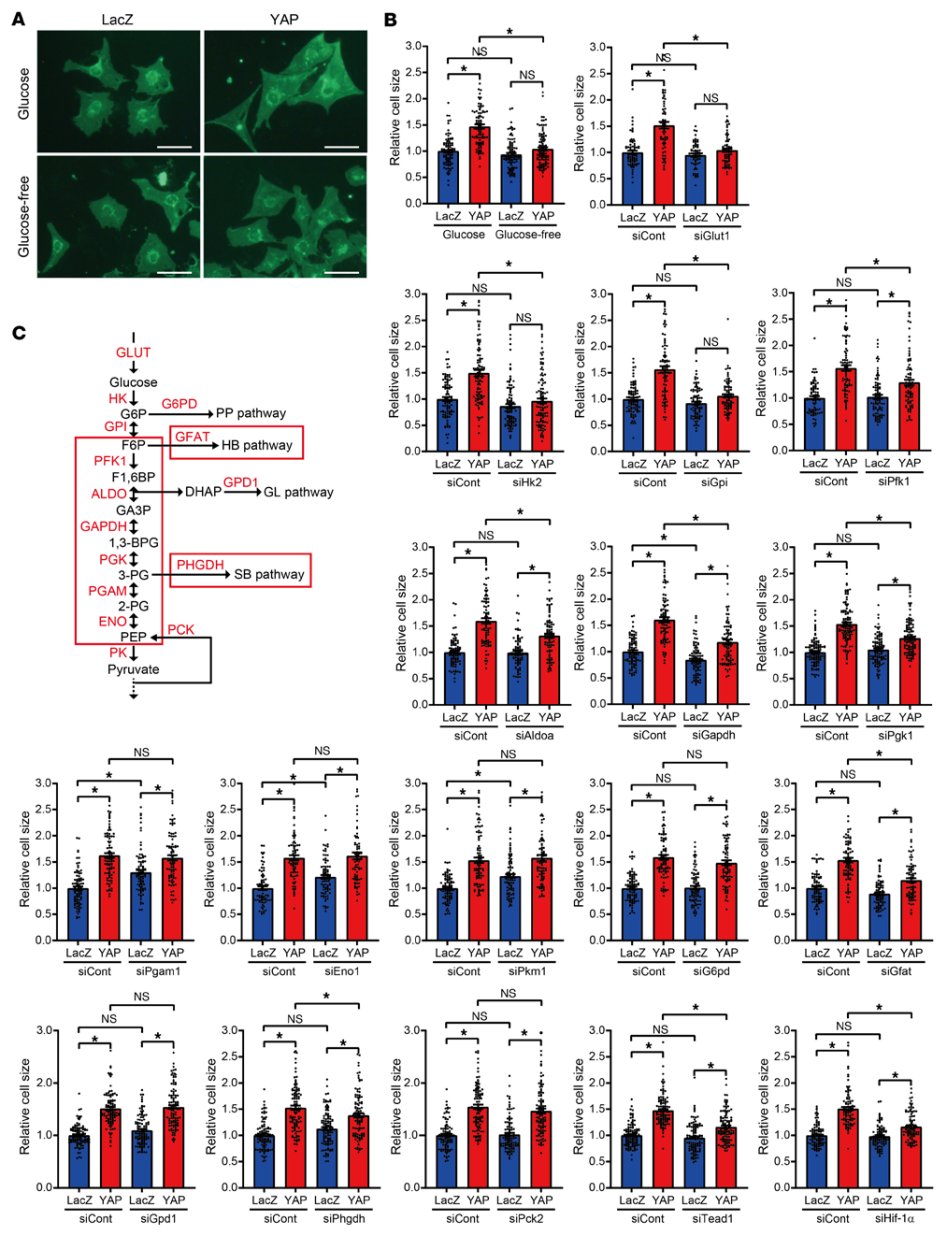
Increased glycolytic flux is essential for YAP-mediated cardiac hypertrophy. Cardiac hypertrophy was evaluated with WGA staining. (**A**) Representative images of NRVMs transduced with Ad-LacZ or Ad-FLAG-YAP in glucose-free or normal DMEM medium for 5 days are shown. Scale bars: 50 μm. (**B**) Effect of knockdown of glucose metabolic genes on YAP-mediated cardiac hypertrophy. *n =* 63–113 cells from 3 independent experiments. *P < 0.05, by 1-way ANOVA with Tukey’s test. Data represent the mean ± SEM. See also Supplemental Figure 10, which shows knockdown of glucose metabolic genes confirmed by immunoblotting. siCont, siControl. (**C**) Glycolytic pathways, indicated by red boxes, are required for YAP-mediated cardiac hypertrophy.

Cardiac hypertrophy is at least initially an adaptive response of the heart against acute PO. Hypertrophy of individual CMs increases wall thickness, thereby reducing wall stress according to the law of Laplace, which in turn reduces oxygen consumption and cell death. Thus, inducing adaptive cardiac hypertrophy is key to maintaining cardiac function during acute PO (1). We therefore investigated whether the Warburg effect mediates cardiac hypertrophy in response to YAP activation. We previously showed that YAP overexpression induces cardiac hypertrophy in NRVMs (16). We tested whether siRNA-mediated downregulation of glucose metabolic genes inhibits YAP-induced cardiac hypertrophy (Figure 7, A–C, and Supplemental Figure 10). The effects of siRNA-mediated downregulation upon YAP-induced hypertrophy could be classified into 4 groups. Downregulation of GLUT1, HK2, or GPI completely inhibited YAP-induced cardiac hypertrophy, as indicated by cell size (group 1). Similar results were obtained when cells were subjected to glucose-free medium (Figure 7, A and B). Downregulation of PFK1, ALODA, GAPDH, or PGK1 significantly inhibited YAP-induced hypertrophy; however, a modest but significant increase in CM size was observed under these conditions (group 2). Downregulation of PGAM1, ENO1, or PKM1 increased baseline CM size but did not affect YAP-induced increases in CM size (group 3). Finally, downregulation of G6PD, a key enzyme on the pentose phosphate pathway, GPD1, an enzyme in the glycerolipid pathway, or phosphoenolpyruvate carboxykinase (PCK2), an enzyme in the cataplerotic pathway, did not affect either baseline or YAP-induced hypertrophy (group 4). In contrast, downregulation of GFAT, a key enzyme in the hexosamine biosynthetic pathway, or PHGDH, a key enzyme on the serine biosynthetic pathway, had an effect similar to that observed in group 2. Together, these results suggest that glycolytic intermediates located below GPI (F6P–pyruvate) and the hexosamine and serine biosynthetic pathways may be involved in cardiac hypertrophy (Figure 7C). Metabolomic studies indicated that 3-PG, PEP, and intermediates of the serine biosynthetic pathway were increased in the presence of YAP (Figure 2B and Figure 5C). Accumulation of PEP could supplement the level of 3-PG through the reversible actions of ENO and PGAM. These results suggest that the accumulation of PEP, 3-PG, and metabolic intermediates on the serine biosynthetic pathway mediates YAP-induced cardiac hypertrophy.

### YAP stimulates the serine biosynthetic pathway.

Serine synthesis through the serine biosynthetic pathway promotes cell growth and survival by producing proteins and nucleotides, S-adenosyl methionine (SAM), and glutathione (GSH) (17). YAP upregulated trimethylation at the fourth lysine residue of histone H3 (H3K4me3), modulated by SAM, and GSH (Supplemental Figure 11). Both were inhibited by CBR-5884, a selective PHGDH inhibitor, in cultured NRVMs (ref. 18 and Supplemental Figure 11). Thus, the serine biosynthetic pathway is activated in response to YAP and may contribute to YAP-induced CM growth and survival. Interestingly, metabolomic analyses showed that l-serine accumulated in the heart 2 days after TAC in a YAP-dependent manner (Figure 2B).

### YAP induces hypertrophy through GLUT1-mediated glycolysis in cultured adult CMs.

**Figure 8:**
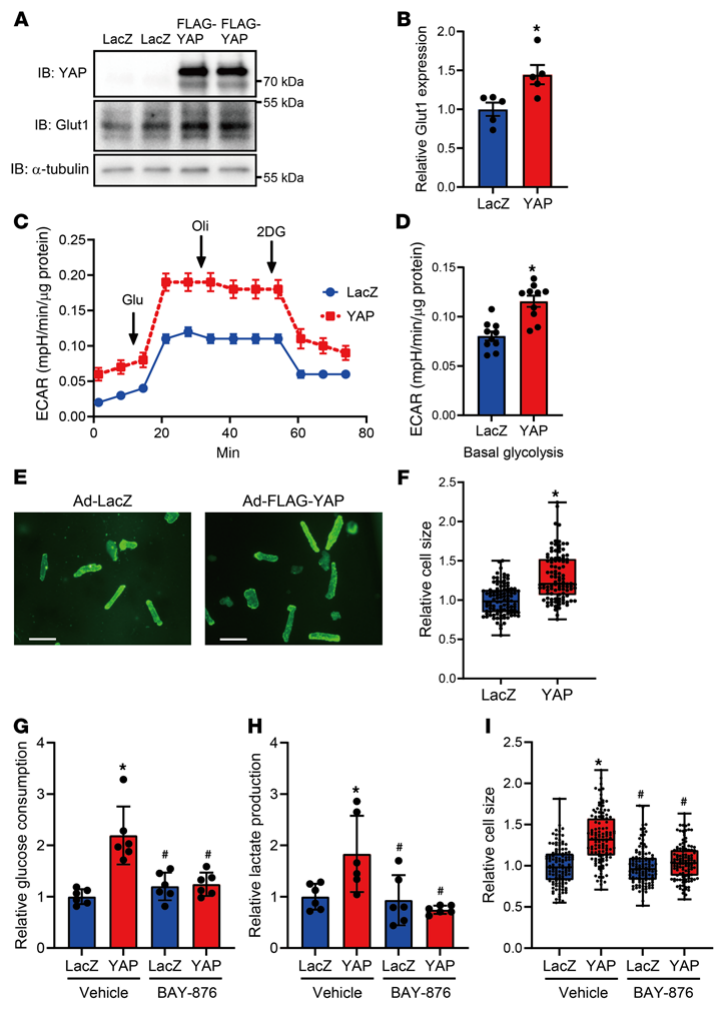
YAP induces cardiac hypertrophy through GLUT1-mediated glycolysis in adult CMs. (**A**–**I**) Overexpressed YAP induced hypertrophy in AMVMs, accompanied by the upregulation of GLUT1 expression and glycolysis. AMVMs were transduced with 0.1 MOI Ad-LacZ or Ad-FLAG-YAP for 2 or 4 days. Representative immunoblots (**A**) and quantification results (**B**) after 4 days of culturing. An α-tubulin blot, serving as a loading control, was run in parallel and contemporaneously with the other blots (**A**). *n =* 5 dishes from 3 mice. Overall ECAR (**C**) and summary of basal glycolysis (**D**) after 2 days of culturing. *n =* 10 wells from 3 mice. Representative images of WGA staining (**E**) and summary of relative CM sizes (**F**) after 4 days of culturing. Scale bars: 100 μm. *n =* 101 cells from 3 mice. *P < 0.05, by 2-tailed, unpaired Student’s *t* test (**B**, **D**, and **F**). (**G**–**I**) YAP-induced glycolysis and hypertrophy were inhibited by 20 nM BAY-876, a selective GLUT1 inhibitor, in cultured AMVMs. Glucose consumption (**G**) and lactate production (**H**) in media after 48 hours of culturing were measured in AMVMs transduced with Ad-LacZ or Ad-FLAG-YAP in the presence or absence of BAY-876. *n =* 6 wells from 6 mice. (**I**) CM size was evaluated with WGA staining after 4 days of culturing. *n =* 120 cells from 6 mice. *P < 0.05 versus LacZ plus vehicle and ^#^*P <* 0.05 versus YAP plus vehicle, by 1-way ANOVA with Tukey’s test (**G**–**I**). Data represent the mean ± SEM (**B**–**D**, **G**, and **H**). Results in **F** and **I** are shown as box plots.

To investigate whether YAP activation induces GLUT1-mediated glycolysis and hypertrophy in adult CMs, we transduced cultured AMVMs with Ad-LacZ or Ad-YAP. Ad-YAP transduction significantly increased GLUT1 protein levels, basal glycolysis, and CM size compared with Ad-LacZ transduction (Figure 8, A–F). YAP overexpression also increased both glucose consumption and lactate production (Figure 8, G and H). BAY-876, a selective GLUT1 inhibitor, almost completely inhibited YAP-induced glucose consumption, lactate production, and hypertrophy in AMVMs (Figure 8, G–I). These results suggest that YAP-mediated upregulation of GLUT1 induces hypertrophy through glycolysis in adult CMs.

### Genetic rescue of GLUT1 alleviates cardiac dysfunction in YAPch-KO mice during acute PO.

**Figure 9:**
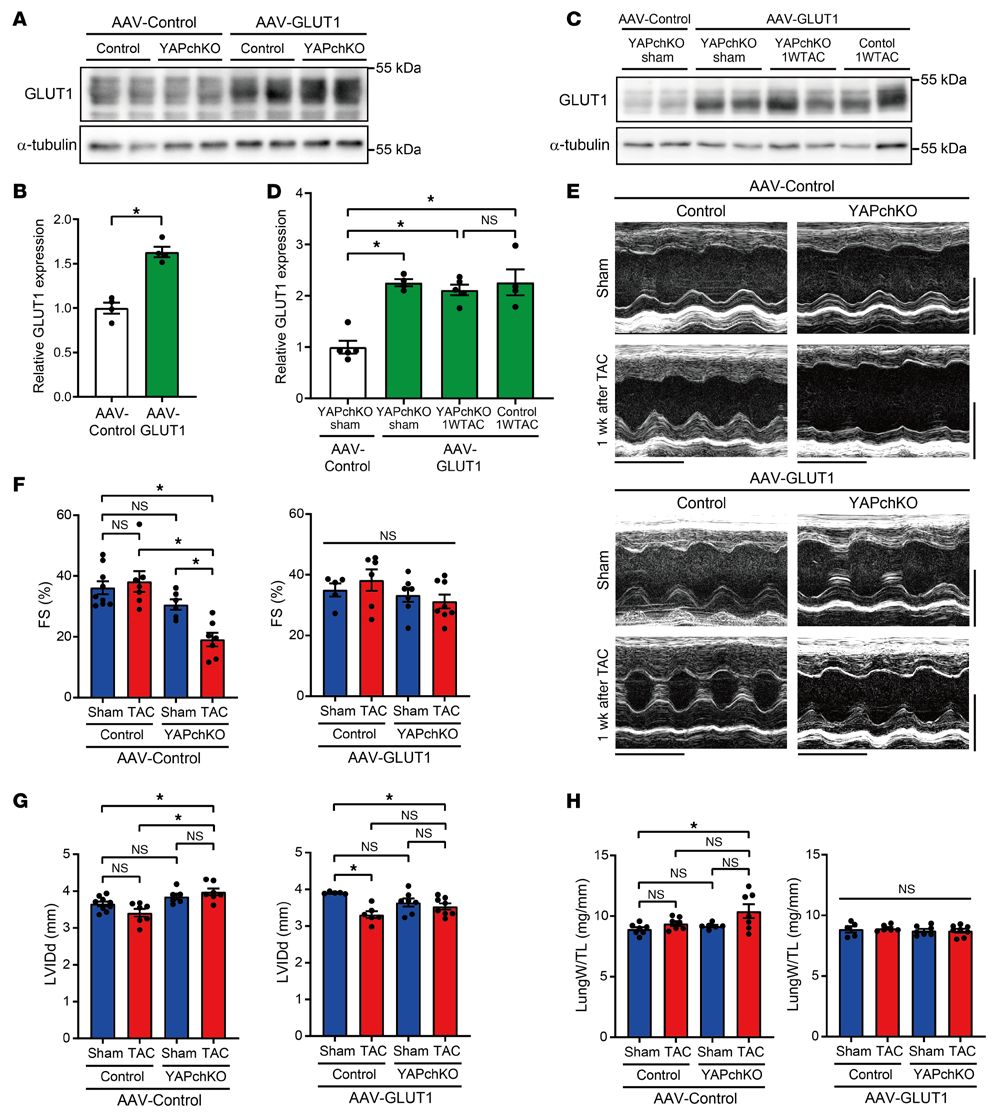
Overexpression of GLUT1 ameliorates cardiac dysfunction in YAPch-KO mice during acute PO. (**A**–**D**) AAV-GLUT1 injection increased GLUT1 expression in mouse hearts. LVs from control and YAPch-KO mice 2 weeks after injection of AAV-control or AAV-GLUT1 (*n =* 4 mice) (**A** and **B**) and sham operation or 1 week of TAC after injection of AAV-control or AAV-GLUT1 (*n =* 4–5 mice) (**C** and **D**) were homogenized and subjected to immunoblotting. α-Tubulin blots, serving as loading controls, were run in parallel and contemporaneously with other blots (**A** and **C**). (**E**–**G**) Cardiac function was evaluated by echocardiography 1 week after TAC. Representative M-mode echocardiographic images (**E**), FS (**F**), and LVIDd (**G**). Vertical scale bars: 3.5 mm; horizontal scale bars: 200 ms. *n =* 6–9 mice. (**H**) LungW/TL. *n =* 5–8 mice. *P < 0.05, by 2-tailed, unpaired Student’s *t* test (**B**) or 1-way ANOVA with Tukey’s test (**D** and **F**–**H**). Data represent the mean ± SEM.

**Figure 10:**
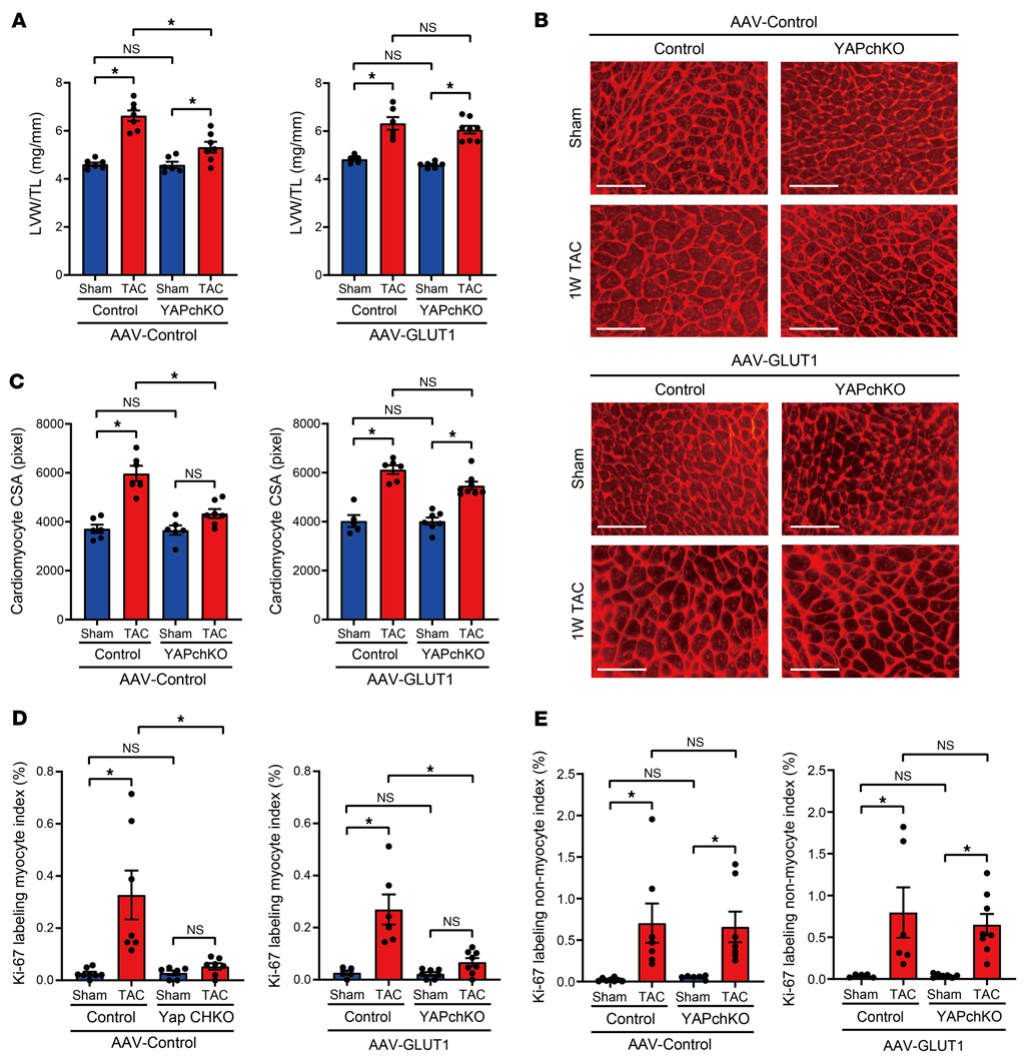
Overexpression of GLUT1 rescues compensatory cardiac hypertrophy in YAPch-KO mice during acute PO. AAV-GLUT1 injection rescued cardiac hypertrophy in YAPch-KO mice 1 week after TAC. (**A**) LVW/TL. *n =* 5–8 mice. (**B** and **C**) Representative images of WGA staining of LVs (**B**) and quantification of CM CSA (**C**). Scale bars: 50 μm. *n =* 5–8 mice. (**D** and **E**) AAV-GLUT1 injection did not alter the cell proliferation index of CMs or non-CMs in LVs. Heart sections were stained with anti–Ki-67 and anti–cardiac troponin T antibodies and DAPI. Percentage of Ki-67 labeling in CMs (*n =* 5–8 mice) (**D**) and non-CMs (*n =* 5–8 mice) (**E**). *P < 0.05 by 1-way ANOVA with Tukey’s test. Data represent the mean ± SEM.

Since PO-induced GLUT1 upregulation was inhibited in YAPch-KO mice in vivo and YAP induced hypertrophy through the upregulation of GLUT1 in cultured CMs, we hypothesized that GLUT1 downregulation is critical in mediating the detrimental effect of YAPch-KO on the heart during acute PO. We therefore conducted a GLUT1 rescue experiment, in which mice were injected with either adeno-associated virus–control (AAV-control) or AAV-GLUT1 and, after 2 weeks, subjected to either sham operation or TAC. We confirmed that AAV-GLUT1 significantly increased GLUT1 expression in the heart (1.6-fold, *P <* 0.05; Figure 9, A and B). After 7 days of PO, mice underwent echocardiographic analyses and were sacrificed. We confirmed that AAV-GLUT1 successfully rescued GLUT1 upregulation in YAPch-KO mice 7 days after TAC (Figure 9, C and D). Fractional shortening (FS, %), a measure of systolic function (Figure 9, E and F), LV end-diastolic internal dimension (LVIDd), a measure of LV dilation (Figure 9, E and G), and lung weight/tibia length (LungW/TL), a measure of lung congestion (Figure 9H), which is a sign of heart failure, were not affected in either AAV-control– or AAV-GLUT1–injected control mice. TAC-induced LV systolic dysfunction and LV dilation were observed in YAPch-KO mice injected with AAV-control (Figure 9, E–G), consistent with our previous results (3). In YAPch-KO mice injected with AAV-GLUT1, TAC-induced systolic dysfunction, LV dilation, and lung congestion were not significant (Figure 9, E–H). We previously showed that TAC for 7 days induces cardiac hypertrophy and cell cycle re-entry in CMs (3). We confirmed that TAC induced cardiac hypertrophy, as indicated by LV weight/tibia length (LVW/TL) (Figure 10A) and the histologically evaluated LV CM cross-sectional area (CSA), in control mice injected with AAV-control (Figure 10, B and C). Although TAC-induced cardiac hypertrophy was significantly inhibited in YAPch-KO mice injected with AAV-control, the suppression was not significant in YAPch-KO mice injected with AAV-GLUT1 (Figure 10, A–C). We also confirmed that TAC significantly stimulated cell cycle re-entry, indicated by Ki67 labeling, in CMs and non-CMs in the LV of control mice injected with AAV-control, whereas cell cycle re-entry of CMs, but not non-CMs, was suppressed in YAPch-KO mice injected with AAV-control (Figure 10, D and E). However, TAC-induced cell cycle re-entry in YAPch-KO CMs was not rescued by AAV-GLUT1 injection (Figure 10D). These results suggest that YAP-induced upregulation of GLUT1 plays a critical role in protecting the heart against acute PO, which is likely mediated through stimulation of compensatory cardiac hypertrophy but not CM cell cycle re-entry.

### HIF-1α and TEAD1 are involved in YAP-induced GLUT1 transcription.

**Figure 11:**
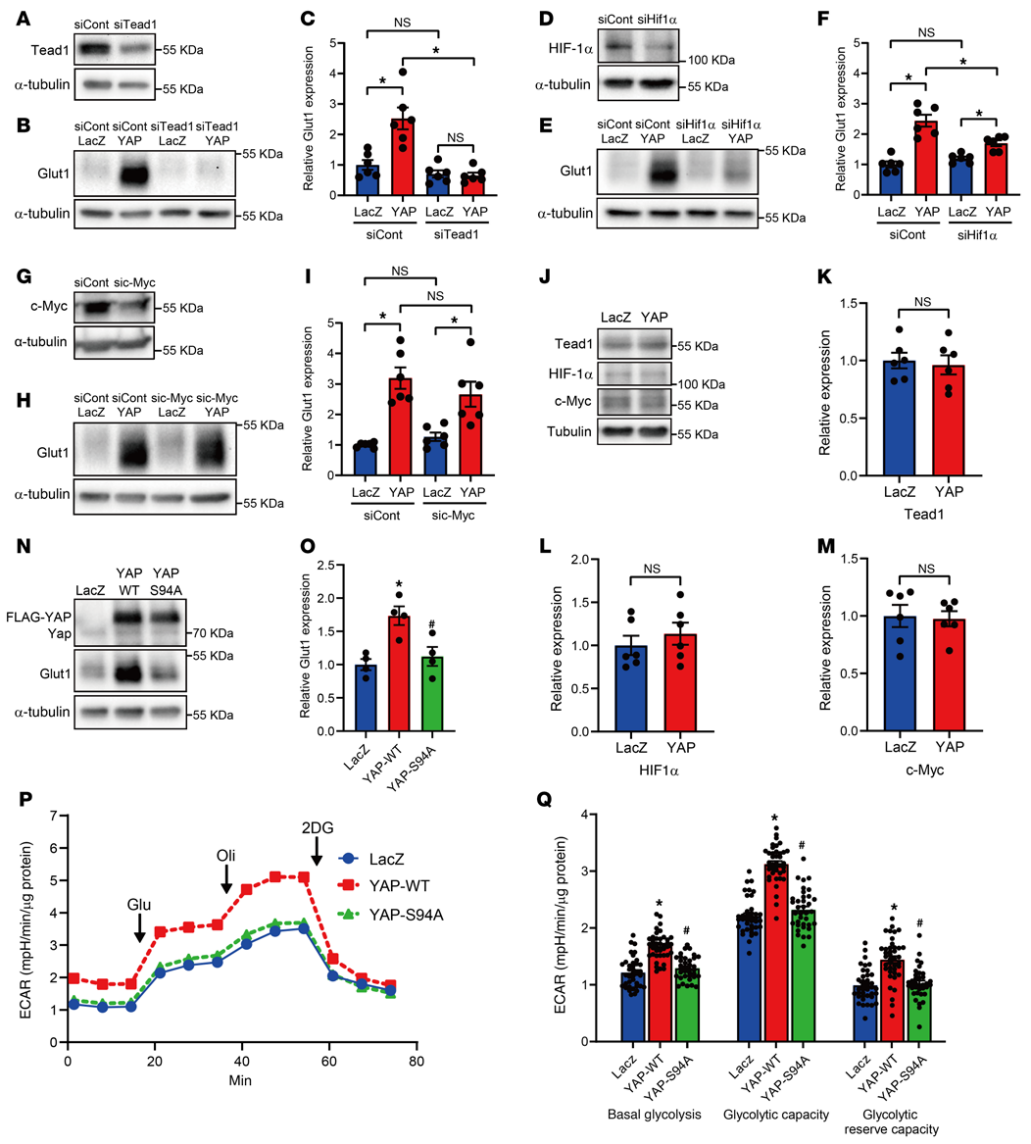
TEAD1 and HIF-1α are required for YAP-induced GLUT1 expression in CMs. (**A**–**I**) The effect of knockdown of TEAD1 (**A**–**C**), HIF-1α (**D**–**F**), or c-Myc (**G**–**I**) on YAP-induced GLUT1 expression in NRVMs. *n =* 6 dishes from 3 independent experiments. *P < 0.05, by 1-way ANOVA with Tukey’s test (**A**–**I**). (**K**–**M**) Representative immunoblots (**J**) and expression levels of TEAD1 (**K**), HIF-1α (**L**), and c-Myc (**M**) in NRVMs. *n =* 6 dishes from 3 independent experiments. *P < 0.05, by 2-tailed, unpaired Student’s *t* test (**J**–**M**). (**N**–**Q**) NRVMs were transduced with Ad-LacZ, Ad-FLAG-YAP-WT, or Ad-FLAG-YAP-S94A. Representative immunoblots (**N**) and expression levels of GLUT1 (**O**) are shown. *n =* 4 dishes from 4 independent experiments. Overall ECAR (**P**) and summary (**Q**). *n =* 36–40 wells from 3 independent experiments. *P < 0.05 versus LacZ and ^#^*P <* 0.05 versus YAP-WT, by 1-way ANOVA with Tukey’s test (**O** and **Q**). α-Tubulin blots, serving as loading controls, were run in parallel and contemporaneously with other blots (**A**, **B**, **E**, **G**, **H**, **J**, and **N**). Data represent the mean ± SEM.

Although GLUT1 expression is low in the normal adult heart, its expression is upregulated in the heart in response to hypoxia and hypertrophic stimuli (19, 20). Previous studies have shown that GLUT1 transcription is regulated by TEA domain family member 1 (TEAD1), HIF-1α, and c-Myc in proliferative cells like cancer cells and fibroblasts (21–23). YAP-induced upregulation of GLUT1 protein was not affected by control siRNA in NRVMs (Figure 11, A–I). However, YAP-induced GLUT1 upregulation was abolished or significantly inhibited by siRNA-mediated knockdown of TEAD1 or HIF-1α, respectively (Figure 11, A–F), but it was not significantly affected by knockdown of c-Myc (Figure 11, G–I). YAP overexpression did not significantly alter TEAD1 or HIF-1α protein levels in NRVMs (Figure 11, J–M). Although YAP overexpression upregulates GLUT1 in cultured NRVMs in vitro, YAP-S94A, a mutant that cannot interact with TEAD1 (24), failed to upregulate GLUT1 or stimulate glycolysis, as indicated by the ECAR (Figure 11, N–Q). Thus, YAP-TEAD1 interaction is essential for YAP-induced upregulation of glycolysis.

**Figure 12:**
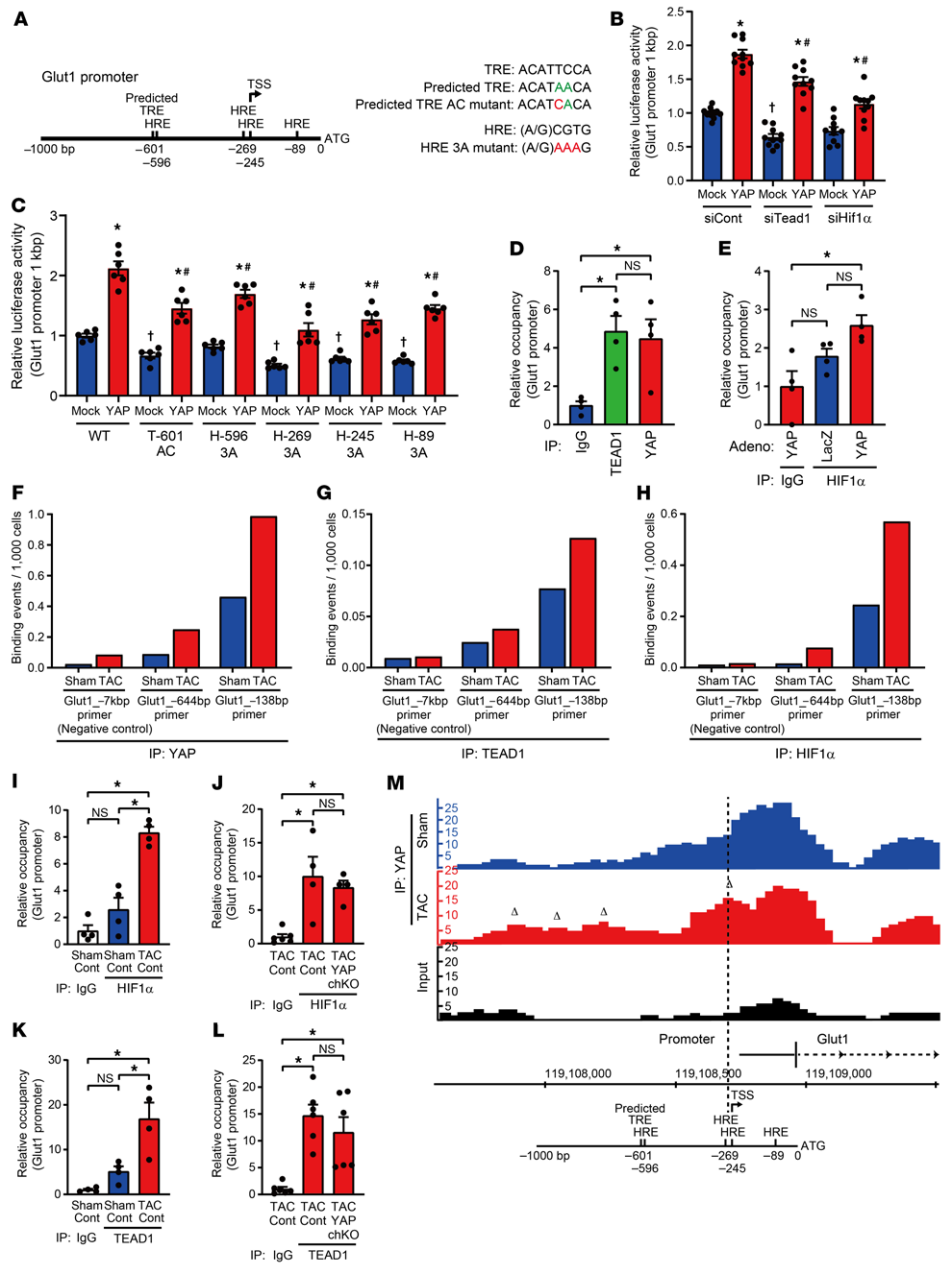
YAP, TEAD1, and HIF-1α bind to the *Glut1* promoter in the heart. (**A**) Schematic representation of HREs and the predicted TRE in the mouse *Glut1* promoter. The predicted TRE differs in 2 base pairs (green) from the consensus TRE. Point mutations are indicated in red. (**B** and **C**) Effects of siRNA against *Tead1* or *Hif1a* (**B**) and *Glut1* promoter mutations (**C**) on YAP-induced activation of the *Glut1* promoter. *n =* 6–10 wells from 3–4 independent experiments. *P < 0.05 versus each mock; ^#^*P <* 0.05 versus each control YAP; and †*P <* 0.05 versus control mock, by 1-way ANOVA with Tukey’s test. (**D** and **E**) ChIP assays of the *Glut1* promoter were performed in NRVMs using the indicated antibodies. *n =* 4 dishes from 2 independent experiments. *P < 0.05, by 1-way ANOVA with Tukey’s test. (**F**–**H**) ChIP assays of the *Glut1* promoter were performed using pooled hearts from WT mice after 2 days of sham operation or TAC, with antibodies against YAP (**F**), TEAD1 (**G**), and HIF-1α (**H**). (**I**–**L**) ChIP assays of the *Glut1* promoter were performed using hearts from control or YAPch-KO mice after 2 days of sham operation or TAC, with antibodies against HIF-1α (**I** and **J**) and TEAD1 (**K** and **L**). *n =* 4–6 mice. *P < 0.05, by 1-way ANOVA with Tukey’s test. (**M**) ChIP-Seq was performed using pooled hearts from WT mice subjected to sham operation or 4 days of TAC with anti-YAP antibody. Schematic representation of the *Glut1* promoter is aligned with the results of ChIP-Seq in the *Glut1* promoter. Triangles indicate new peaks after PO. ATG, start codon. Data represent the mean ± SEM.

The mouse *Glut1* proximal promoter contains 4 putative HIF-1α–responsive elements (HREs) and a TEAD-responsive element (TRE), and one of the HREs is located near the TRE (Figure 12A). To examine the roles of the HREs and the TRE, we generated luciferase reporter genes containing 1 kbp of the proximal promoter of the *Glut1* gene with mutations in either the TRE or one of the HREs (Figure 12A). The WT reporter gene activity in NRVMs was increased by YAP but significantly attenuated by either si*Tead1* or si*Hif1a* (Figure 12B), suggesting that both TEAD1 and HIF-1α play an essential role in mediating YAP-induced activation of the *Glut1* proximal promoter. A point mutation in the TRE at –601 or the HREs at –596, –269, –245, or –89 significantly decreased reporter gene activity at baseline and in the presence of YAP compared with the WT (Figure 12C). These results suggest that both the TRE and the HREs in the proximal promoter are involved in the YAP-induced transcriptional activation of *Glut1*. Downregulation of TEAD1 or HIF-1α also inhibited the YAP-induced increases in cell size in NRVMs (Figure 7B).

To test whether YAP, TEAD1, and HIF-1α interact with the *Glut1* promoter, we conducted ChIP assays with cultured NRVMs. ChIP assays with anti-TEAD1 or anti-YAP antibody showed significant binding to the *Glut1* promoter (Figure 12D). ChIP with anti–HIF-1α antibody also showed significant binding to the *Glut1* promoter when YAP was overexpressed (Figure 12E). These results are consistent with the notion that HIF-1α, TEAD1, and YAP interact with the *Glut1* promoter in cultured NRVMs.

We also conducted ChIP assays and ChIP-Seq analyses of mouse hearts after sham operation or TAC for 2–4 days, using the indicated antibodies (Figure 12, F–M). The experiments shown in Figure 12, F–H, and M were conducted at Active Motif, and 3 hearts were combined for each sample. YAP, TEAD1, and HIF-1α accumulated in the proximal promoter region approximately 138 bp upstream of the *Glut1* start codon in WT mice in response to TAC (Figure 12, F–H). We also repeated ChIP–quantitative PCR (qPCR) experiments ourselves using 4–6 hearts from control or YAPch-KO mice subjected to either sham operation or TAC. Binding of HIF-1α to the proximal *Glut1* promoter in response to TAC was not significantly decreased in YAPch-KO mice compared with that seen in control mice (Figure 12, I and J). TEAD1 binding tended to be decreased in YAPch-KO mice compared with control mice, but the difference did not reach statistical significance (Figure 12, K and L). Following TAC, new YAP binding peaks appeared in the proximal *Glut1* promoter containing the HRE (Figure 12M), as well as in the distal promoter and/or the enhancer. Together, these results suggest that acute PO induces interaction between YAP/TEAD1/HIF-1α and the proximal promoter region of *Glut1*, including the HRE, and possibly between YAP/TEAD1/HIF-1α and the enhancer region.

### A protein complex consisting of TEAD1, YAP, and HIF-1α is formed on the GLUT1 promoter.

**Figure 13:**
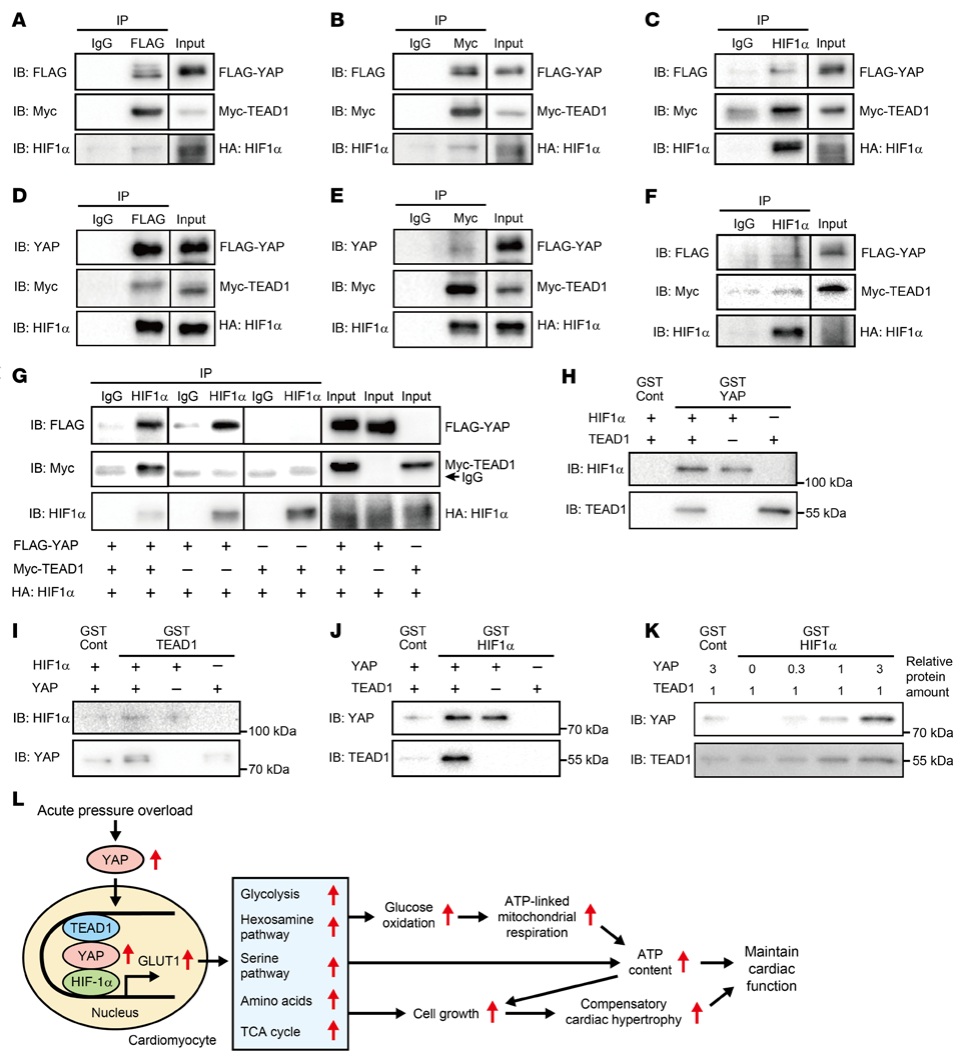
YAP physically interacts with both TEAD1 and HIF-1α. (**A**–**C** and **G**) HEK293 cells were transfected with plasmids encoding FLAG-YAP, Myc-TEAD1, and HA–HIF-1α P402A/P564A, a stable HIF-1α mutant. (**D**–**F**) NRVMs were transduced with Ad-FLAG-YAP, Ad-Myc-TEAD1, and Ad-HA–HIF-1α P402A/P564A. The cells were treated with 3 μM MG-132 twenty-four hours prior to being harvested. Immunoblotting (IB) and immunoprecipitation (IP) were performed using the indicated antibodies. Data are representative of 3 independent blots. The lanes were run on the same gel but were noncontiguous (**A**–**G**). (**H**–**K**) YAP directly bound to both TEAD1 and HIF-1α, while TEAD1 and HIF-1α only interacted with one another through YAP. The indicated GST-fused proteins and recombinant proteins were incubated, followed by pulldown with glutathione-sepharose beads. Data are representative of 3 independent blots. (**L**) Schematic illustration of the role of YAP in regulating CM glycolysis in the heart during acute PO.

To investigate whether TEAD1, YAP, and HIF-1α interact with one another, we performed co-immunoprecipitation experiments using HEK293 cells and NRVMs overexpressing FLAG-YAP, Myc-TEAD1, and HA–HIF-1α P402A/P564A, a stabilized form of HIF-1α used because HIF-1α is rapidly degraded under unstressed conditions. Specific antibodies against each protein co-immunoprecipitated the other proteins, indicating the presence of YAP-TEAD1, YAP–HIF-1α, and TEAD1–HIF-1α complexes in both HEK293 cells (Figure 13, A–C) and NRVMs (Figure 13, D–F). However, when only 2 of the 3 proteins were overexpressed in HEK293 cells, the YAP–HIF-1α complex was detected but not the TEAD1–HIF-1α complex (Figure 13G), suggesting that YAP may be required for TEAD1–HIF-1α interaction.

To examine whether the interactions among YAP, TEAD1, and HIF-1α in CMs are direct or indirect, we conducted in vitro protein-protein interaction assays. When recombinant HIF-1α and TEAD1 were incubated, either individually or in combination, with glutathione S-transferase–YAP (GST-YAP) or GST-control, both HIF-1α and TEAD1 bound to GST-YAP but not to GST-control, suggesting that HIF-1α and TEAD1 can interact with YAP directly (Figure 13H). When recombinant HIF-1α and YAP were incubated, either individually or in combination, with GST-TEAD1 or GST-control, although YAP bound to GST-TEAD1 under either condition, HIF-1α only bound to GST-TEAD1 in the presence of YAP, suggesting that YAP could interact with TEAD1 directly but that HIF-1α could only interact with TEAD1 through YAP (Figure 13I). Similarly, when recombinant TEAD1 and YAP were incubated, either individually or in combination, with GST–HIF-1α or GST-control, although YAP bound to GST–HIF-1α under either condition, TEAD1 bound to GST–HIF-1α only in the presence of YAP, suggesting that YAP could interact with HIF-1α directly but that TEAD1 could only interact with HIF-1α through YAP (Figure 13J). When equal amounts of recombinant TEAD1 were incubated with GST–HIF-1α, the amount of TEAD1 that bound to GST–HIF-1α increased proportionately to the amount of recombinant YAP present (Figure 13K). Thus, YAP could interact with either TEAD1 or HIF-1α directly, but TEAD1 and HIF-1α interacted with one another through YAP. These results suggest that PO-induced upregulation of YAP and the consequent YAP-TEAD1 or YAP–HIF-1α interaction promote TEAD1–YAP–HIF-1α interaction and upregulation of GLUT1 transcription (Figure 13L).

## Discussion

The heart undergoes cardiac hypertrophy to compensate for acute hemodynamic overload by increasing wall thickness and activating cell survival mechanisms (1). We previously showed that YAP is activated by acute PO and that endogenous YAP plays an essential role in mediating compensatory hypertrophy in the heart in vivo (3). Other studies have shown that acute PO activates aerobic glycolysis, namely the Warburg effect (25, 26), and that metabolites of the glycolytic pathway can induce cardiac hypertrophy by acting as building blocks (12, 25). However, the involvement of YAP in the Warburg effect in the heart during acute stress remained unclear. Here, we show that endogenous YAP critically mediated the Warburg effect and that induction of compensatory hypertrophy by YAP was critically mediated by the Warburg effect and the metabolic intermediates of the glycolytic pathway.

We show that PO-induced activation of glycolysis and upregulation of GLUT1 were inhibited by downregulation of YAP in the mouse heart. Furthermore, the changes in glycolytic intermediates and metabolites induced by YAP overexpression in NRVMs were partially mimicked in hearts subjected to PO for 2 days. YAP was also necessary and sufficient to induce glycolysis in both neonatal and adult cultured CMs. Thus, YAP likely had a cell-autonomous stimulatory effect on glycolysis in the heart and the CMs therein. Since rescue of GLUT1 expression alleviated PO-induced cardiac dysfunction and decreased in cardiac hypertrophy in YAPch-KO mice, of the many known functions of YAP (27), activation of the glycolytic pathway through glucose uptake appeared to play a significant role in mediating YAP-induced compensatory cardiac hypertrophy.

PO-induced accumulation of some glycolytic intermediates and metabolites, but not others, was inhibited in the YAPch-KO hearts in vivo. On the other hand, downregulation of YAP almost fully suppressed PO-induced glycolysis in freshly isolated adult CMs in vitro. Therefore, PO may have induced compensatory mechanisms that maintained glycolysis in the YAPch-KO hearts in vivo. Since the glycolytic pathway is regulated by multiple mechanisms, YAP-induced transcription may not be the only mechanism that regulates this pathway’s protein levels and enzyme activities. We noted that endogenous AMP-activated protein kinase (AMPK) activity, indicated by the ratio of Ser79/Ser212 phosphorylated acetyl-CoA carboxylase 1, -2 (ACC1/-2) to total ACC1/-2, was greater in YAPch-KO hearts than in control hearts at baseline and 2 days after TAC (Supplemental Figure 1, K–P, S, and T). AMPK may stimulate glycolysis (28) and partially restore the levels of glycolytic intermediates and metabolites in vivo. However, the functional significance of AMPK activation in this context remains to be elucidated. Even in the presence of AMPK, PO-induced upregulation of l-serine, l-aspartate, and malate was significantly (*P* < 0.05) attenuated in YAPch-KO hearts 2 days after TAC, suggesting that YAP provided the auxiliary and anaplerotic pathways with substrates through glycolysis, a process for which AMPK may not compensate.

YAP-induced cardiac hypertrophy in NRVMs was significantly (*P* < 0.05) inhibited when PGK or any enzyme on the glycolytic pathway upstream of PGK was inhibited but not when PGAM or any enzyme downstream of PGAM was inhibited. Interestingly, inhibition of PGAM or any enzyme downstream of PGAM alone induced modest cardiac hypertrophy, even without overexpression of YAP, suggesting that intermediate products of the glycolytic pathway between F6P and PEP may be involved in YAP-induced cardiac hypertrophy (Figure 7C). Among the enzymes that activate the auxiliary pathways branching from the glycolytic pathway, suppression of either GFAT, which mediates activation of the hexosamine biosynthetic pathway (29), or PHGDH, which mediates activation of the serine biosynthetic pathway (17), also inhibited YAP-induced cardiac hypertrophy. Among glycolytic intermediates and metabolites, YAP overexpression induced significant (*P* < 0.05) accumulation of 3-PG, PEP, and cystathionine. Thus, we speculate that YAP activates the serine biosynthetic pathway. Consistently, suppression of PHGDH inhibited YAP-induced accumulation of H3K4me3 and GSH, whose production is known to be stimulated by the serine biosynthetic pathway (17). Thus, YAP-induced stimulation of the serine biosynthetic pathway may promote hypertrophic growth and cell survival, thereby acting in a compensatory manner in the heart during PO.

Stimulation of fatty acid oxidation (FAO) negatively affects glucose oxidation through a mechanism called the Randall effect (25). Downregulation of ACC2 and upregulation of FAO inhibit PO-induced cardiac hypertrophy by suppressing glucose-dependent anabolic mechanisms, including aspartate synthesis (25, 26). Conversely, increases in glucose oxidation through a reduction of FAO improve cardiac function in failing hearts (30). Furthermore, increasing glucose utilization through overexpression of GLUT1 in hypertrophied hearts protects against contractile failure and LV dilation after PO (31). These studies clearly suggest that the glycolytic pathway is critically involved in mediating cardiac hypertrophy, survival, and energetics, mimicking the effect of YAP upon glycolysis demonstrated in the current study.

It has been shown that inhibition of the glucose-dependent anabolic mechanism by increased FAO inhibits cardiac hypertrophy without inducing cardiac dysfunction (25, 26). Although downregulation of endogenous YAP inhibits cardiac hypertrophy, unlike stimulation of FAO via the downregulation of ACC2, YAP inhibition promotes cardiac dysfunction in response to acute PO (3). Interestingly, increased FAO and YAP haploinsufficiency differentially affected the metabolomic profile, including serine accumulation; the accumulation of serine in response to hypertrophic stimuli was inhibited by YAP haploinsufficiency but not by increased FAO (25). We speculate that increased FAO and YAP haploinsufficiency affect glycolysis at distinct steps. They may therefore have distinct effects on the levels of glycolytic intermediates and metabolites and, thus, differentially affect consequent activation of the anabolic mechanisms and cell survival mechanisms.

Our results suggest that upregulation of YAP during acute PO allows YAP to interact with HIF-1α, thereby upregulating GLUT1. Since endogenous TEAD1 also plays an essential role in mediating GLUT1 expression and upregulation of glycolysis (21), we speculate that the YAP-TEAD1 complex formed on the distal promoter or the enhancer of the *Glut1* gene is involved in the formation of the TEAD1–YAP–HIF-1α complex through chromatin looping. Further investigation is required to prove this hypothesis.

Although the current study suggests that stimulation of YAP is salutary during acute PO, we previously showed that persistent YAP activation, induced by genetic downregulation of WW45 and consequent inactivation of the Hippo kinases, during chronic PO is detrimental (7). Persistent YAP activation induces dedifferentiation of CMs, causing contractile dysfunction in the presence of PO (7). Why does the effect of YAP differ between the acute and chronic phases of PO? The detrimental effect of YAP during chronic PO is mediated by induction of oncostatin M, an inflammatory cytokine, through YAP-TEAD1–dependent mechanisms (7). However, the salutary actions of YAP during acute PO are mediated through the upregulation of GLUT1 by a TEAD1–YAP–HIF-1α–dependent mechanism. YAP may interact with different transcription factors in a time- and stress-dependent manner, depending on the availability of transcription factors. Although HIF-1α was significantly elevated in the heart 2 days after TAC, TEAD1 was not (Supplemental Figures 4 and 5). On the other hand, TEAD1 is upregulated in the heart (7), whereas HIF-1α is not during chronic PO. Thus, stabilization of HIF-1α, but not TEAD1, may allow YAP to induce salutary actions even during chronic PO by inducing compensatory cardiac hypertrophy. Chronic overexpression of GLUT1 alleviated cardiac dysfunction in a mouse model of PO (31). Clinically, treatment with CPT1 inhibitors alleviated cardiac dysfunction in patients with heart failure, presumably stimulating glucose oxidation by reducing FAO (30). Upregulation of the YAP–HIF-1α complex may serve as an alternative mechanism to upregulate GLUT1, thereby alleviating heart failure during chronic PO. However, persistent activation of HIF-1α could be maladaptive, depending on the context (32). Thus, the level and duration of HIF-1α activation may require adjustment.

Myocardial expression of connective tissue growth factor (CTGF), a well-established transcriptional target of YAP, was found to be significantly elevated in patients with diastolic heart failure compared with patients with normal cardiac function (33), suggesting that YAP may be activated by PO in the human heart. Further investigation is needed to determine whether YAP activation induces compensatory cardiac hypertrophy in patients with PO.

In summary, YAP mediated compensatory cardiac hypertrophy by stimulating the Warburg effect during acute PO in the heart (Figure 13L). YAP induced the accumulation of intermediates and metabolites of the glycolytic pathway. Although there may be some compensation for the loss of YAP function in the heart during PO, YAP appeared to be indispensable for the production of l-serine, l-aspartate, and malate in the glycolytic, auxiliary, and anaplerotic pathways through direct upregulation of GLUT1. We found that these, in turn, were involved in both anabolism and cell survival mechanisms, thereby playing an essential role in mediating compensatory cardiac hypertrophy.

## Methods

### Mouse models.

Generation of transgenic mice harboring a floxed *Yap1* allele (C57BL/6 background) and α-MHC Cre recombinase–transgenic mice (C57BL/6 background) has been reported (16). Two- to 3-month-old male *Yap^+/fl^* (control) and *Yap^+/fl^* *Cre* (YAPch-KO) mice were used in the experiments. Mice were housed in a temperature-controlled environment within a range of 21°C–23°C with a 12-hour light/12-hour dark cycle and were given free access to water and a standard diet. To collect heart samples, mice were euthanized by cervical dislocation.

### Cell line.

HEK293 cells obtained from the American Type Culture Collection (ATCC) were maintained at 37°C with 5% CO_2_ in DMEM supplemented with 10% FBS and penicillin/streptomycin. For immunoblotting samples, HEK293 cells were grown to approximately 80% confluence in plastic dishes and transiently transfected for 3 days with the indicated plasmids using polyethylenimine.

### TAC.

The method for TAC has been described previously (7). Mice were subcutaneously injected with a small volume of bupivacaine, anesthetized with pentobarbital (60–70 mg/kg, i.p.), and mechanically ventilated. Aortic constriction was performed by ligation of the transverse thoracic aorta between the innominate artery and left common carotid artery with a 27 gauge needle using a 7-0 braided polyester suture. After surgery, mice were allowed to recover in a ThermoCare unit, followed by injection with Meloxicam-SR (4 mg/kg, s.c.).

### AAV.

The recombinant AAV vectors used to generate AAV-DJ/8-GFP (AAV-control) and AAV-DJ/8-GLUT1 (AAV-GLUT1) were constructed by subcloning the cDNA of GFP or GLUT1 (human) into the pAAV-MCS expression vector (Cell Biolabs). 293AAV cells (Cell Biolabs) were cotransfected with the recombinant AAV vector, pAAV-DJ/8 vector, and helper plasmid at a 1:1:1 ratio using polyethylenimine at the AAV core facility of Rutgers University. The recombinant AAV product was purified by the iodixanol gradient/ultracentrifugation method, and the AAV fraction was concentrated using a VIVASPIN 20 concentrator (100 kDa cutoff, Sartorius). The virus titer was determined using the Cell Biolabs AAV quantitation kit (VPK-145). To administer recombinant AAVs, doses of 2 × 10^11^ vector genome per mouse were injected i.v. via the jugular vein into 2- to 3-month-old male control and YAPch-KO mice, as described previously (34). Two weeks after the injection, the mice were subjected to sham or TAC surgery.

### Echocardiography.

Mice were anesthetized with 2.5% avertin (12 μL/g body weight, i.p.). Echocardiography was performed using ultrasonography (Acuson Sequoia C256, Siemens; ref. 7) with a 13 MHz linear ultrasound transducer. For all mouse experiments and data analysis were conducted in a blinded manner.

### Histological analysis.

Heart specimens were fixed with 4% paraformaldehyde and sectioned at 6 μm thickness. CM size was evaluated using wheat germ agglutinin (WGA) staining and ImageJ software (NIH). The cell proliferation index was evaluated by immunohistochemical analysis using the antibodies described in Supplemental Table 5 and ref. 7.

### Primary cultures of NRVMs.

Primary cultures of NRVMs were prepared from approximately 100 pooled hearts of both male and female 1-day-old Hsd:WI Wistar rats (Envigo; ref. 7). A CM-rich fraction was obtained by centrifugation through a discontinuous Percoll gradient. NRVMs were cultured overnight in complete medium containing DMEM/F12 supplemented with 5% horse serum, 4 μg/mL transferrin, 0.7 ng/mL sodium selenite, 2 g/L BSA, 3 mM pyruvate, 15 mM HEPES (pH 7.1), 100 μM ascorbate, 100 mg/L ampicillin, 5 mg/L linoleic acid, and 100 μM 5-bromo-2′-deoxyuridine (MilliporeSigma). The medium was then changed to normal serum-free DMEM/F12 medium with penicillin/streptomycin. Culture dishes were coated with 0.3% gelatin. Ad transduction of NRVMs with the indicated Ads was done in serum-free DMEM/F12 medium. For siRNA transfection, NRVMs were transfected overnight with the siRNAs (2–5 nM) described in Supplemental Table 6. After changing the medium, the cells were transduced with Ad-LacZ or Ad-FLAG-YAP for 5 days.

### Ad construction.

Recombinant Ad vectors for overexpression and shRNA-mediated gene silencing were constructed, propagated, and titered as previously described (16). The pBHGloxΔE1,3Cre plasmid was cotransfected with the pDC316 shuttle vector (Microbix Biosystems) or the pDCSilencer vector (Microbix Biosystems) containing the gene of interest into HEK293 cells using Lipofectamine 2000 (Thermo Fisher Scientific).

### Primary cultures of AMVMs.

AMVMs were isolated as previously described with some modification (35). Briefly, the hearts from male 2- to 3-month-old control or YAPch-KO mice were perfused with 5 mL EDTA buffer using a 27 gauge needle from the right ventricle to arrest the heart. After the ascending aorta was clamped, digestion was achieved by sequential injection of 5 mL perfusion buffer and 20 mL perfusion buffer containing collagenase type II (2.5 mg/mL, Worthington) into the LV. Cellular dissociation was stopped by adding 5 mL perfusion buffer containing 5% FBS. CMs and non-CMs were separated by 4 sequential rounds of gravity setting with gradual addition of Seahorse XF Base medium (103335-100, Agilent Technologies) or culture M199 medium (M4530, MilliporeSigma) supplemented with 0.1% BSA, 10 mM 2,3-butanedione monoxime (BDM) (MilliporeSigma), 1% chemically defined lipid (11905031, Thermo Fisher Scientific), and penicillin/streptomycin. More than 60% rod-shaped AMVMs were observed.

### CM size analysis.

For evaluation of NRVM or AMVM hypertrophy, CMs were fixed with 4% paraformaldehyde, and CM size was evaluated using WGA-conjugated CF488 (Biotium) staining and ImageJ software (NIH).

### Seahorse real-time cell metabolic assay.

The ECAR (mpH/min) and OCR (pmol/min) in cultured CMs were determined using a Seahorse XF96 Extracellular Flux Analyzer (Agilent Technologies) according to the company’s Installation and Operation Manual and as described previously (36). NRVMs and AMVMs were plated in 96-well Seahorse assay plates at a density of 1 × 10^5^ cells/well and 5000 cells/well, respectively. One hour before beginning the measurements, the medium was replaced with XF assay medium, and the cells were incubated for 1 hour at 37°C without CO_2_. For ECAR measurements, after baseline measurements, 10 mM glucose, 1 μM oligomycin, and 50 mM 2-deoxy d-glucose (2DG) were sequentially injected into each well. For OCR measurements, the medium contained 10 mM glucose in the presence or absence of 1 mM pyruvate. After baseline measurements, 1 μM oligomycin for NRVMs or 2 μM oligomycin for AMVMs, 3 μM FCCP, and 1 μM rotenone and 1 μM antimycin A (R/A) were sequentially injected into each well. The ECAR and OCR were normalized to the protein amount.

### qPCR.

Total RNA was prepared from mouse LVs and NRVMs using TRIzol (Thermo Fisher Scientific). cDNA was generated using 1000 ng total RNA and PrimeScript RT Master Mix (Takara). qPCR was performed using TB Green Premix Ex Taq (Takara). Ribosomal protein S15 served as an internal control. The oligonucleotide primers used to carry out the qPCRs are listed in Supplemental Table 7.

### Immunoblotting and immunoprecipitation.

Cells and hearts were lysed with ice-cold lysis buffer (10 mM Tris, pH 7.5, 150 mM NaCl, 5 mM EDTA, 1% Triton X-100, 50 mM NaF, and 10% glycerol) containing protease inhibitor (MilliporeSigma) and 1 μM MG-132 (MilliporeSigma). Protein concentrations were measured using a bicinchoninic acid (BCA) protein assay kit (Thermo Fisher Scientific). For immunoprecipitation, lysates (150 μg) were incubated with antibodies (4 μg) covalently linked to Protein G-Sepharose (17-0618-01, GE Healthcare) at 4°C for 2 hours. After washing 3 times with lysis buffer, proteins were eluted with 2× SDS sample buffer and 100 mM DTT. Denatured and undenatured protein samples (10–30 μg) were analyzed by immunoblotting using the antibodies indicated in Supplemental Table 5. Band signal intensities were quantified with ImageJ. (See complete unedited blots in the supplemental material.)

### Metabolomics analysis.

The method of metabolomics analysis has been described previously (37). Briefly, gas chromatography/mass spectrometry–based (GC/MS-based) metabolomics analysis was performed using ventricular tissue samples (~15 mg) harvested from control and YAPch-KO mice 2 days after sham operation or TAC or using NRVMs (1 × 10^7^ cells/10 cm dish) transduced with Ad-LacZ or Ad-FLAG-YAP for 5 days. All GC/MS analyses were performed with an Agilent 7200 GC-QTOF and an Agilent 7693A automatic liquid sampler. Data were collected using MassHunter software (Agilent Technologies). Metabolites were identified and their peak areas were recorded using Agilent’s MassHunter Quant. Metabolite identity was established using a combination of an in-house metabolite library developed using pure purchased standards, the National Institute of Standards and Technology (NIST) library, and the Fiehn library. The values were normalized to wet tissue weight. Analysis of the metabolomics data was carried out using MetaboAnalyst 5.0 software, including the generation of a heatmap.

### ^13^C mass isotopomer analysis.

NRVMs (1 × 10^7^ cells/10 cm dish) were transduced with Ad-LacZ or Ad-FLAG-YAP for 5 days in 10 mL serum-free DMEM/F12 medium. The cells were then processed as previously reported with some modification (38). The medium was replaced with serum-free DMEM containing 3.15 g/L uniformly labeled [U-^13^C] d-glucose (Cambridge Isotope Laboratories). After 24 hours of incubation, the cells were washed with 5 mL ice cold PBS twice and extracted with 1 mL 80% methanol. U-^13^C-glucose stable isotope tracing was performed on an Agilent Technologies system consisting of an Infinity Lab UPLC coupled with a 6545 Q-TOF mass spectrometer by Creative Proteomics. Agilent Profinder, version 8, was used for data analysis. Data were calculated as corrected abundances (peak areas) for each metabolite.

### Intracellular GSH assay.

NRVMs (1 × 10^6^ cells/well, 6-well dish) were transduced with Ad-LacZ or Ad-FLAG-YAP in the presence or absence of 30 μM CBR-5884 (MilliporeSigma), a selective inhibitor of PHGDH, for 5 days in serum-free DMEM/F12 medium. Intracellular GSH levels were measured with a GSH assay kit (BioAssay Systems) according to the manufacturer’s instructions. The absorbance at 412 nm of the standard and samples were read with a spectrophotometer at 0 minutes and 10 minutes, and GSH levels were calculated.

### Extracellular glucose and lactate assays.

AMVMs (15,000 cells/well, 96-well dish) were transduced with Ad-LacZ or Ad-FLAG-YAP for 48 hours in 200 μL M199 culture medium. Glucose consumption and lactate production were measured using the Glucose Assay Kit-WST (Dojindo Molecular Technologies) and the Lactate Assay Kit-WST (Dojindo Molecular Technologies), respectively, using each culture medium according to the manufacturer’s instructions. The absorbance at 450 nm was measured using a microplate reader. Glucose consumption after 48 hours of culturing was calculated by subtracting the glucose concentration in medium with CMs from that in the medium without CMs. The lactate concentration in medium with CMs after 48 hours of culturing was subtracted from that in the medium without CMs.

### Luciferase reporter gene assay.

Luciferase activity was measured with a luciferase assay system (Promega). After seeding NRVMs (50,000 cells/well) on a 12-well plate coated with 0.3% gelatin, reporter and the indicated expression plasmids were cotransfected using Lipofectamine 2000. The amounts of expression vectors and siRNAs transfected into the cells per well were as follows: 0.1 μg pFLAG-YAP (Addgene), 0.1 μg mock, 0.5 μg mouse *Glut1* promoter (from –1 to –1000 bps) luciferase reporter plasmids, and 5 nM siRNAs. Cells were lysed with 100 μL reporter lysis buffer 2 days after transfection. Luminescence was normalized to the protein content.

### ChIP assays.

The method for ChIP assays using chromatin from NRVMs and mouse hearts has been described previously (7). Briefly, NRVMs (1 × 10^7^ cells/10 cm dish) were transduced with Ad-LacZ or Ad-FLAG-YAP in 10 mL serum-free DMEM/F12 medium. After 2 days of transduction, the cells were crosslinked with 1% formalin for 10 minutes at room temperature. Hearts from control and YAPch-KO mice 2 days after sham operation or TAC were minced into 1 mm blocks and crosslinked with 1% formalin for 5 minutes with rotation. The nuclei were isolated and lysed, and sheared chromatin was isolated after sonication. Immunoprecipitation reactions with chromatin extractions and the antibodies described in Supplemental Table 5 were carried out overnight. Eluted chromatin fragments were purified using a PCR purification kit (QIAGEN). The collected chromatin fragments were validated by qPCR with the primers listed in Supplemental Table 7. Some ChIP assays using chromatin from mouse hearts were performed using the ChIP-IT qPCR Analysis kit (53029, Active Motif) according to the manufacturer’s protocol. Hearts were isolated from 12- to 13-week-old C57BL/6 mice 2 days after sham operation (*n =* 3) or TAC (*n =* 3). The TAC and sham group hearts were pooled and subjected to ChIP assays using the following antibodies: anti-YAP antibody (Y1200-01D, US Biological), anti-TEAD1 antibody (610923, BD Biosciences), and anti–Hif-1α antibody (36169, CST). The primers used to carry out the qPCR are listed in Supplemental Table 7.

### ChIP-Seq.

Hearts were isolated from 12 to 13-week-old C57BL/6 mice 4 days after TAC (*n =* 3) or sham operation (*n =* 3). The TAC and sham group hearts were pooled and subjected to YAP ChIP-Seq (Active Motif). Immunoprecipitation was performed using anti-YAP antibody (Y1200-01D, US Biological), followed by high-throughput Illumina sequencing using an Illumina Genome Analyzer 2. Sequencing of input DNA taken before immunoprecipitation served as a control for normalization and elimination of background noise.

### Recombinant proteins.

The bacterial expression vectors for GST-fused YAP, TEAD1, and HIF-1α were generated by subcloning the cDNA of YAP (human), TEAD1 (human), and HIF-1α (human) into the pCold-GST-vector. NEB 10-beta Competent *E*. *coli* (New England BioLabs) were transformed with pCold-GST plasmids. Protein expression was induced by addition of 1 mM isopropyl β-d-1-thiogalactopyranoside (MilliporeSigma). After overnight culturing at 15°C, the *E*. *coli* were lysed in lysis buffer (1% Triton X-100 and 1 mM DTT in PBS) with sonication. The lysate was incubated with 0.25 mL GSH–sepharose 4B (GE Healthcare) for 2 hours at 4°C. The sepharose was washed 3 times with 10 mL lysis buffer and then suspended with 1 mL cleavage buffer (20 mM Tris, pH 7.0, 150 mM NaCl, 1 mM DTT) with or without 10 U/mL PreScission Protease (GE Healthcare) overnight.

### In vitro binding assays.

Recombinant GST-fused proteins in a slurry of GST Sepharose 4B (GE Healthcare) were incubated with the indicated recombinant proteins in binding buffer (10 mM HEPES, pH 7.9, 2.5 mM MgCl_2_, 50 mM KCl, 150 mM NaCl, 5% glycerol, 1 mM DTT, and 0.1% NP-40) with rotation for 2 hours at 4°C, followed by pulldown with GST-sepharose. After washing 3 times with the binding buffer, proteins were eluted with 2× SDS sample buffer and 100 mM DTT, followed by SDS-PAGE and immunoblotting.

### Statistics.

All data are expressed as the mean ± SEM. Statistical analyses were carried out using a 2-tailed, unpaired Student’s *t* test for 2 groups or 1-way ANOVA followed by Tukey’s or Dunnett’s test for multiple comparisons. A *P* value of less than 0.05 was considered statistically significant. All statistical analyses were performed using GraphPad Prism 8 (GraphPad Software).

### Study approval.

All experiments involving animals were approved by the Rutgers New Jersey Medical School’s IACUC (PROTO9999000934 and PROTO201900140) and were performed in accordance with NIH guidelines.

## Author contributions

JS conceived the ideas. TK and JS designed the experiments. TK, SO, RM, and TN performed the experiments. PZ and ZY conducted TAC and hemodynamic measurements. YN and WM conducted echocardiographic analyses. JSW conducted metabolomics analysis. MA conducted ChIP-Seq analysis. TK analyzed the data and conducted biostatistics analysis. TK and JS wrote the manuscript, and the authors reviewed and approved the manuscript for publication.

## Supplementary Material

Supplemental data

## Figures and Tables

**Table 1:**
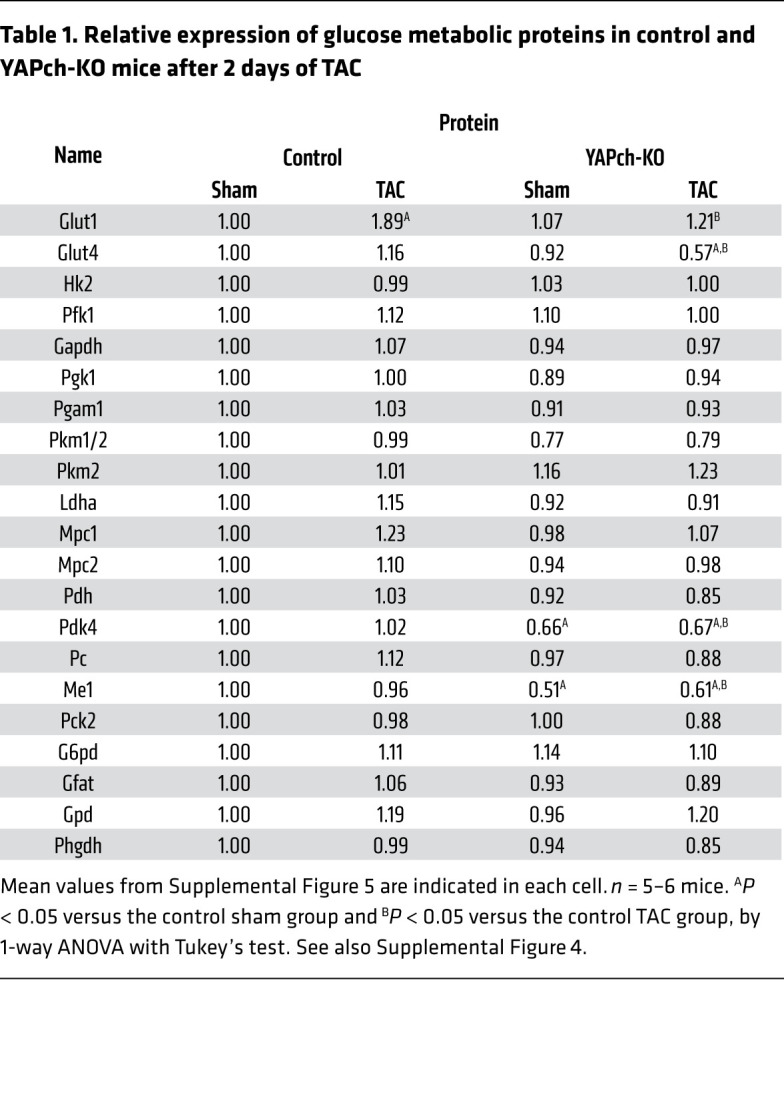
Relative expression of glucose metabolic proteins in control and YAPch-KO mice after 2 days of TAC

**Table 2:**
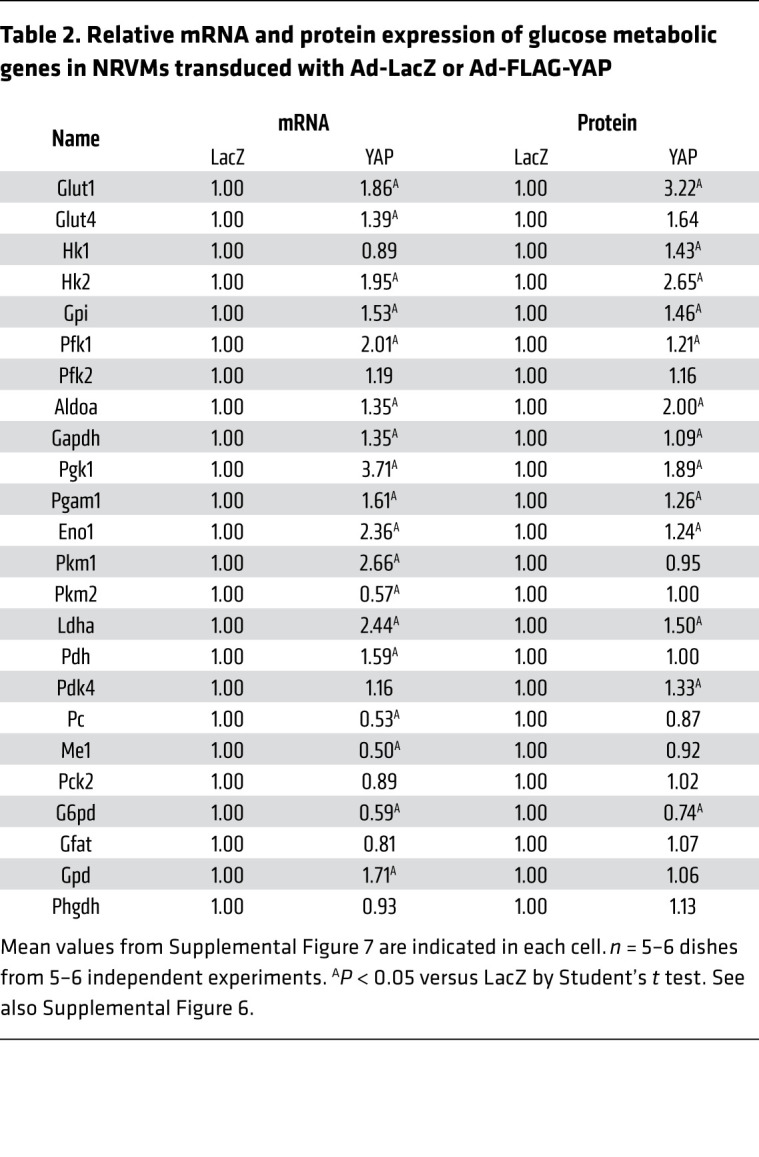
Relative mRNA and protein expression of glucose metabolic genes in NRVMs transduced with Ad-LacZ or Ad-FLAG-YAP

## Version 1 02/08/2022

In-Press Preview

## Version 2 03/15/2022

Electronic publication
