# Supplementary material for: YAP mediates compensatory cardiac hypertrophy through aerobic glycolysis in response to pressure overload
Source: J Clin Invest. 2022 Mar 15;132(6):e150595. doi: 10.1172/JCI150595 (PMC8920343; doi:10.1172/JCI150595)

Supplemental Figure 1

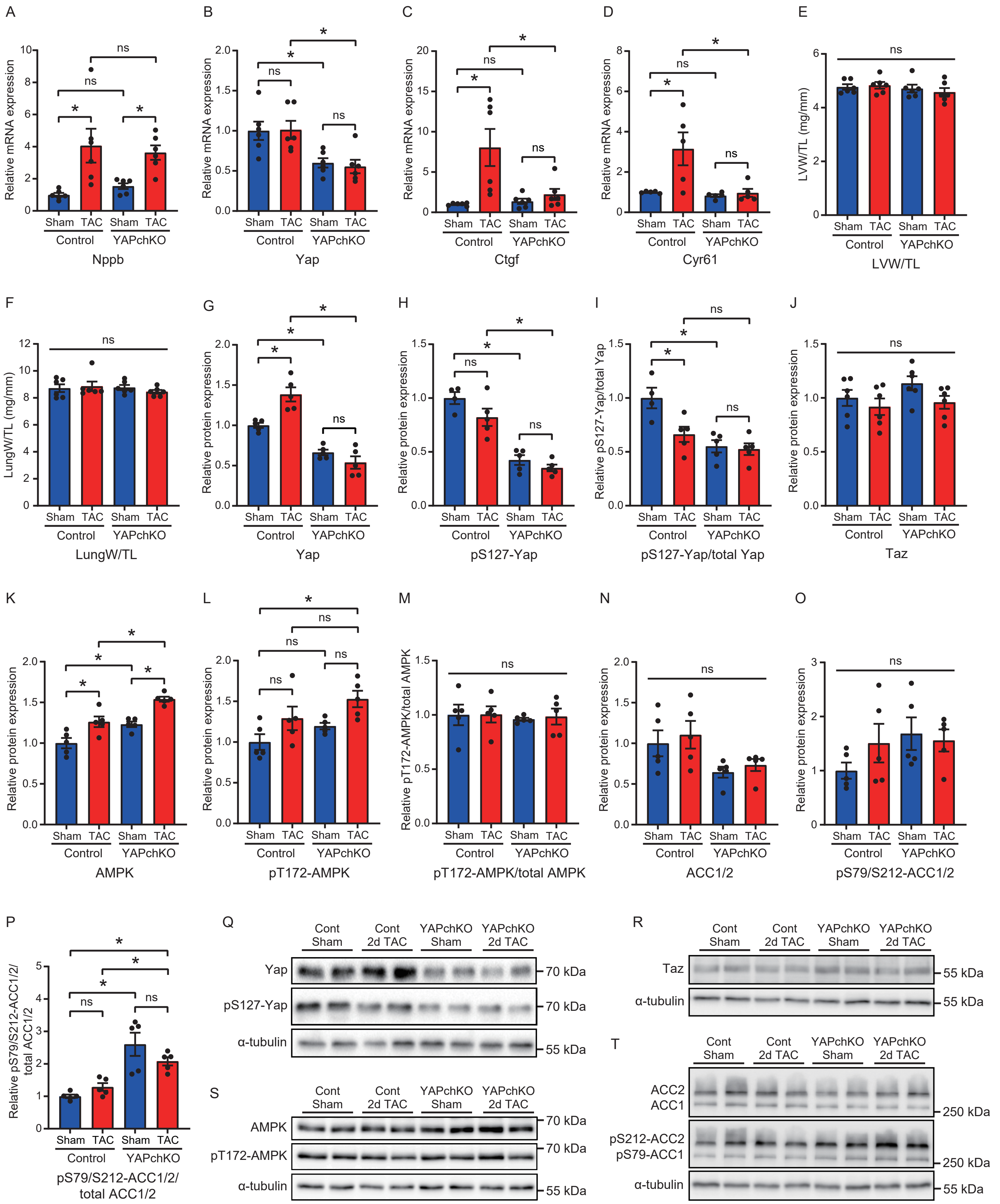

Supplemental Figure 1. Cardiac characterization of control and YAPchKO mice after 2 days of TAC. (**A**–**D**) Relative mRNA levels in the left ventricles. n = 5–6 mice. (**E**) LVW/TL. n = 6 mice. (**F**) LungW/TL. n = 6 mice. (**G**–**T**) Relative protein levels in the left ventricles (**G**–**P**) and representative immunoblots (**Q**–**T**). α-tubulin and YAP (**Q**), α-tubulin (**R**), α-tubulin and AMPK (**S**), and α-tubulin and ACC1/2 (**T**) blots, serving as loading controls, were run in parallel and contemporaneously with other blots. n = 5–6 mice. *$p < 0.05$ by 1-way ANOVA with Tukey's test. Error bars represent SEM.

Supplemental Figure 2

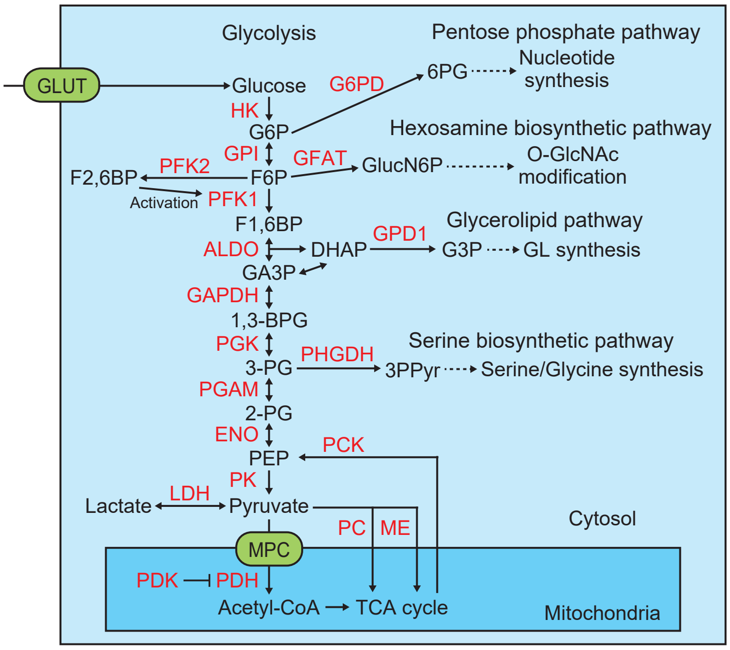

Supplemental Figure 2. Schematic representation of glycolysis and its auxiliary pathways.

Supplemental Figure 3

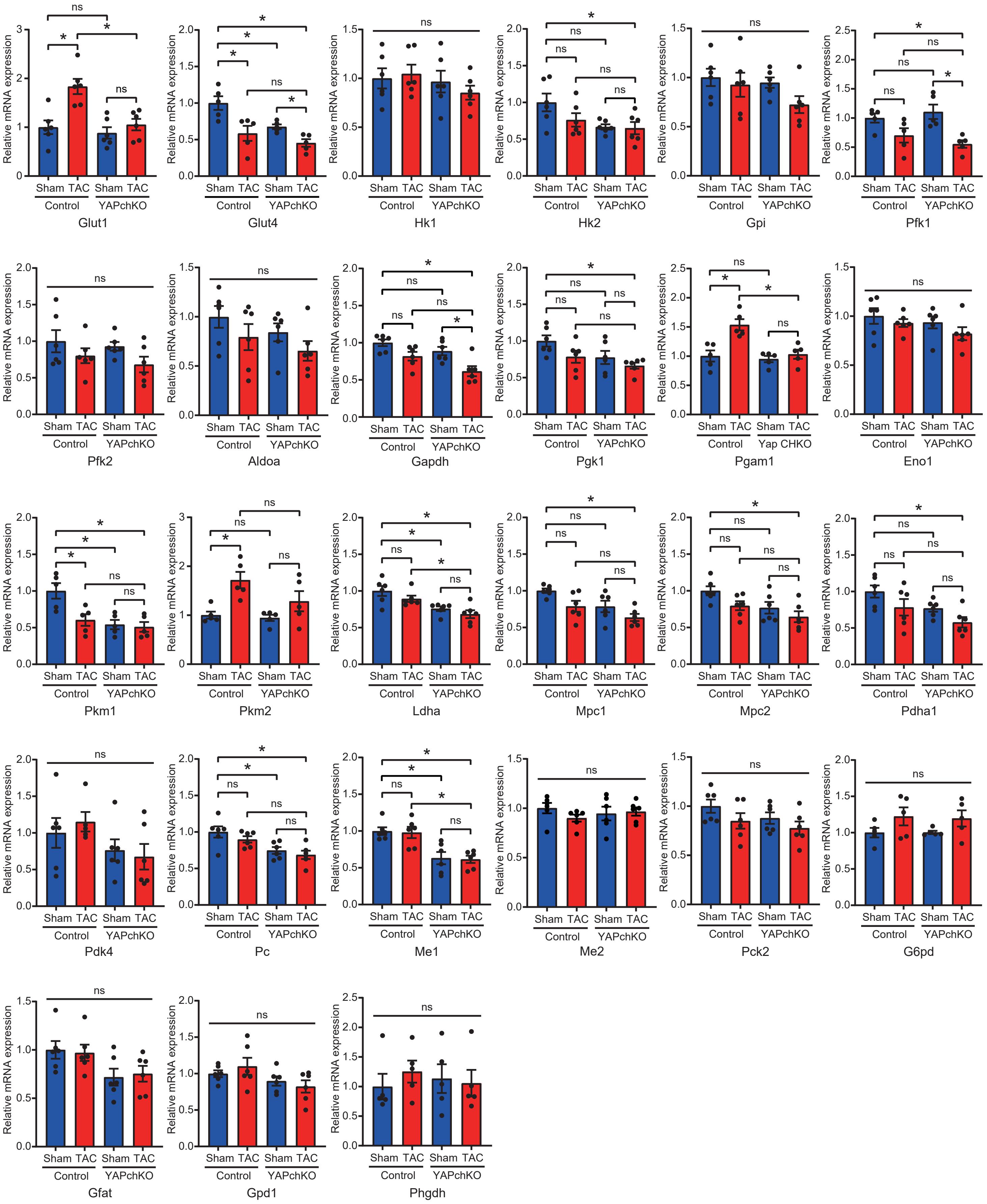

Supplemental Figure 3. Altered levels of glucose metabolic gene mRNAs in control and YAPchKO mice after 2 days of TAC. Relative mRNA levels in the left ventricles were evaluated by qPCR. n = 5–6 mice. *$p < 0.05$ by 1-way ANOVA with Tukey's test. Error bars represent SEM. See also Supplemental Table 1.

Supplemental Figure 4

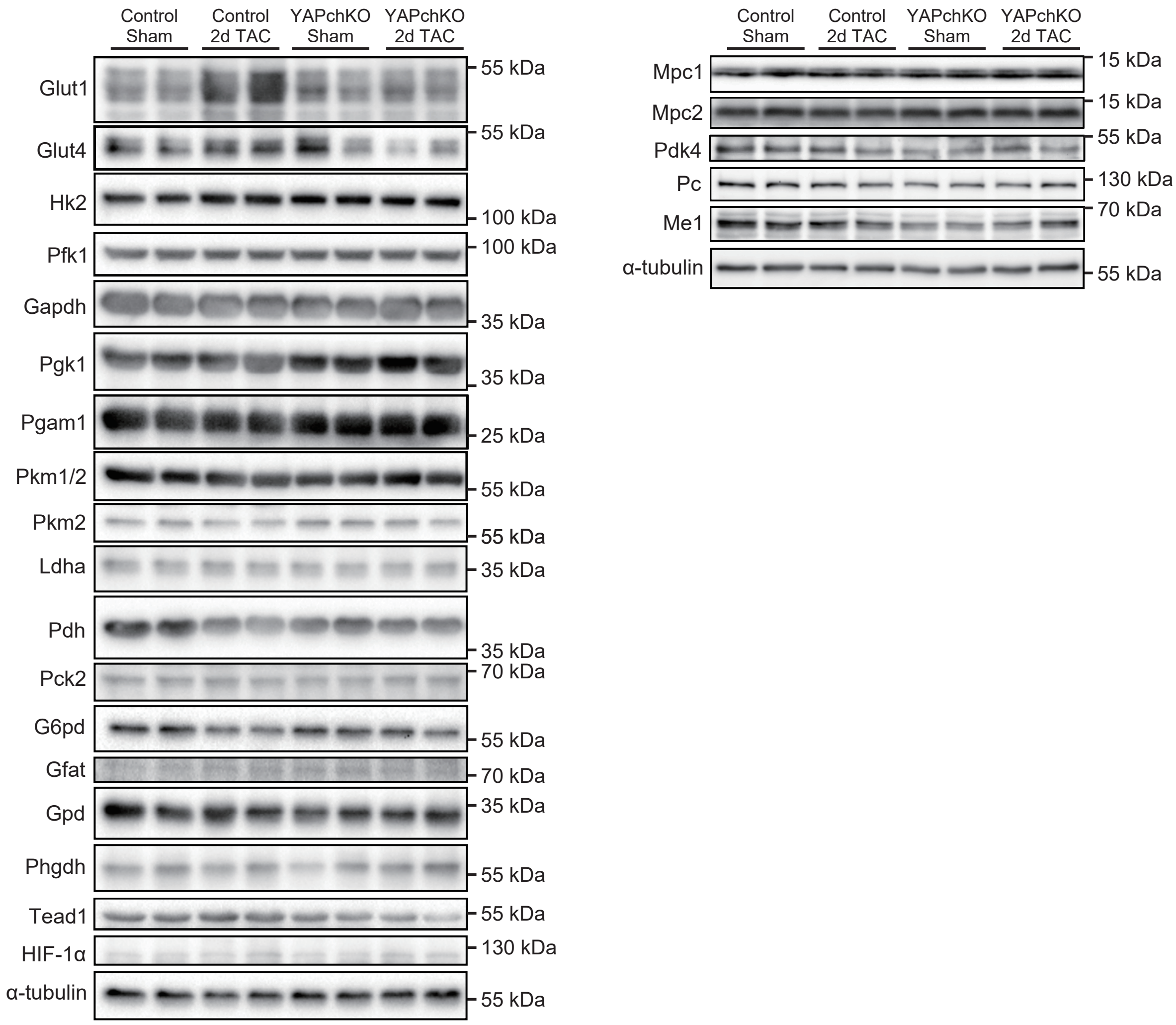

Supplemental Figure 4. Representative immunoblots of glucose metabolic proteins in the left ventricles from control and YAPchKO mice subjected to either sham operation or 2 days of TAC. α-tubulin blots, serving as loading controls, were run in parallel and contemporaneously with other blots. Relative protein expression levels of glucose metabolic proteins are shown in Supplemental Figure 5. See also Table 1.

Supplemental Figure 5

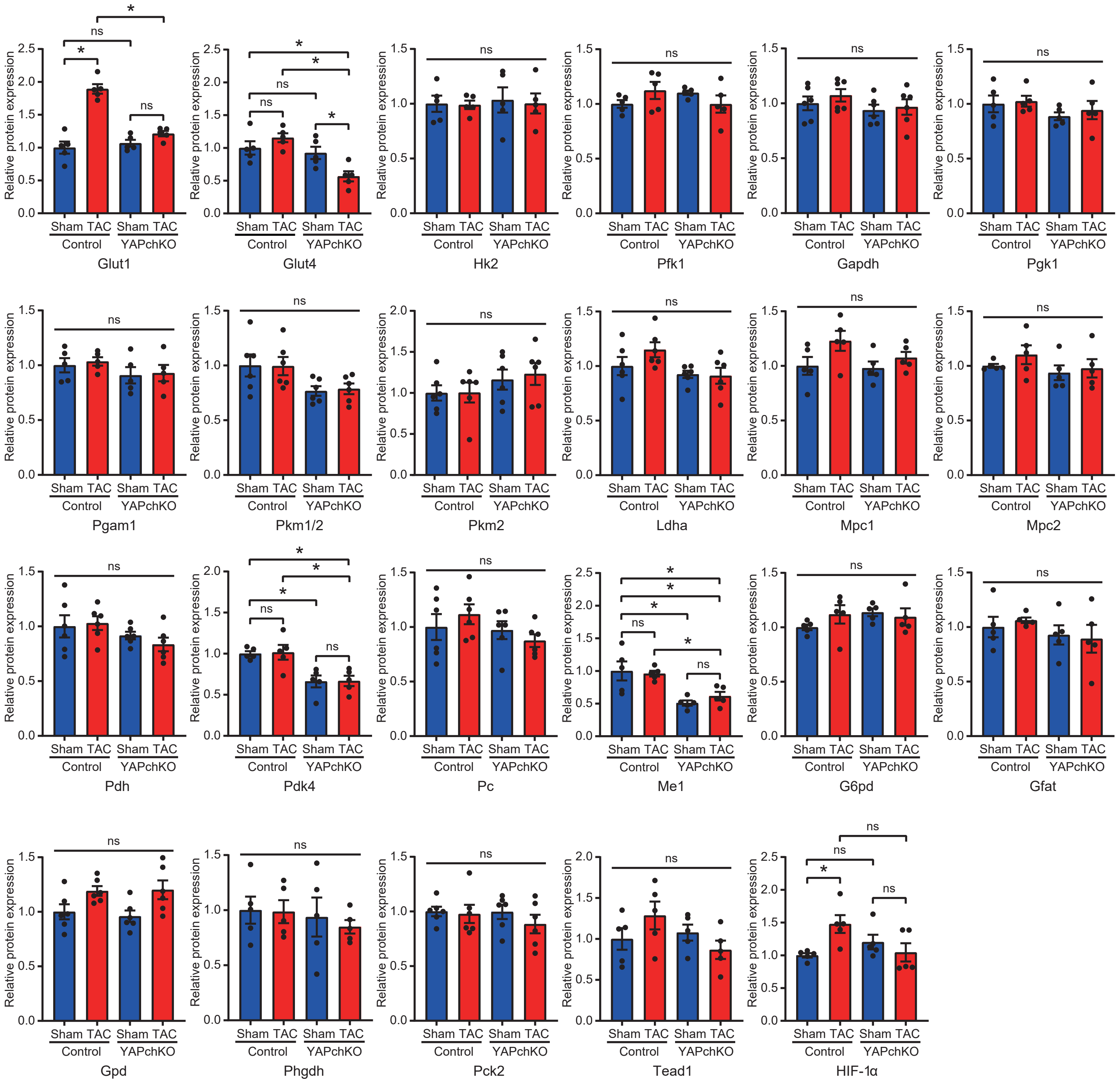

Supplemental Figure 5. Altered levels of glucose metabolic proteins in control and YAPchKO mice after 2 days of TAC. Relative protein expression levels in the left ventricles were evaluated by immunoblotting. n = 5–6 mice. *$p < 0.05$ by 1-way ANOVA with Tukey's test. Error bars represent SEM. See also Supplemental Figure 4.

Supplemental Figure 6

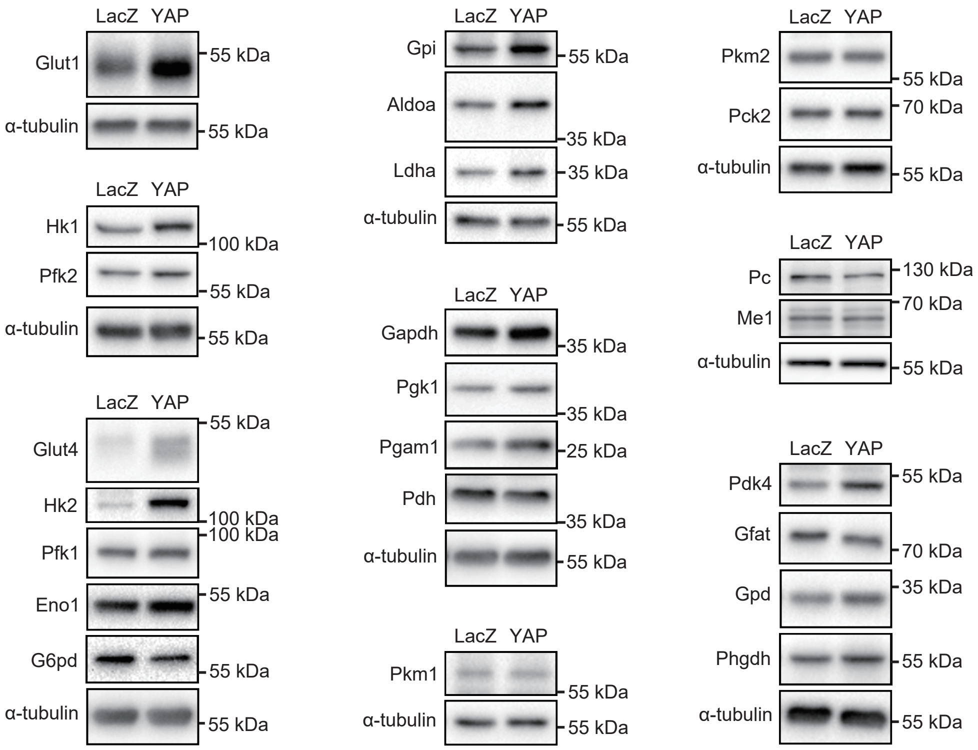

Supplemental Figure 6. Representative immunoblots of glucose metabolic proteins in NRVMs transduced with Ad-LacZ or Ad-FLAG-YAP. α-tubulin blots, serving as loading controls, were run in parallel and contemporaneously with other blots. Relative protein expression levels of glucose metabolic proteins are shown in Supplemental Figure 7B. See also Table 2.

Supplemental Figure 7

A

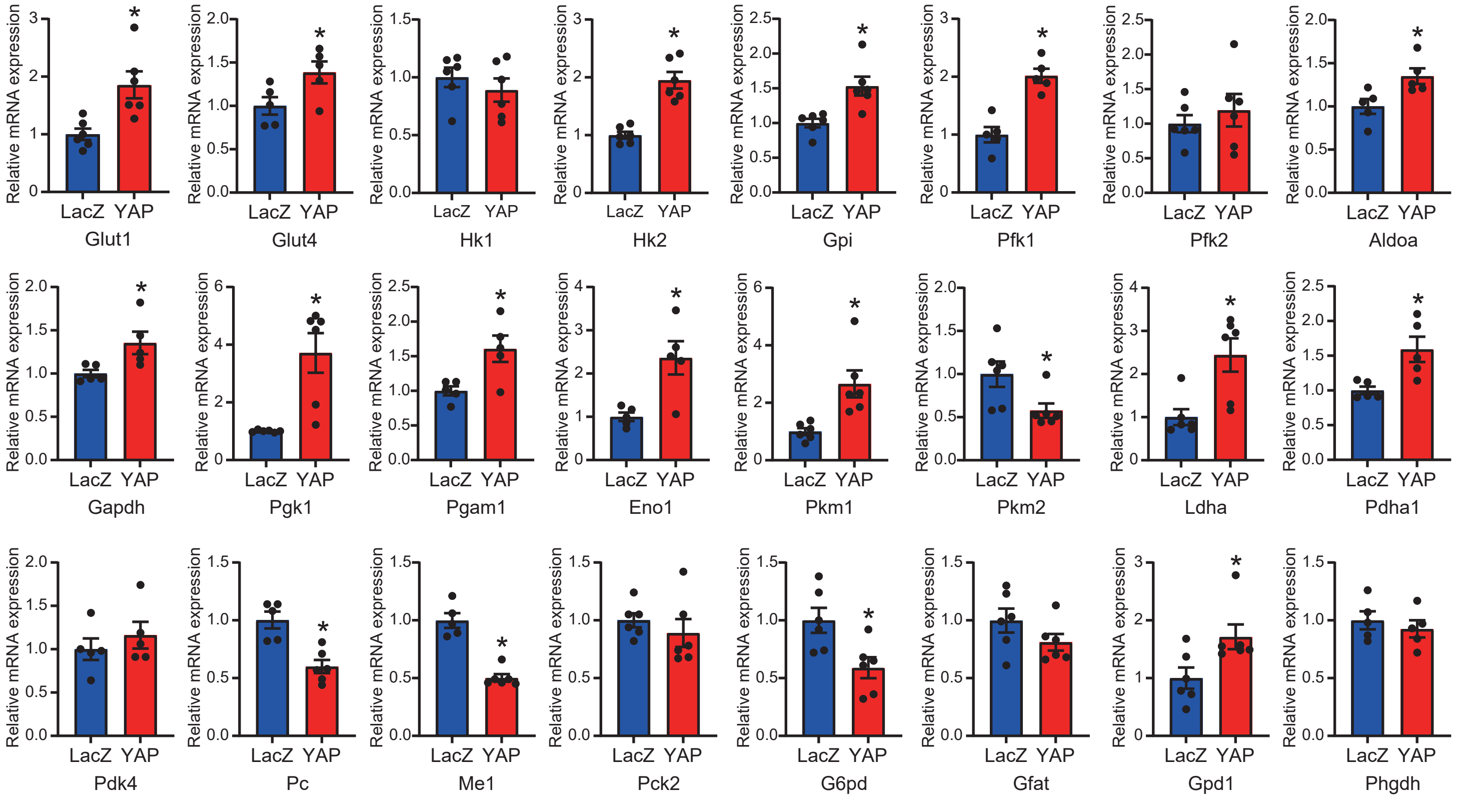

B

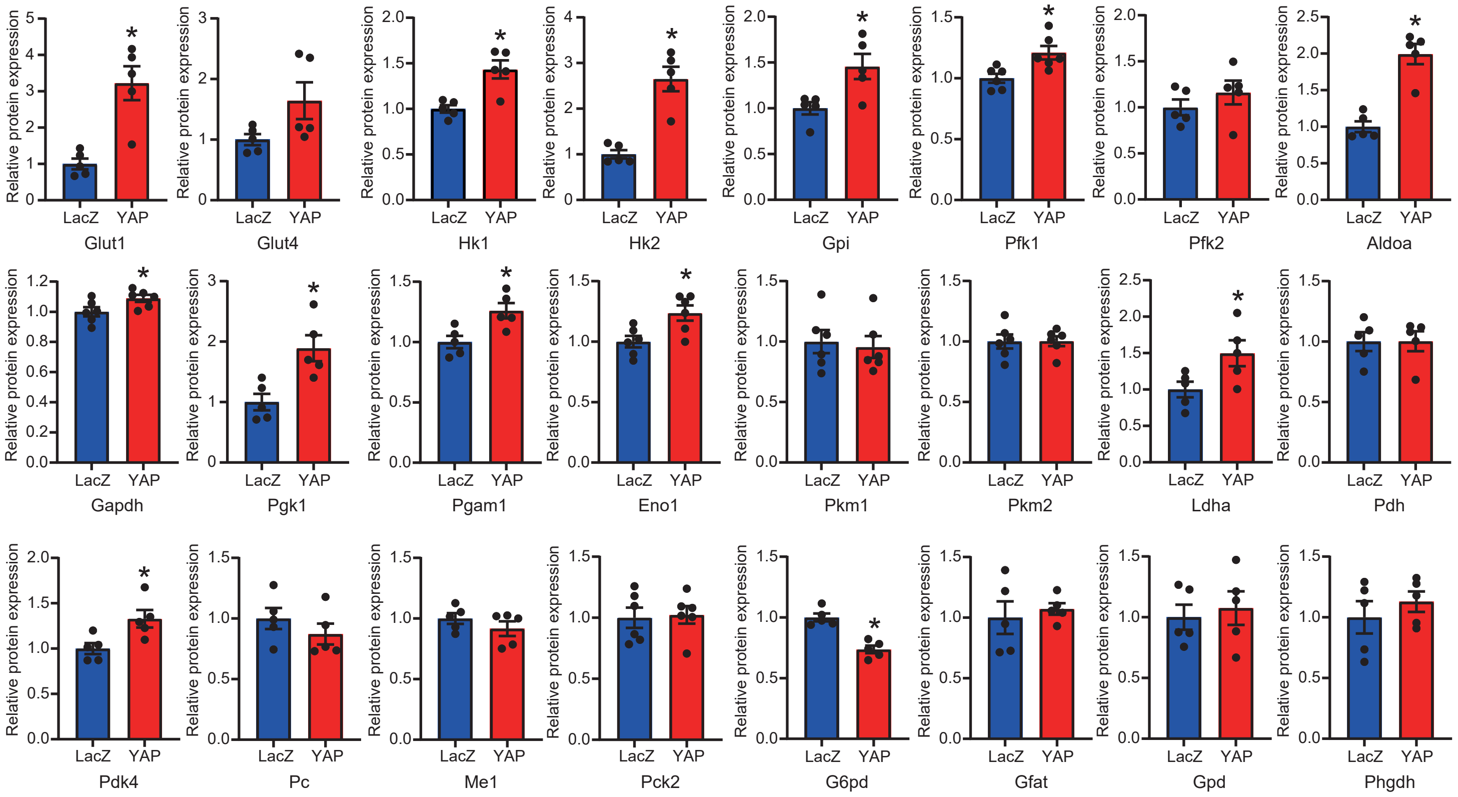

Supplemental Figure 7. Altered mRNA and protein levels of glucose metabolic factors in NRVMs overexpressing YAP. NRVMs were transduced with Ad-LacZ or Ad-FLAG-YAP for 6 days in serum-free DMEM/F12 medium. (**A**) Relative mRNA expression levels. n = 5–6 dishes from 5–6 independent experiments. (**B**) Relative protein expression levels. n = 5–6 dishes from 5–6 independent experiments. $^*p < 0.05$ by Student's *t* test. Error bars represent SEM. See also Supplemental Figure 6.

Supplemental Figure 8

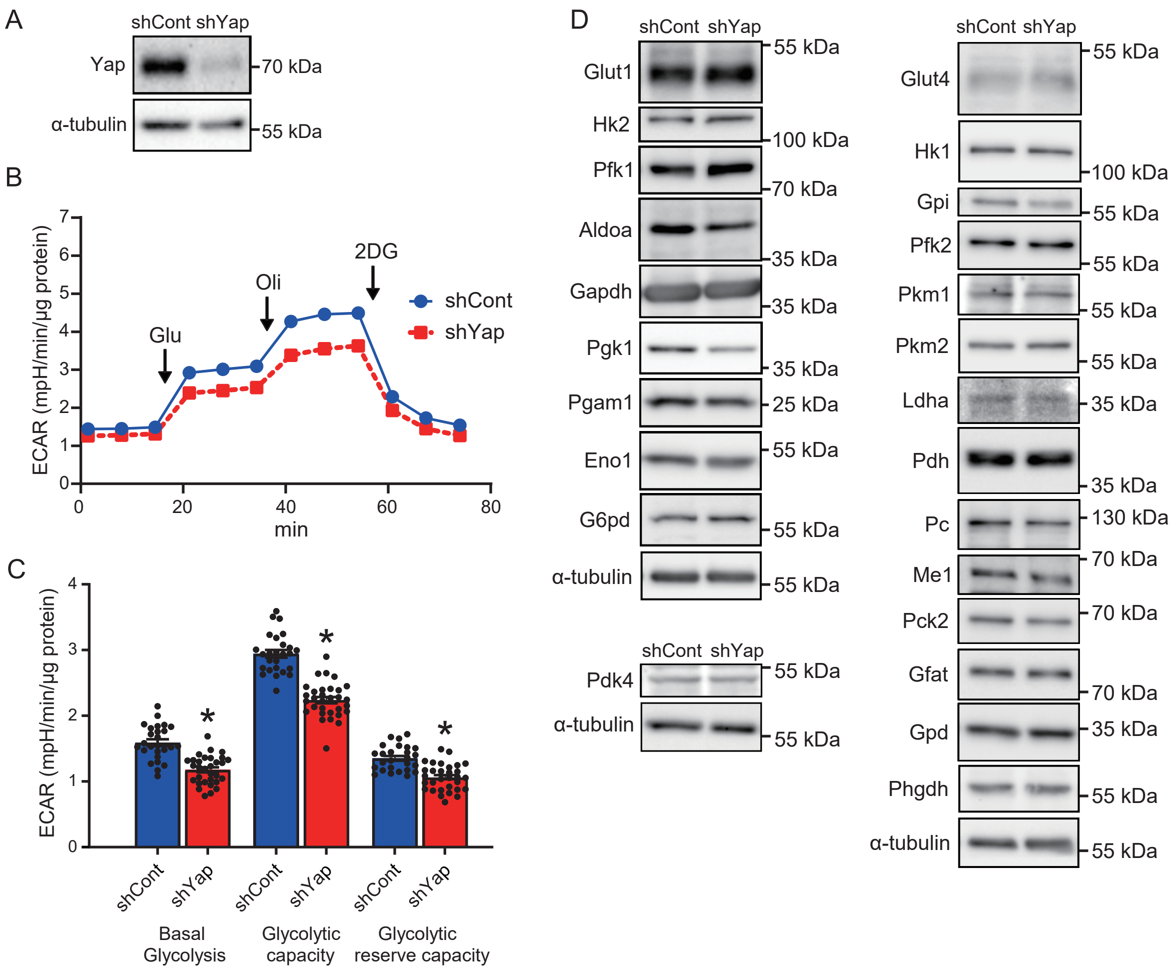

Supplemental Figure 8. Knockdown of YAP decreased glycolytic function in NRVMs. (**A**–**C**) YAP was downregulated in NRVMs transduced with Ad-shYap for 6 days compared to in those transduced with Ad-shControl (**A**). Data are representative of 3 independent blots. Measurement of ECAR (**B**) and the summary of glycolytic function (**C**). n = 26–31 wells from 3 independent experiments. *p < 0.05 by Student's *t* test. Error bars represent SEM. (**D**) Representative immunoblots of NRVMs transduced with Ad-shControl or Ad-shYap are shown. α-tubulin blots, serving as loading controls, were run in parallel and contemporaneously with other blots (**A** and **D**). Relative protein expression levels of glucose metabolic proteins are shown in Supplemental Table 3.

Supplemental Figure 9

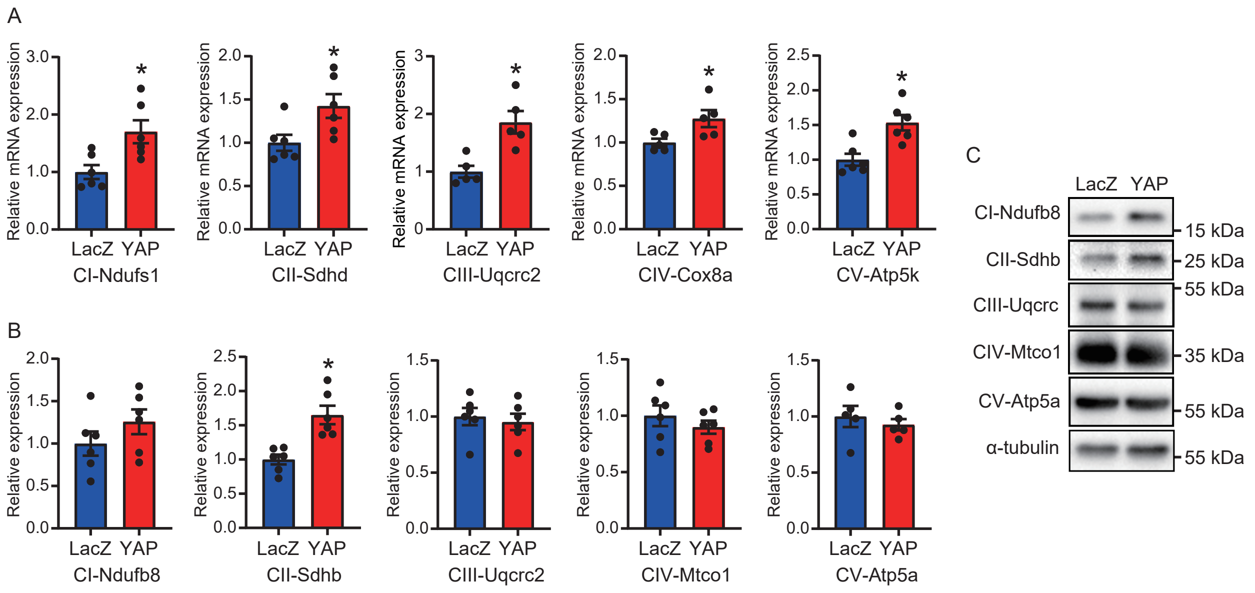

Supplemental Figure 9. Altered mRNA and protein levels of mitochondrial oxidative phosphorylation complex components in NRVMs overexpressing YAP. (**A**–**C**) Relative mRNA and protein expression levels of oxidative phosphorylation complex components were evaluated by qPCR (n = 5–6 dishes from 5–6 independent experiments) (**A**) and immunoblotting (n = 5–6 dishes from 5–6 independent experiments) (**B** and **C**), respectively. Representative immunoblots are shown (**C**). An α-tubulin blot, serving as a loading control, was run in parallel and contemporaneously with other blots. *$p < 0.05$ by Student's *t* test. Error bars represent SEM.

Supplemental Figure 10

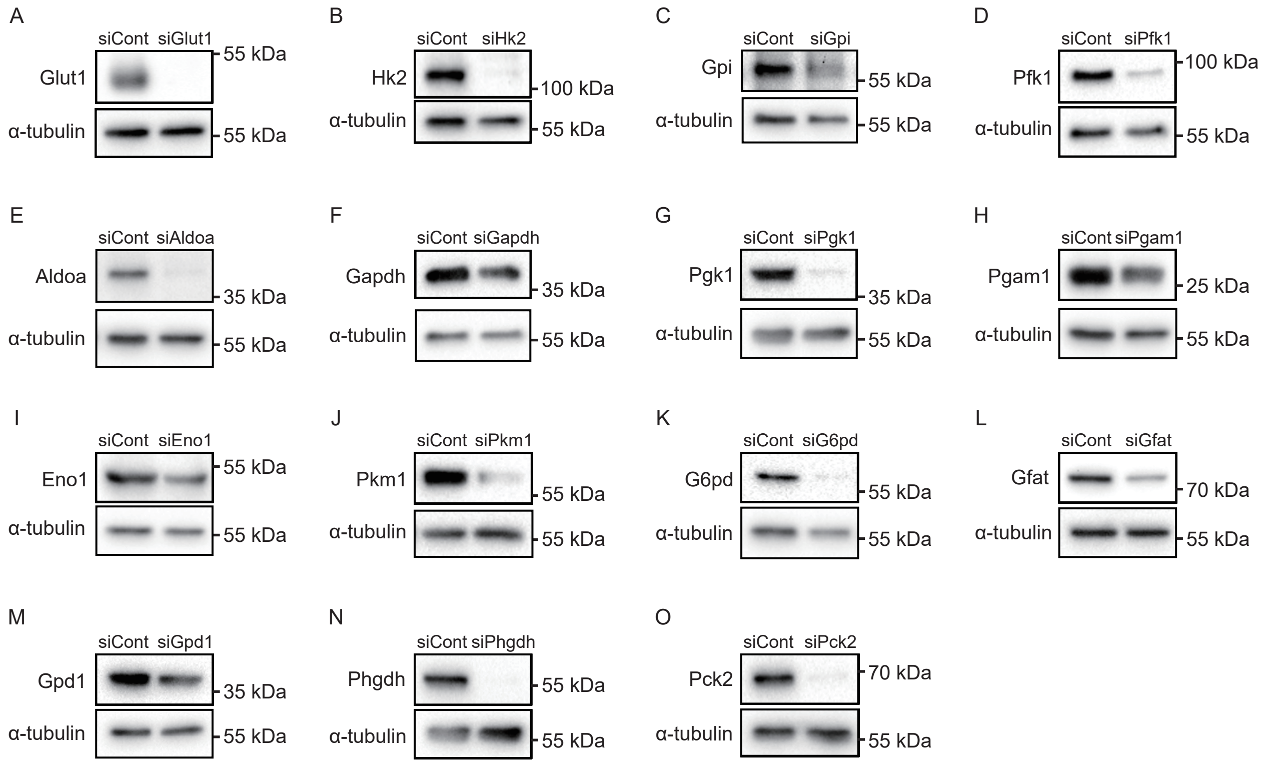

Supplemental Figure 10. Confirmation of knockdown of glucose metabolic genes in NRVMs. (**A**–**O**) Knockdown of glucose metabolic genes in NRVMs transfected with indicated siRNAs was confirmed by immunoblotting. Data are representative of 3 independent blots. α-tubulin blots, serving as loading controls, were run in parallel and contemporaneously with other blots (**A**, **G**, **J**, **K**, **N**, and **O**). See also Figure 7.

Supplemental Figure 11

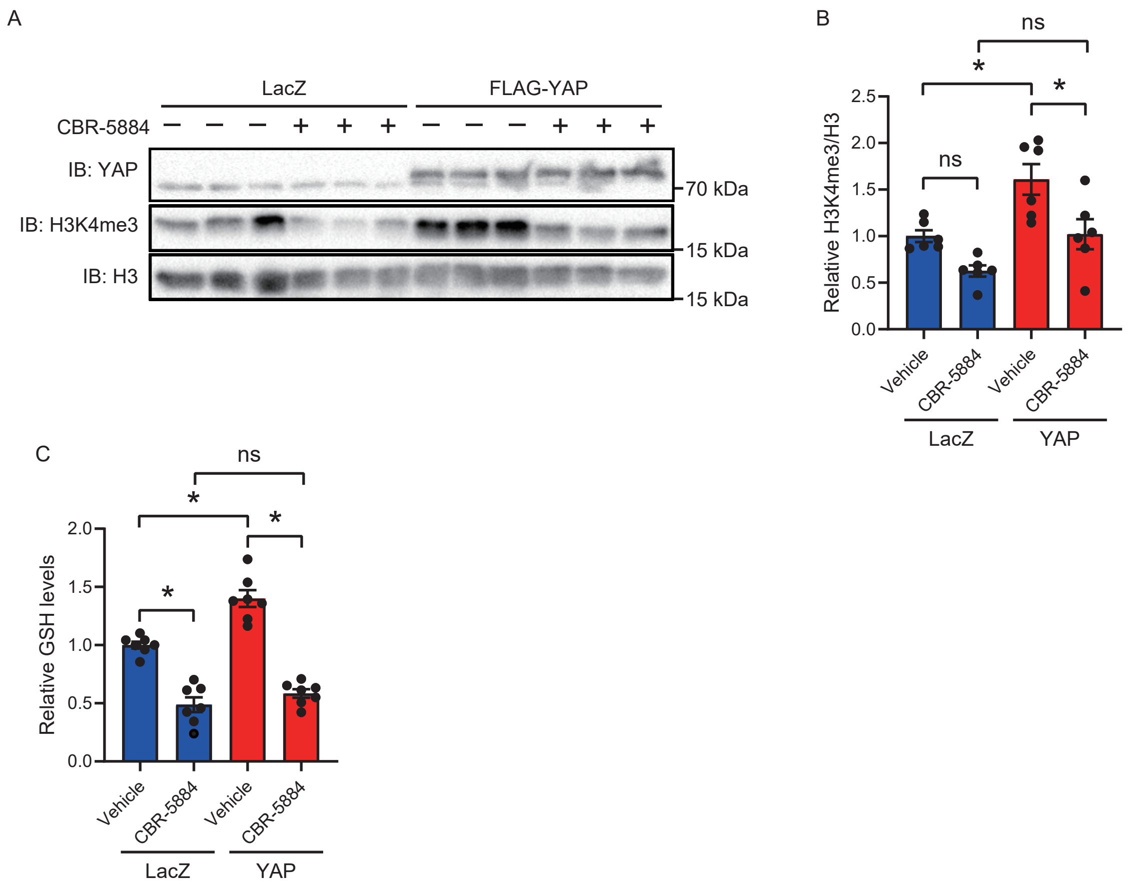

Supplemental Figure 11. Overexpressed YAP upregulated H3K4me3 and GSH. (**A**–**C**) NRVMs were transduced with Ad-LacZ or Ad-FLAG-YAP in the presence or absence of 30 μM CBR-5884, a selective PHGDH inhibitor. Representative immunoblots (**A**) and the summary of quantification (**B**) are shown. A H3 blot, serving as a loading control, was run in parallel and contemporaneously with other blots. n = 6 wells from 6 independent experiments. n = 6 wells from 6 independent experiments. Relative GSH levels are shown (**C**). n = 7 wells from 7 independent experiments. *$p < 0.05$ by 1-way ANOVA with Tukey's test. Error bars represent SEM.

Supplemental Table 1. Relative mRNA expression of glucose metabolic genes in control and YAPchKO mice after 2 days of TAC

Name	mRNA			
	Control		YAPchKO	
	Sham	TAC	Sham	TAC
Glut1	1.00	1.84[A]	0.89	1.05[B]
Glut4	1.00	0.67[A]	0.66[A]	0.50[A]
Hk1	1.00	1.05	0.97	0.85
Hk2	1.00	0.76	0.67	0.65[A]
Gpi	1.00	0.93	0.95	0.73
Pfk1	1.00	0.70	1.11	0.55[A]
Pfk2	1.00	0.80	0.93	0.68
Aldoa	1.00	0.79	0.84	0.65
Gapdh	1.00	0.82	0.89	0.62[A]
Pgk1	1.00	0.79	0.77	0.66[A]
Pgam1	1.00	1.54[A]	0.95	1.03[B]
Eno1	1.00	0.93	0.94	0.82
Pkm1	1.00	0.60[A]	0.54[A]	0.51[A]
Pkm2	1.00	1.72[A]	0.95	1.29
Ldha	1.00	0.89	0.76[A]	0.68[AB]
Mpc1	1.00	0.79	0.79	0.64[A]
Mpc2	1.00	0.79	0.77	0.65[A]
Pdh	1.00	0.80	0.74	0.55[A]
Pdk4	1.00	1.39	0.76	0.68
Pc	1.00	0.90	0.74[A]	0.69[A]
Me1	1.00	0.98	0.63[A]	0.61[AB]
Me2	1.00	0.90	0.94	0.97
Pck2	1.00	0.85	0.88	0.78
G6pd	1.00	1.22	1.00	1.20
Gfat	1.00	0.97	0.72	0.75
Gpd	1.00	1.10	0.90	0.82
Phgdh	1.00	1.25	1.14	1.06

Mean values from Supplemental Figure 3 are indicated in each cell. n = 5–6 mice. [A]$p < 0.05$ versus the control sham group and [B]$p < 0.05$ versus the control TAC group by 1-way ANOVA with Tukey's test.

Supplemental Table 2. Metabolomics analysis of energy metabolism pathways after sham or 2 days TAC in control and YAPchKO mice

Pathway	Subpathway	Metabolite	Fold change				Sham vs TAC		WT vs YAPchKO	
			WT Sham (n = 4)	WT TAC (n = 5)	YAPchKO Sham (n = 5)	YAPchKO TAC (n = 5)	WT *P* value[A]	YAPchKO *P* value[A]	Sham *P* value[A]	TAC *P* value[A]
Sugar		D-Fructose	1.000	1.178	1.585	1.004	0.8189	0.0390	0.0520	0.8032
		D-Mannose	1.000	1.222	1.166	1.013	0.8503	0.1903	0.0468	0.9985
		D-Galactose	1.000	1.344	1.469	1.550	0.3506	0.9727	0.1340	0.7028
		D-Glucose	1.000	0.872	2.045	1.074	0.9425	0.0021	0.0019	0.7873
Glucose metabolism intermediates	Glycolysis	Glucose 6-phosphate	1.000	0.728	1.396	0.971	0.7909	0.4409	0.5470	0.8160
		Fructose 6-phosphate	1.000	0.785	1.300	0.819	0.8339	0.2328	0.6513	0.9989
		Glycerol 3-phosphate	1.000	1.024	1.188	1.364	0.9990	0.7117	0.7069	0.2073
		Glyceraldehyde 3-phosphate	1.000	0.562	0.576	0.842	0.9963	0.6632	0.5749	0.9785
		Phosphoenolpyruvate (PEP)	1.000	1.173	1.153	1.411	0.3212	0.0583	0.4225	0.0869
		Pyruvic acid	1.000	1.293	1.401	1.209	0.2123	0.5014	0.0568	0.9227
		L-Lactic acid	1.000	1.556	1.496	1.368	0.1384	0.9412	0.2088	0.8398
	Pentose phosphate pathway	Ribitol	1.000	0.981	1.328	1.301	0.9988	0.9957	0.0872	0.0749
		Myoinositol	1.000	0.863	1.328	0.941	0.8835	0.1711	0.3349	0.9707
		Inositol phosphate	1.000	0.827	0.919	0.846	0.1613	0.7490	0.7305	0.9940
		Sedoheptulose 7-phosphate	1.000	0.708	1.675	1.271	0.8602	0.1966	0.0239	0.9754
TCA cycle		Citric acid	1.000	1.130	1.308	1.225	0.9700	0.9902	0.7253	0.9858
		cis-Aconitic acid	1.000	1.421	1.427	1.254	0.1702	0.7751	0.1612	0.7934
		Isocitric acid	1.000	0.834	1.279	1.725	0.9945	0.8944	0.9752	0.5159
		2-Ketoglutamic acid	1.000	1.441	1.556	1.296	0.1507	0.5091	0.0524	0.8587
		Succinic acid	1.000	1.067	1.316	1.075	0.9832	0.5321	0.3552	>0.9999
		Fumaric acid	1.000	1.631	1.408	1.543	0.0191	0.8695	0.1711	0.9569
		D-Malic acid	1.000	2.752	1.657	1.587	0.0024	0.9976	0.3747	0.0313
Amino acids and AA derivatives		L-Valine	1.000	1.429	1.535	1.549	0.1788	0.9999	0.0701	0.9166
		L-Leucine	1.000	1.479	1.907	1.545	0.5306	0.6930	0.0825	0.9969
		L-Isoleucine	1.000	1.295	1.555	1.654	0.6279	0.9722	0.1452	0.4239
		Glycine	1.000	1.124	1.041	1.340	0.7426	0.0849	0.9862	0.2792
		L-Alanine	1.000	2.093	1.595	1.843	0.0049	0.7624	0.1631	0.7563
		L-Proline	1.000	1.482	1.491	2.068	0.5558	0.3614	0.5415	0.3489
		L-Phenylalanine	1.000	1.687	1.956	2.024	0.1410	0.9948	0.0269	0.6369
		L-Tyrosine	1.000	1.320	1.635	2.099	0.4910	0.1606	0.0511	0.0096
		L-Serine	1.000	2.872	1.937	1.842	0.0005	0.9917	0.0774	0.0339
		L-Threonine	1.000	2.028	1.651	2.657	0.0029	0.0021	0.0637	0.0560
		L-Methionine	1.000	3.254	1.710	2.311	0.0001	0.3502	0.2649	0.0715
		L-Asparagine	1.000	0.874	1.080	1.618	0.9866	0.4533	0.9964	0.1991
		L-Aspartic acid	1.000	3.974	1.998	1.551	0.0012	0.8673	0.3988	0.0041
		L-Glutamic acid	1.000	0.913	1.370	2.416	0.9970	0.0906	0.8280	0.0107
		L-Lysine	1.000	12.134	2.328	10.184	0.7020	0.8628	0.9998	0.9492
		Pyroglutamic acid	1.000	1.289	1.245	1.412	0.0633	0.3619	0.1334	0.6075
		Ornithine	1.000	1.147	1.273	1.098	0.4858	0.2914	0.0690	0.9534
		Taurine	1.000	1.264	0.733	0.997	0.9826	0.9793	0.9820	0.9788
		Beta-Alanine	1.000	1.125	1.183	0.811	0.9170	0.2242	0.7865	0.3538
		L-Threonine2	1.000	1.981	1.754	2.020	<0.0001	0.2287	0.0004	0.9906
		Iminodiacetic acid 1	1.000	0.743	1.142	1.141	0.6786	>0.9999	0.1848	0.8084
		4-Hydroxyproline	1.000	1.398	1.469	1.303	0.0369	0.5490	0.0129	0.8660
Lipid metabolism		Capric acid	1.000	1.139	1.188	1.099	0.4789	0.7539	0.2358	0.9693
		Myristic acid	1.000	1.043	1.108	0.947	0.9953	0.7939	0.9355	0.9450
		Lauric Acid	1.000	0.969	1.223	1.118	0.9886	0.6680	0.1446	0.3983
		Palmitoleic acid	1.000	0.855	1.775	1.058	0.9702	0.1391	0.1287	0.9114
		Palmitic acid	1.000	1.110	1.332	1.200	0.7269	0.5612	0.0303	0.8036
		Linoleic acid	1.000	1.415	1.342	1.284	0.4020	0.9951	0.5605	0.9481
		Oleic acid	1.000	1.195	1.487	1.155	0.8225	0.4279	0.1802	0.9974
		Elaidic acid	1.000	0.537	0.809	0.832	0.1170	0.9992	0.7542	0.3913
		Stearic acid	1.000	1.006	1.132	1.063	>0.9999	0.9339	0.7185	0.9609
		Arachidic acid	1.000	1.046	1.227	1.119	0.9876	0.8497	0.4102	0.9480
		Arachidonic Acid	1.000	0.655	0.901	0.958	0.9258	0.4499	0.9720	0.9806
		Oxalic acid	1.000	1.082	1.249	1.084	0.9800	0.8450	0.6568	>0.9999
		Heptadecanoic acid	1.000	0.953	1.087	1.079	0.9638	0.9997	0.8207	0.5563
		1-Monomyristin	1.000	1.341	2.091	1.470	0.6595	0.1580	0.0101	0.9655
		2-Monopalmitoylglycerol	1.000	0.917	1.170	1.179	0.9488	>0.9999	0.6971	0.3133
		1-Palmitoylglycerol	1.000	0.907	1.156	1.204	0.9180	0.9850	0.7124	0.1798

	2-Steroylglycerol	1.000	1.160	0.866	1.939	0.9953	0.7363	0.9968	0.6946
	1-Steroylglycerol	1.000	1.035	1.250	1.272	0.9960	0.9987	0.4099	0.4031
	Glycerol	1.000	1.311	1.500	1.277	0.3419	0.5679	0.0599	0.9971
	Cholesterol	1.000	0.865	0.880	0.982	0.3880	0.5721	0.4851	0.4619
	3-Hydroxybutyrate	1.000	1.485	1.829	1.694	0.9963	>0.9999	0.7406	0.8228
Inorganic phosphate	Phosphate	1.000	1.118	1.036	1.244	0.4358	0.0500	0.9635	0.3366
	Pyrophosphate	1.000	0.852	1.017	1.427	0.7632	0.0505	0.9994	0.0053
Purines & Purimidynes	Hypoxanthine	1.000	1.170	1.049	1.604	0.9496	0.2910	0.9986	0.4924
	Adenosine	1.000	1.809	1.223	2.320	0.7835	0.5453	0.9936	0.9207
	Inosine	1.000	0.831	1.096	1.433	0.9805	0.8513	0.9962	0.4962
	Adenine	1.000	1.303	1.399	1.417	0.5028	0.9997	0.2783	0.9407
	Adenosine monophosphate	1.000	0.840	0.756	2.364	0.9289	<0.0001	0.7937	0.0001
	Thymine	1.000	1.509	1.137	1.277	0.1556	0.9122	0.9290	0.7028
	Uracil	1.000	1.302	1.391	1.261	0.3363	0.8536	0.1522	0.9940
	Urea	1.000	1.171	1.105	1.165	0.6085	0.9645	0.8705	>0.9999
Others	Nicotinamide	1.000	1.069	0.718	1.130	0.9710	0.0602	0.3135	0.9752
	Creatinine	1.000	1.196	1.405	1.138	0.6956	0.4149	0.1492	0.9856
	Glutathione	1.000	1.678	1.050	1.567	0.6697	0.7924	0.9998	0.9971
	L-Ascorbic acid	1.000	1.035	1.957	1.299	0.9989	0.0525	0.0067	0.6682
	Galactonic acid	1.000	6.626	1.146	0.656	0.0130	0.8747	0.9816	0.0039
	Cellobiose	1.000	3.248	1.448	1.007	0.0038	0.8181	0.8362	0.0024
	D-Threitol	1.000	0.999	1.360	1.011	>0.9999	0.0475	0.0549	0.9996
	Threonic acid	1.000	1.789	1.328	1.446	0.0006	0.8480	0.1885	0.1275
	Nonanoic acid	1.000	1.230	1.319	1.213	0.2546	0.7808	0.0718	0.9987
	O-Phosphoethanolamine	1.000	1.354	1.297	1.893	0.4627	0.0740	0.6022	0.1168
	Ethanolamine1	1.000	0.874	0.937	1.060	0.8836	0.8753	0.9832	0.6711
	Allantoin	1.000	1.133	0.665	0.573	0.7719	0.9359	0.9953	>0.9999
	2-Hydroxyglutaric acid	1.000	1.716	1.376	1.271	0.0370	0.9635	0.4089	0.2293
	2-Hydroxybutyric acid	1.000	1.140	1.162	1.209	0.7529	0.9842	0.6618	0.9524
	Glycolic acid	1.000	1.495	1.283	1.253	0.0158	0.9958	0.2339	0.3104
	Putrescine	1.000	0.872	0.795	1.097	0.8349	0.1977	0.5512	0.4260
	1,6-anhydro-Glucose	1.000	1.028	1.167	1.307	0.9990	0.8811	0.8375	0.4757
	1-Octadecene	1.000	1.280	1.073	1.115	0.3334	0.9918	0.9674	0.7002
	2-Ketoisocaproic acid	1.000	4.501	2.287	0.612	0.5018	0.9482	0.9849	0.4072
	Adipic acid	1.000	1.329	1.257	1.412	0.0303	0.4232	0.1085	0.8343
	Azelaic acid	1.000	1.443	1.507	1.117	0.0967	0.1300	0.0497	0.2434
	Benzoic acid	1.000	1.182	1.354	1.018	0.2914	0.0119	0.0126	0.3266
	Cystathionine	1.000	1.209	0.869	0.793	0.8717	0.9878	0.9906	0.8587
	Eicosane	1.000	0.881	1.296	1.215	0.9136	0.9368	0.2337	0.8667
	Ethanolamine2	1.000	1.160	1.355	1.334	0.6793	0.9986	0.0997	0.5733
	Glyoxylic acid	1.000	0.992	1.018	1.072	>0.9999	0.9664	0.9990	0.9045
	Itaconic acid	1.000	0.984	1.022	1.065	0.9993	0.9827	0.9980	0.9015
	methyl-beta-D-Galactopyranoside	1.000	1.925	1.280	1.229	0.0011	0.9912	0.4752	0.0071
	Methylmalonic acid	1.000	1.438	1.333	1.122	0.0895	0.5706	0.2503	0.2443
	N-acetyl-L-aspartic acid	1.000	0.765	0.625	0.528	0.9531	0.9468	0.9692	0.4826
	N-acetyl-Lysine	1.000	6.238	4.342	8.749	0.6205	0.7877	0.9811	0.9899
	Nitrous Oxide	1.000	1.682	1.638	0.910	0.8396	0.7848	0.8641	0.7539
	Pyrrolidonecarboxylic acid	1.000	1.849	2.075	1.238	0.2190	0.1884	0.0874	0.4323
	Sophorose	1.000	1.050	1.418	1.337	0.9910	0.9579	0.1144	0.3275
	Trehalose	1.000	1.014	0.719	0.923	0.7428	0.9762	0.9984	0.8569
	Turanose	1.000	2.457	1.214	1.014	0.0064	0.9385	0.9373	0.0043
	alpha-D-Glucosamine 1-phosphate	1.000	1.580	1.583	1.831	0.0591	0.5963	0.0576	0.5875

[A] *P* value was calculated by 1-way ANOVA with Tukey's test.

Supplemental Table 3. Relative mRNA and protein expression of glucose metabolic factors in NRVMs transduced with Ad-shControl or Ad-shYap

Name	mRNA		Protein	
	shControl	shYap	shControl	shYap
Glut1	1.00 ± 0.10	1.37 ± 0.13	1.00 ± 0.09	1.07 ± 0.12
Glut4	1.00 ± 0.08	1.43 ± 0.12[A]	1.00 ± 0.13	0.98 ± 0.16
Hk1	1.00 ± 0.15	1.14 ± 0.08	1.00 ± 0.05	1.02 ± 0.07
Hk2	1.00 ± 0.15	1.3 ± 0.13[A]	1.00 ± 0.09	0.97 ± 0.14
Gpi	1.00 ± 0.10	0.96 ± 0.06	1.00 ± 0.08	1.01 ± 0.12
Pfk1	1.00 ± 0.11	2.3 ± 0.08[A]	1.00 ± 0.02	1.17 ± 0.04[A]
Pfk2	1.00 ± 0.10	0.89 ± 0.05	1.00 ± 0.13	1.13 ± 0.06
Aldoa	1.00 ± 0.12	0.98 ± 0.07	1.00 ± 0.08	0.75 ± 0.08
Gapdh	1.00 ± 0.07	0.81 ± 0.05	1.00 ± 0.04	1.04 ± 0.03
Pgk1	1.00 ± 0.03	0.73 ± 0.03[A]	1.00 ± 0.06	0.69 ± 0.04[A]
Pgam1	1.00 ± 0.09	0.67 ± 0.05[A]	1.00 ± 0.05	0.76 ± 0.06[A]
Eno1	1.00 ± 0.07	0.71 ± 0.05[A]	1.00 ± 0.05	0.80 ± 0.06[A]
Pkm1	1.00 ± 0.09	1.75 ± 0.16[A]	1.00 ± 0.04	0.89 ± 0.09
Pkm2	1.00 ± 0.10	0.66 ± 0.04[A]	1.00 ± 0.03	0.99 ± 0.04
Ldha	1.00 ± 0.10	1.09 ± 0.03	1.00 ± 0.09	1.05 ± 0.08
Pdh	1.00 ± 0.09	0.69 ± 0.04[A]	1.00 ± 0.08	0.89 ± 0.07
Pdk4	1.00 ± 0.06	1.44 ± 0.10[A]	1.00 ± 0.03	0.94 ± 0.06
Pc	1.00 ± 0.10	1.25 ± 0.10	1.00 ± 0.05	0.93 ± 0.09
Me1	1.00 ± 0.06	1.07 ± 0.08	1.00 ± 0.05	0.86 ± 0.08
Pck2	1.00 ± 0.04	0.69 ± 0.03[A]	1.00 ± 0.02	0.86 ± 0.03[A]
G6pd	1.00 ± 0.14	0.96 ± 0.12	1.00 ± 0.14	0.87 ± 0.09
Gfat	1.00 ± 0.07	0.91 ± 0.05	1.00 ± 0.17	1.04 ± 0.11
Gpd	1.00 ± 0.10	1.52 ± 0.15[A]	1.00 ± 0.02	0.98 ± 0.05
Phgdh	1.00 ± 0.12	0.94 ± 0.04	1.00 ± 0.07	1.12 ± 0.05

Mean values plus SEM are indicated in each cell. n = 5–6 dishes from 5–6 independent experiments. [A]$p < 0.05$ versus each shControl by Student's *t* test. See also Supplemental Figure 8D.

Supplemental Table 4. Metabolomics analysis of energy metabolism pathways in NRVMs transduced with Ad-LacZ or Ad-YAP

Pathway		Metabolite	Fold change		*P* value
			LacZ (n = 6)	YAP (n = 6)	
Glucose metabolism	Monosaccharide	D-Glucose	1.000	1.121	0.718
		D-Fructose	1.000	1.798	0.039
		D-Mannose	1.000	1.869	0.053
	Sugar alcohol (Polyol)	Mannitol	1.000	0.751	0.106
		D-Threitol	1.000	1.755	0.000
		Sorbitol	1.000	2.171	0.009
	Glycolytic intermediates	Glucose-6-phosphate	1.000	0.412	0.150
		Fructose-6-phosphate	1.000	0.382	0.118
		Glycerol	1.000	0.808	0.583
		Glycerol 3-phosphate	1.000	0.718	0.023
		DHAP	1.000	1.318	0.583
		3-Phosphoglyceric acid	1.000	1.945	0.041
		Phosphoenolpyruvate	1.000	3.807	0.002
		Pyruvate	1.000	0.642	0.179
		L-Lactic acid	1.000	1.366	0.406
	Pentose phosphate pathway	Sedoheptulose-7-phosphate	1.000	0.622	0.360
		Ribitol	1.000	0.629	0.001
TCA cycle		Citric acid	1.000	0.990	0.970
		Isocitric acid	1.000	0.759	0.593
		cis-Aconitic acid	1.000	1.007	0.973
		2-Ketoglutarate	1.000	0.159	0.047
		Succinic acid	1.000	1.249	0.108
		D-Malic acid	1.000	2.445	0.003
		Fumaric acid	1.000	1.497	0.108
Amino acids and AA derivatives		L-Valine	1.000	1.269	0.254
		L-Leucine	1.000	1.363	0.102
		L-Isoleucine	1.000	1.452	0.027
		L-Alanine	1.000	1.521	0.012
		L-Aspartic acid	1.000	1.250	0.705
		L-Glutamine	1.000	0.787	0.659
		L-Glutamic acid	1.000	1.078	0.701
		Glycine	1.000	1.047	0.610
		L-Lysine	1.000	1.720	0.018
		L-Methionine	1.000	1.601	0.001
		L-Proline	1.000	1.137	0.611
		L-Phenylalanine	1.000	1.387	0.023
		L-Serine	1.000	1.187	0.432
		L-Threonine	1.000	1.385	0.001
		L-Tyrosine	1.000	1.625	0.015
		beta-Alanine	1.000	1.017	0.965
		2-Aminoadipic acid	1.000	0.979	0.941
		Hypotaurine	1.000	0.928	0.627
		Pyroglutamic acid	1.000	1.374	0.127
		N-acetyl-L-Aspartic acid	1.000	0.378	0.053
Lipids		Lauric acid	1.000	1.291	0.334
		Myristic acid	1.000	1.188	0.144
		Palmitic acid	1.000	0.985	0.908
		Palmitoleic acid	1.000	1.099	0.767
		Oleic acid	1.000	0.790	0.331
		Stearic acid	1.000	0.941	0.665

	1-Palmitoylglycerol	1.000	1.072	0.444
	1-Steroylglycerol	1.000	1.091	0.471
	2-Monopalmitoylglycerol	1.000	1.040	0.772
	Cholesterol	1.000	0.692	0.625
	O-Phosphoethanolamine	1.000	0.265	0.000
inorganic phosphate	Phosphate	1.000	1.187	0.333
	Pyrophosphate	1.000	1.038	0.893
Urea cycle	Ornithine	1.000	1.820	0.149
Purine and Pyrimidine	Adenosine	1.000	0.842	0.488
	Inosine	1.000	1.161	0.701
	Adenine	1.000	3.358	0.078
	Adenosine monophosphate	1.000	1.095	0.637
	Thymine	1.000	1.255	0.306
	Uracil	1.000	1.026	0.804
	Uridine	1.000	0.808	0.685
Ketone body	2-Hydroxyglutaric acid	1.000	0.669	0.553
	3-Hydroxybutyric acid	1.000	0.991	0.982
Others	Creatinine	1.000	1.153	0.586
	myo-Inositol	1.000	0.363	0.121
	Inositol phosphate	1.000	1.037	0.918
	Pantothenic acid	1.000	1.188	0.361
	Gluconic acid	1.000	0.525	0.054
	Nonanoic acid	1.000	1.154	0.377
	Capric acid	1.000	0.993	0.965
	Heptadecanoic acid	1.000	0.968	0.904
	2'3'-Bisphosphoglycerate	1.000	0.979	0.951
	2-Hydroxybenzoic acid	1.000	1.059	0.794
	Adipic acid	1.000	1.268	0.253
	Benzoic acid	1.000	1.048	0.692
	Cystathionine	1.000	4.314	0.001
	Disaccharide	1.000	0.740	0.623
	Ethanolamine	1.000	1.124	0.318
	Ethanolamine	1.000	1.123	0.519
	Glyoxylic acid	1.000	0.419	0.055
	Nicotinamide	1.000	1.250	0.026
	Nitrous Oxide	1.000	1.158	0.269
	Oxalic acid	1.000	1.167	0.254
	Putrescine	1.000	2.316	0.207
	Pyrrolidonecarboxylic acid	1.000	1.301	0.187
	Sarcosine	1.000	1.248	0.031
	Threonic acid	1.000	1.111	0.578

P value was calculated by 2-tailed unpaired Student's *t* test.

Supplemental Table 5. Antibodies Used in This Paper

Antibody	Source	Identifier	Dilution
Anti-GLUT1	Cell Signaling Technology	12939	WB 1:1,000
Anti-GLUT4	Cell Signaling Technology	2213	WB 1:1,000
Anti-HK1	Cell Signaling Technology	2024	WB 1:1,000
Anti-HK2	Cell Signaling Technology	2867	WB 1:1,000
Anti-GPI	Cell Signaling Technology	57893	WB 1:1,000
Anti-PFK1	Novus Biologicals	NBP2-75578	WB 1:1,000
Anti-PFK2	Cell Signaling Technology	13123	WB 1:1,000
Anti-ALDOA	Cell Signaling Technology	8060	WB 1:1,000
Anti-GAPDH	Cell Signaling Technology	2118	WB 1:3,000
Anti-PGK1	Abcam	ab154613	WB 1:1,000
Anti-PGAM1	Cell Signaling Technology	12098	WB 1:1,000
Anti-ENO1	Cell Signaling Technology	3810	WB 1:1,000
Anti-PKM1/2	Cell Signaling Technology	3190	WB 1:1,000
Anti-PKM1	Proteintech	15821-1-AP	WB 1:1,000
Anti-PKM2	Cell Signaling Technology	4053	WB 1:1,000
Anti-PKM2 (Tyr105)	Cell Signaling Technology	3827	WB 1:1,000
Anti-LDHA	Cell Signaling Technology	3582	WB 1:1,000
Anti-PDH	Cell Signaling Technology	3205	WB 1:1,000
Anti-MPC1	Cell Signaling Technology	14462	WB 1:1,000
Anti-MPC2	Cell Signaling Technology	46141	WB 1:1,000
Anti-PDK4	Novus Biologicals	NBP1-07047	WB 1:1,000
Anti-G6PD	Cell Signaling Technology	12263	WB 1:1,000
Anti-GFAT	Cell Signaling Technology	5322	WB 1:1,000
Anti-GPD	Abcam	ab153902	WB 1:1,000
Anti-PHGDH	Cell Signaling Technology	66350	WB 1:1,000
Anti-PHGDH	Novus Biologicals	NBP2-52939	WB 1:1,000
Anti-PCK2	Cell Signaling Technology	8565	WB 1:1,000
Anti-PC	Novus Biologicals	NBP1-49636	WB 1:1,000
Anti-ME1	GeneTex	GTX104122	WB 1:1,000
Anti-α-tubulin	Sigma-Aldrich	T6199	WB 1:6,000
Anti-YAP	Cell Signaling Technology	14074	WB 1:1,000
Anti-phospho-YAP (Ser127)	Cell Signaling Technology	4911	WB 1:1,000
Anti-TAZ	ABclonal	A15806	WB 1:1,000
Total OXPHOS Rodent WB Antibody Cocktail	Abcam	ab110413	WB 1:2,000
Anti-AMPK	Cell Signaling Technology	5832	WB 1:1,000
Anti-phospho-AMPK (Thr172)	Cell Signaling Technology	2535	WB 1:2,000
Anti-ACC	Cell Signaling Technology	3676	WB 1:1,000
Anti-phospho-ACC (Ser79)/(Ser212)	Cell Signaling Technology	11818	WB 1:1,000
Anti-Tri-Methyl-Histone H3 (Lys4)	Cell Signaling Technology	9751	WB 1:1,000
Anti-H3	Cell Signaling Technology	4499	WB 1:1,000
Anti-mouse IgG	Cell Signaling Technology	7076	WB 1:5,000
Anti-rabbit IgG	Cell Signaling Technology	7074	WB 1:5,000
Anti-TEAD1	Sigma-Aldrich	AV39521	WB 1:1,000
Anti-c-Myc	Cell Signaling Technology	5605	WB 1:1,000
Anti-HIF-1α	Cell Signaling Technology	79233	WB 1:1,000; IP 4 μg for 150 μg lysate
Control IgG	Cell Signaling Technology	5415	IP 4 μg for 150 μg lysate
Anti-FLAG	Sigma-Aldrich	F1804	IP 4 μg for 150 μg lysate
Anti-Myc-tag	MBL	M192-3	IP 4 μg for 150 μg lysate

Mouse TrueBlot ULTRA, Anti-mouse Ig HRP	ROCKLAND	18-8817-33	WB 1:5,000
Control IgG	Cell Signaling Technology	2729	ChIP 5 μL for 5 μg chromatin
Anti-YAP	Cell Signaling Technology	14074	ChIP 5 μL for 5 μg chromatin
Anti-TEAD1	Cell Signaling Technology	12292	ChIP 5 μL for 5 μg chromatin
Anti-HIF-1α	Cell Signaling Technology	36169	ChIP 5 μL for 5 μg chromatin
Anti-Ki-67	Cell Signaling Technology	9129	IHC 1:400
Anti-cardiac troponin T	Thermo Fisher Scientific	MA5-12960	IHC 1:400
Goat anti-mouse IgG, Alexa Fluor 488	Thermo Fisher Scientific	A-24920	IHC 1:400
Goat anti-rabbit IgG, Alexa Fluor 594	Thermo Fisher Scientific	A-11072	IHC 1:400

Supplemental Table 6. siRNAs Used in This Paper

siRNA Name	Source	Identifier
siTead1	Thermo Fisher Scientific	MSS211219
siHif-1α	Thermo Fisher Scientific	RSS310066
sic-Myc	Thermo Fisher Scientific	RSS351381
siGlut1	Thermo Fisher Scientific	RSS332476
siHk2	Thermo Fisher Scientific	RSS302994
siGpi	Thermo Fisher Scientific	RSS307944
siPfk1	Thermo Fisher Scientific	RSS368299
siAldoa	Thermo Fisher Scientific	RSS332289
siGapdh	Thermo Fisher Scientific	s236445
siPgk1	Thermo Fisher Scientific	s128192
siPgam1	Thermo Fisher Scientific	RSS351593
siEno1	Thermo Fisher Scientific	s127698
siPkm1	Thermo Fisher Scientific	RSS340844
siG6pd	Thermo Fisher Scientific	RSS302151
siGfat	Thermo Fisher Scientific	RSS334938
siGpd1	Thermo Fisher Scientific	RSS339932
siPhgdh	Thermo Fisher Scientific	RSS339816
siPck2	Thermo Fisher Scientific	RSS323287
siControl	Thermo Fisher Scientific	12935300

Supplemental Table 7. Oligonucleotide Primers Used in This Paper

Name	Forward Primer	Reverse Primer
qPCR primers		
Mouse *Glut1*	TGTGCTTCCAGTATGTGGAGC	CTTGTCACTTTGGCTGGCAC
Mouse *Glut4*	TATGTTGCGGATGCTATGGGTC	CTGGGTTTCACCTCCTGCTC
Mouse *Hk1*	TCCATCCACACTTCTCCAGAATC	GATCCTGGCTCTTAGGCGTTC
Mouse *Hk2*	TCATTGTTGGCACTGGAAGC	TTGCCAGGGTTGAGAGAGAG
Mouse *Gpi*	TGATTGCCATGTATGAGCACAAG	AGTGGAGGAATCATGGGAGG
Mouse *Pfk1*	ACAGACTTTGAACACCGAATCC	GCTTCTCCAGACCGTTTCCTTG
Mouse *Pfk2*	CCTGGGAAAGAACATGAAGAGC	CCATTCTCCCGAGTCCAGAAG
Mouse *Aldoa*	GGCATTAAGGTAGATAAGGGTGTG	GCATAACGGGCCAGAACATTG
Mouse *Gapdh*	CTACACTGAGGACCAGGTTGTCT	CACCACCCTGTTGCTGTAGCC
Mouse *Pgk1*	TTGTTTGGAATGGTCCTGTTGG	GCTGACTTTATCCTCTGTGTTCC
Mouse *Pgam1*	TCAGCAAGGATCGCAGGTAC	AGACCCTCCAGATGCTTAACG
Mouse *Eno1*	GCTCTGTGACCGAATCCCTG	ACCAGTCTTGATCTGCCCAGTG
Mouse *Pkm1*	GTCTGGAGAAACAGCCAAGG	TCTTCAAACAGCAGACGGTG
Mouse *Pkm2*	GTCTGGAGAAACAGCCAAGG	CGGAGTTCCTCGAATAGCTG
Mouse *Ldha*	CAAGCAGGTGGTGGACAGTG	TGGGACACTGAGGAAGACATC
Mouse *Mpc1*	TGACTTTCGCCCTCTGTTGC	GCCGCTTACTCATCTCGTAGTTG
Mouse *Mpc2*	GGGATTGGTATGTGCTGGATTAG	CCTTGACCAAATAAACCCTGTAGC
Mouse *Pdh*	AGGAGATTGATGTGGAAGTGAGG	CCACCTAACAGTCACCCATTAAC
Mouse *Pdk4*	ATCAAGATTTCTGACCGAGGAGG	TGGCGTAGAGACGAGAAATTGG
Mouse *G6pd*	AAGAATGTGAAGCTCCCTGATGC	CGCGGCTGCCATATACATAGG
Mouse *Gfat*	GACTGCCGGACTTGATTAAGG	TCACCAGCAAGGATGCCTTC
Mouse *Gpd*	CTTGAAGAATATAGTGGCCGTTGG	CAGGTCGTGATGAGGTCTGC
Mouse *Phgdh*	CCCACTATGATTGGCCTCCTG	CCTCCAATACATGCTGCTTCC
Mouse *Pc*	TGCAGAGGAGTTTGAGGTTGAG	TGCATCTCCTTCATGGCCTG
Mouse *Me1*	TGCCCTCATAGGAGTTGCTG	TCCCTTGGTCACCTTGTAGC
Mouse *Me2*	TAGAAGCTGCAAAGGCGCTG	CGTATCTGGCCTTGTCCTCG
Mouse *Pck2*	TGCGGAGCACAAAGGAAAGAC	CACGAGCATTCTCTCCAAAGC
Mouse *Ctgf*	CAAAGCAGCTGCAAATACCA	GGCCAAATGTGTCTTCCAGT
Mouse *Cyr61*	GAGGCTTCCTGTCTTTGGCAC	GATCCGGGTCTCTTTCACCAG
Mouse *Nppb*	GGTCCAGCAGAGACCTCAAA	CAACTTCAGTGCGTTACAGCC
Mouse *Yap*	CCCTGACTCCACAGCATGTTC	TTTGCCATCTCCCAACCTGC
Rat *Glut1*	AATATGTGGAGCAACTGTGTGG	TCAGGTGTCTTGTCGCTCTGG
Rat *Glut4*	TATGTTGCGGATGCTATGGGTC	TAAGAGAGAAGGTGTCCGTCG
Rat *Hk1*	CTTCATCCACACTTCTCCAGAATC	TAGGCGATCGAAGGGTCTCC
Rat *Hk2*	GTCTCAGATAGAGAGCGACTGC	ACGGTTCTCTCTTATCTTGTCCAC
Rat *Gpi*	ACTGATTGCCATGTATGAGCAC	GGAGGAGTCATGGGAGGTTAC
Rat *Pfk1*	GCTGAGGAATGAGAAGTGCAAC	CTGTCAAAGGGAGTTGGGCTTC
Rat *Pfk2*	AAACAGGATGCAAAGAAGGGAC	CAGGGCAGCAGAAGTGGAAG
Rat *Aldoa*	ACTGAGAAGGTACTGGCAGC	ACGAAGTGCTGTGACGGTTG
Rat *Gapdh*	CTACACTGAGGACCAGGTTGTCT	CACCACCCTGTTGCTGTAGCC
Rat *Pgk1*	CTGGATGGGCTTGGACTGTG	TAGACGTGGCTTTCACCACC
Rat *Pgam1*	AAGCATCTGGAGGGTCTGTC	TTTACGCACGGTCTCCTCATC
Rat *Eno1*	TCAAGGACTACCCAGTGGTG	CTTTGAGCAGGAGGCAGTTG
Rat *Pkm1*	ACCGCCTGCTGTTTGAAGAG	AGCAATGATAGGAGCCCTTGG
Rat *Pkm2*	AGGCTGCCATCTACCACTTG	AGCAATGATAGGAGCCCTTGG
Rat *Ldha*	GGTGGTTGACAGTGCATACG	TGGGACGCTGAGGAAGACATC
Rat *Pdh*	AAGGAGATTGATGTGGAAGTGAGG	CGCCCATCCATCTAATACTCTCC
Rat *Pdk4*	TCAAGATTTCTGACCGAGGTGG	TAGAGACGGGAAATCGGCAAG
Rat *G6pd*	CGTAGTGATGAACTCAGGGAAGC	CCTCATACTGGAAGCCCACTC
Rat *Gfat*	GACTGCCGGACTTGATTAAGG	TCACCAGCAAGGATGCCTTC
Rat *Gpd*	ACCTCATCACGACCTGCTAC	AAGGGAAACTTGTCCACGAGG
Rat *Phgdh*	GGCCTCAATGTCACCACCTC	AGACAGCTCCGTTGAGCATC
Rat *Pc*	GGTCTTCAAGTTCTGTGAGGTG	GTCACCCGTGTAGGAGATGG
Rat *Me1*	AGGAGTGCTATAAAGTGACCAAGG	CTCAGTCCACAGGCCACTAC
Rat *Pck2*	GCTGCTGAACACAAGGGAAAG	CGGAACCAGTTGACATGGAAG
Rat *Ndufs1*	CGTTCCACCTCAGCTAACTATAAAAGACTTC	CTCCTCTACTGCCTGAGCGC
Rat *Sdhd*	CTGTGGTGGACTACTCTCTGGCT	CATCGTGGTAATTGAAGTAGCAAAGCCC
Rat *Uqcrc2*	GCGGCTGGAGATGTTATCAATG	ATCTCACTCAGGAAGCCCTC
Rat *Cox8a*	ATCACCATTGGGCTCACTTCC	GATCAAGGTGGGCTGGCAAG
Rat *Atp5k*	TTCGGCCGGTACTCCGCTCT	TTCTCTCTCAATCCGTTTCAACTCATC
Mouse and Rat *Rps15*	TTCGCAAGTTCACCTACC	GAGAACTCGCCCAGGTAGTGG
ChIP qPCR primers		
Rat *Glut1* promoter	CACTGGCTTACAGCCCTGAAC	GCAATCTGGGTTTGGATGCTAG

Mouse *Glut1* promoter	ACTCAGAGAGGTGTGCAAACCAG	CGGGGCGGGACTCTGAGA
Mouse *Glut1* -7kbp	TTGCCTCAAAATGAAAACTACC	TCCTTCTATCTTCTCTCTCCTGAG
Mouse *Glut1* -644bp	CCTCCCGTGGTCTCTCTAATG	GGTGTTTACAACCGCGTGTG
Mouse *Glut* 1 -138bp	TGCTTGTAGAGTGACGATCTGAG	ACGGACGCGCTGTAACTATG

Full unedited image for Figure 3E_YAP

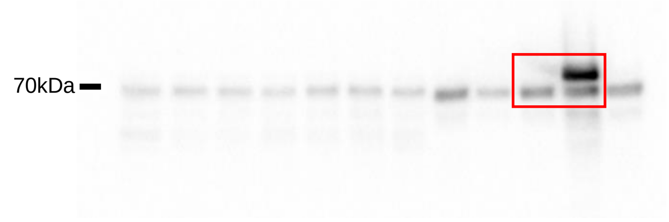

Full unedited image for Figure 3E_α-tubulin

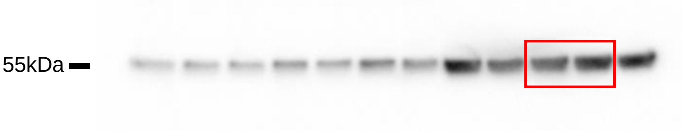

Full unedited image for Figure 5D_pY105-Pkm2

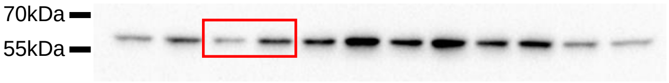

Full unedited image for Figure 5D_Pkm2

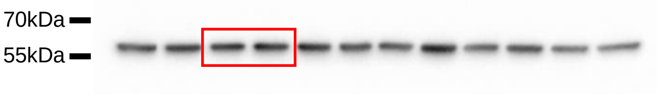

Full unedited image for Figure 5D_ α-tubulin

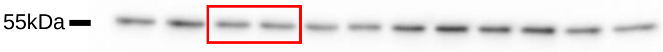

Full unedited image for Figure 8A_ YAP

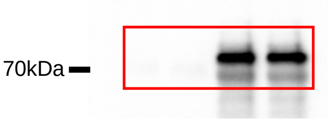

Full unedited image for Figure 8A_ Glut1

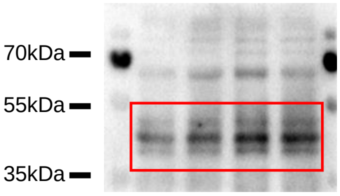

Full unedited image for Figure 8A_ α-tubulin

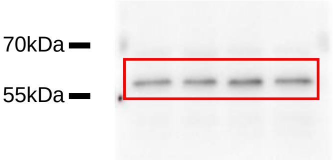

Full unedited image for Figure 9A_GLUT1

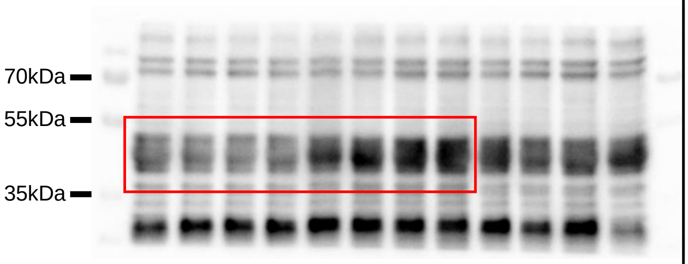

Full unedited image for Figure 9A_α-tubulin

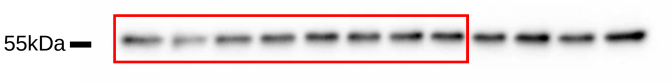

Full unedited image for Figure 9C_GLUT1

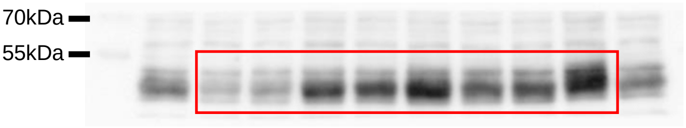

Full unedited image for Figure 9C_α-tubulin

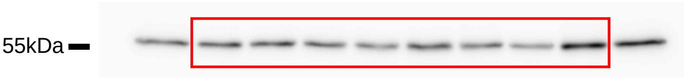

Full unedited image for Figure 11A_Tead1

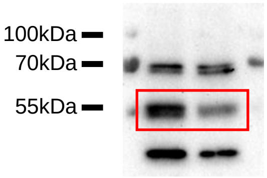

Full unedited image for Figure 11A_α-tubulin

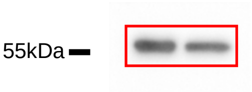

Full unedited image for Figure 11B_Glut1

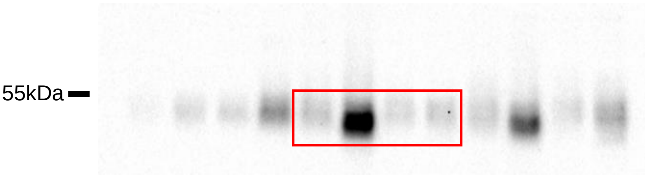

Full unedited image for Figure 11B_α-tubulin

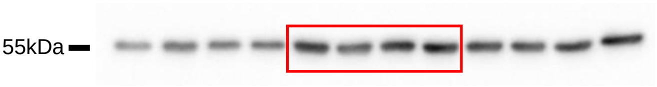

Full unedited image for Figure 11D_HIF-1α

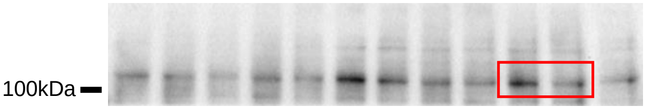

Full unedited image for Figure 11D_α-tubulin

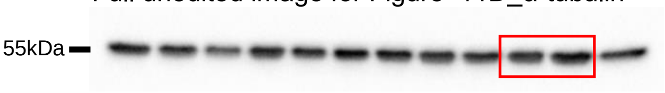

Full unedited image for Figure 11E_Glut1

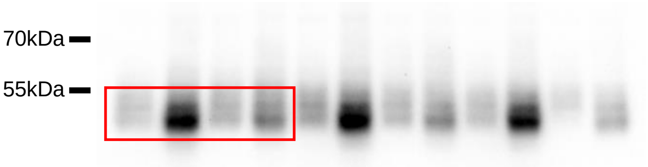

Full unedited image for Figure 11E_α-tubulin

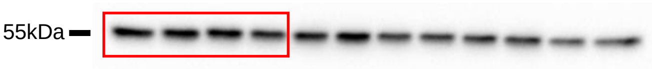

Full unedited image for Figure 11G_c-Myc

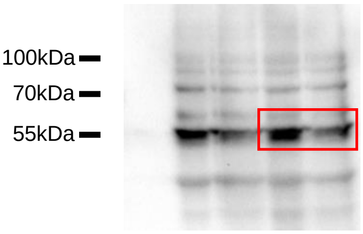

Full unedited image for Figure11G _ α-tubulin

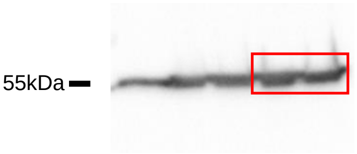

Full unedited image for Figure 11H_ Glut1

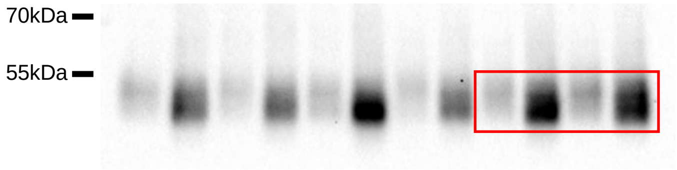

Full unedited image for Figure 11H_ α-tubulin

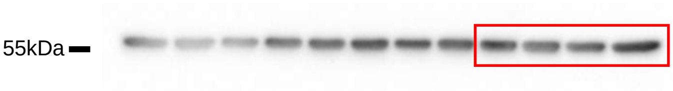

Full unedited image for Figure 11J_ Tead1

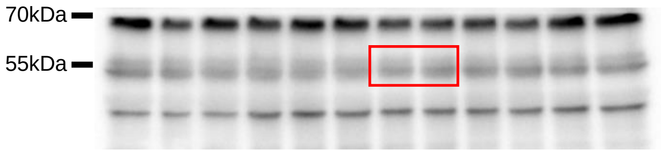

Full unedited image for Figure 11J_HIF-1α

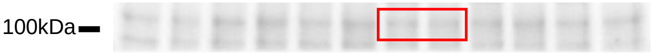

Full unedited image for Figure 11J_ c-Myc

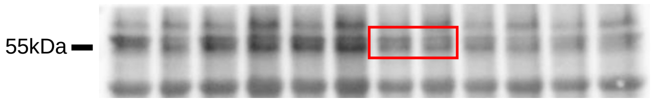

Full unedited image for Figure 11J_ α-tubulin

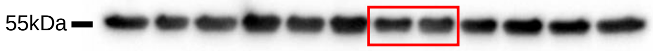

Full unedited image for Figure 11N_YAP

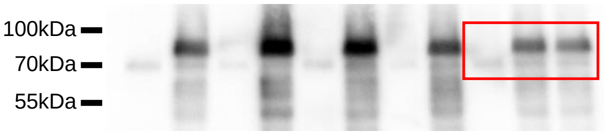

Full unedited image for Figure 11N_Glut1

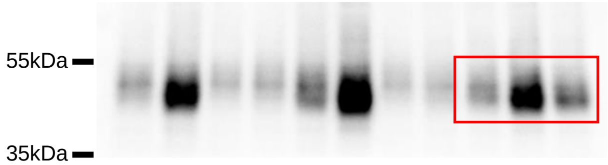

Full unedited image for Figure 11N_α-tubulin

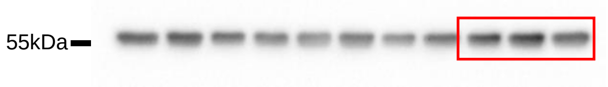

Full unedited image for Figure 13A_FLAG

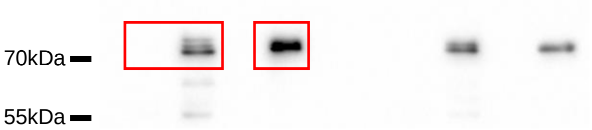

Full unedited image for Figure 13A_Myc

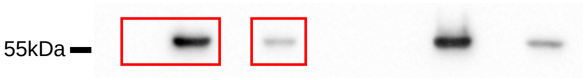

Full unedited image for Figure 13A_HIF-1α

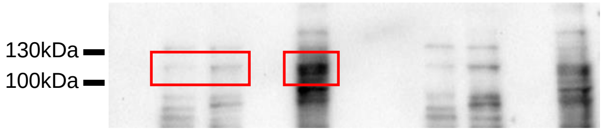

Full unedited image for Figure 13B_FLAG

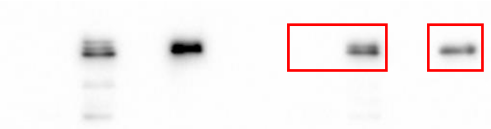

Full unedited image for Figure 13B_Myc

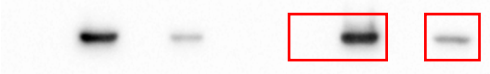

Full unedited image for Figure 13B_HIF-1α

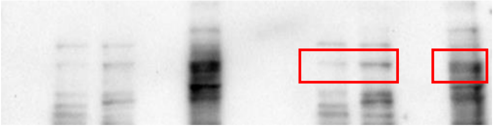

Full unedited image for Figure 13C_FLAG

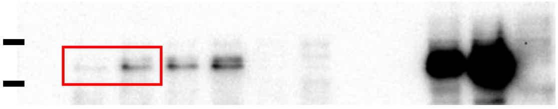

Full unedited image for Figure 13C_FLAG

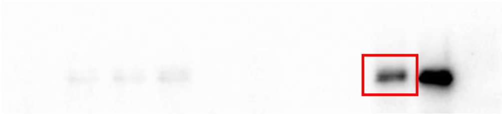

Full unedited image for Figure 13C_Myc

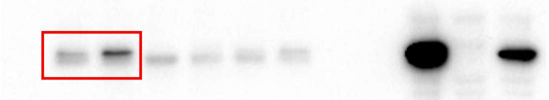

Full unedited image for Figure 13C_Myc

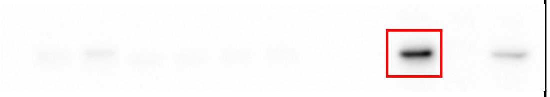

Full unedited image for Figure 13C_HIF-1α

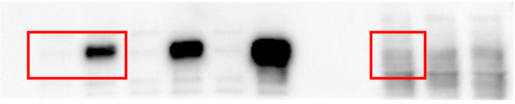

Full unedited image for Figure 13D _YAP

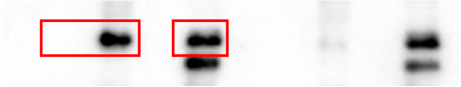

Full unedited image for Figure 13D _Myc

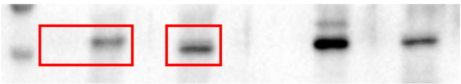

Full unedited image for Figure 13D _HIF-1α

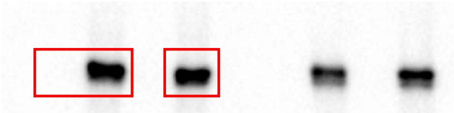

Full unedited image for Figure 13E_YAP

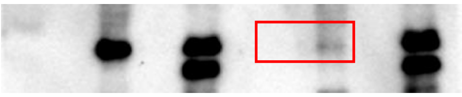

Full unedited image for Figure 13E_YAP

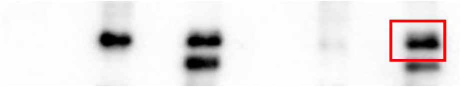

Full unedited image for Figure 13E_Myc

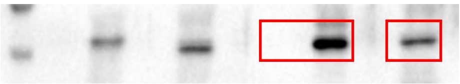

Full unedited image for Figure 13E_HIF-1α

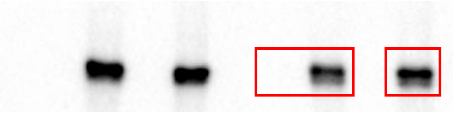

Full unedited image for Figure 13F_FLAG

70kDa
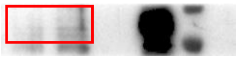

Full unedited image for Figure 13F_FLAG

70kDa
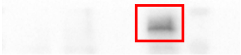

Full unedited image for Figure 13F_Myc

55kDa
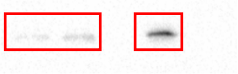

Full unedited image for Figure 13F_HIF-1α

100kDa
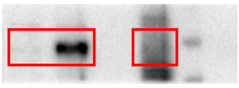

Full unedited image for Figure 13G_FLAG

70kDa
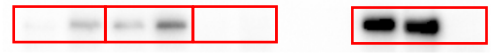

Full unedited image for Figure 13G_Myc

55kDa
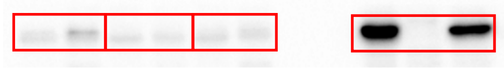

Full unedited image for Figure 13G_HIF-1α

100kDa
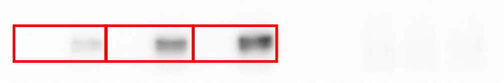

Full unedited image for Figure 13G_HIF-1α

100kDa
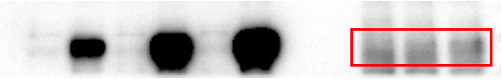

Full unedited image for Figure 13H_HIF-1α

100kDa
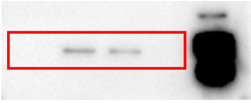

Full unedited image for Figure 13H_TEAD1

55kDa
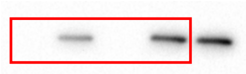

Full unedited image for Figure 13I_HIF-1α

100kDa
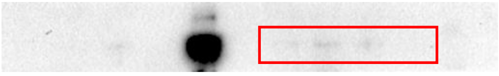

Full unedited image for Figure 13I_YAP

70kDa
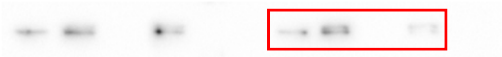

Full unedited image for Figure 13J _YAP

70kDa
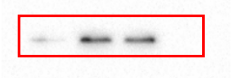

Full unedited image for Figure 13J_TEAD1

55kDa
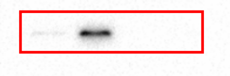

Full unedited image for Figure 13K_YAP

70kDa
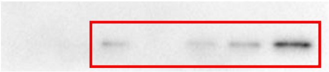

Full unedited image for Figure 13K_TEAD1

55kDa
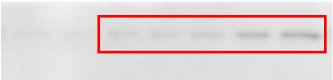

Full unedited image for Supplemental Figure 1Q_YAP

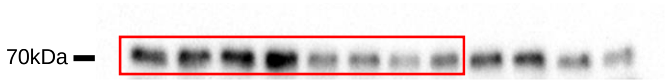

Full unedited image for Supplemental Figure 1Q_pS127-YAP

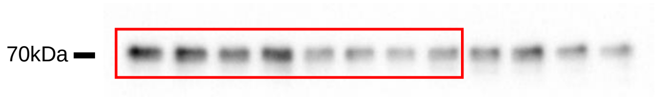

Full unedited image for Supplemental Figure 1Q_α-tubulin

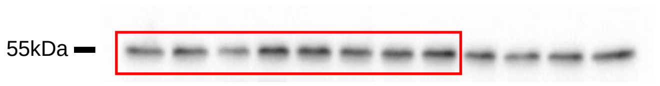

Full unedited image for Supplemental Figure 1R_TAZ

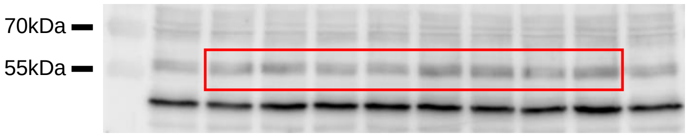

Full unedited image for Supplemental Figure 1R_α-tubulin

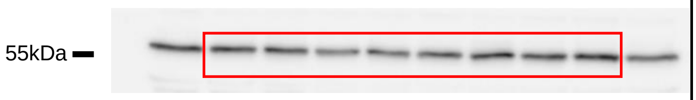

Full unedited image for Supplemental Figure 1S_AMPK

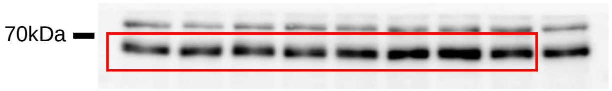

Full unedited image for Supplemental Figure 1S_pT172-AMPK

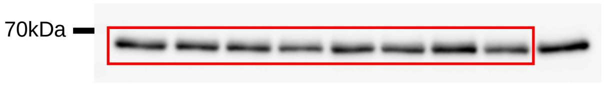

Full unedited image for Supplemental Figure 1S_ α-tubulin

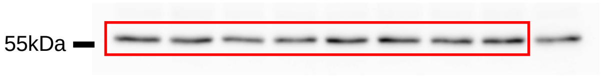

Full unedited image Supplemental Figure 1T_ACC1/2

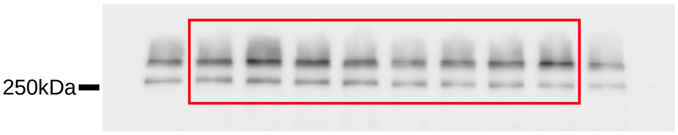

Full unedited image for Supplemental Figure 1T_pS79/S212-ACC1/2

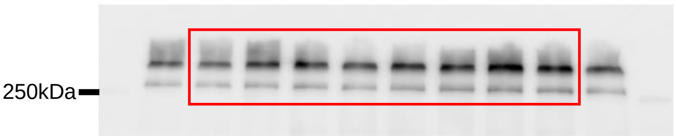

Full unedited image for Supplemental Figure 1T_α-tubulin

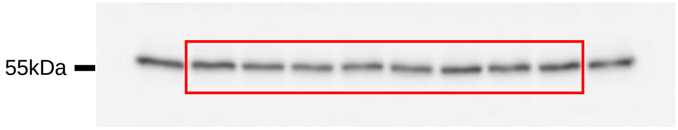

Full unedited image for Supplemental Figure 4_Glut1

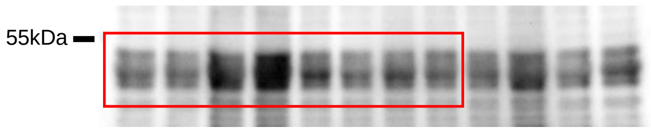

Full unedited image for Supplemental Figure 4_Glut4

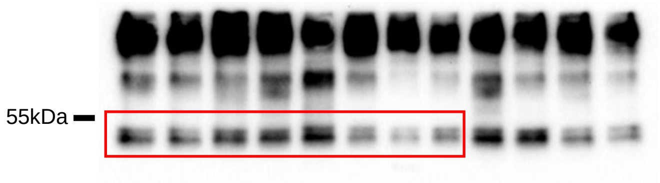

Full unedited image for Supplemental Figure 4_Hk2

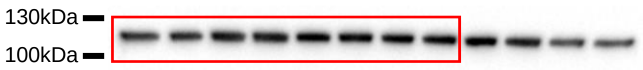

Full unedited image for Supplemental Figure 4_Pfk1

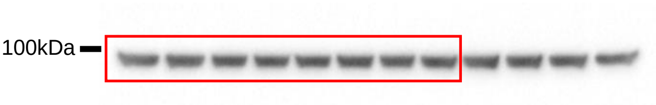

Full unedited image for Supplemental Figure 4_Gapdh

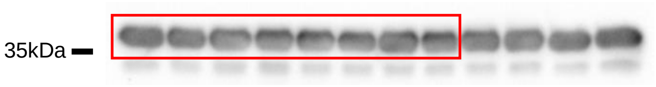

Full unedited image for Supplemental Figure 4_Pgk1

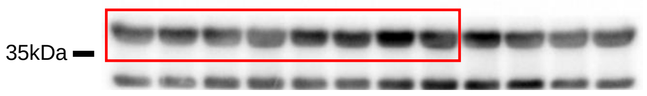

Full unedited image for Supplemental Figure 4_Pgam1

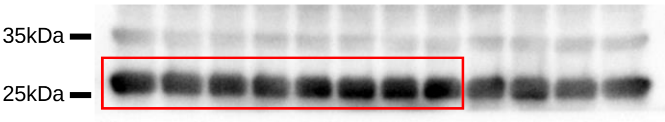

Full unedited image for Supplemental Figure 4_Pkm1/2

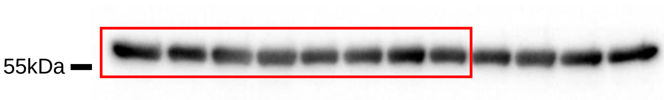

Full unedited image for Supplemental Figure 4_Pkm2

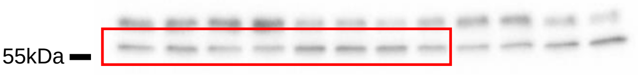

Full unedited image for Supplemental Figure 4_Ldha

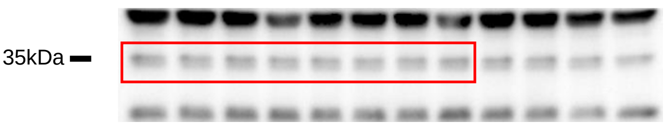

Full unedited image for Supplemental Figure 4_Pdh

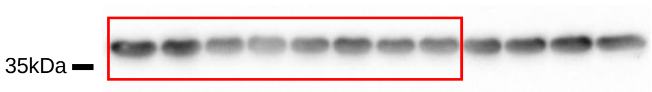

Full unedited image for Supplemental Figure 4_Pck2

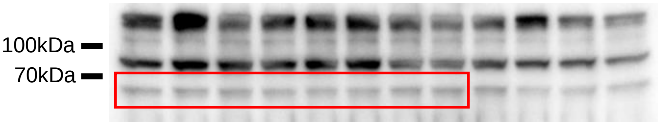

Full unedited image for Supplemental Figure 4_G6pd

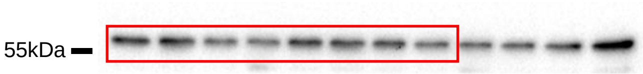

Full unedited image for Supplemental Figure 4_Gfat

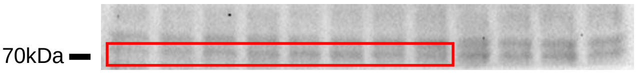

Full unedited image for Supplemental Figure 4_Gpd

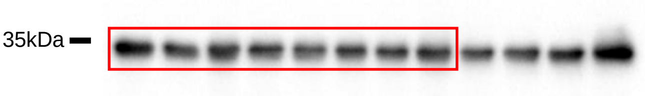

Full unedited image for Supplemental Figure 4_Phgdh

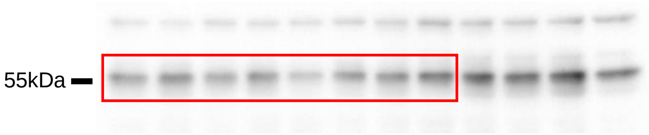

Full unedited image for Supplemental Figure 4_Tead1

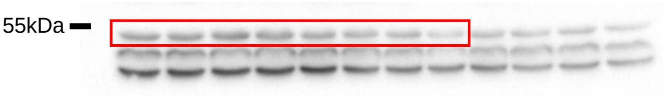

Full unedited image for Supplemental Figure 4_Hif-1α

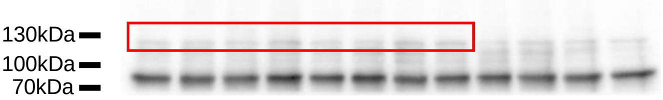

Full unedited image for Supplemental Figure 4_α-tubulin

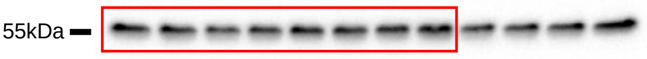

Full unedited image for Supplemental Figure 4_Mpc1

15kDa

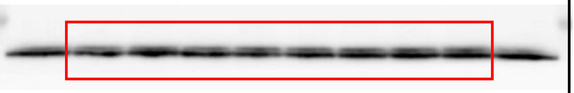

Full unedited image for Supplemental Figure 4_Mpc2

15kDa

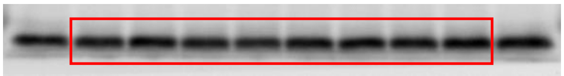

Full unedited image for Supplemental Figure 4_Pdk4

55kDa

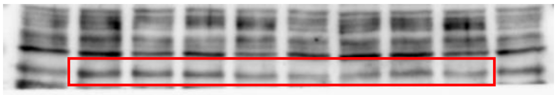

Full unedited image for Supplemental Figure 4_Pc

130kDa

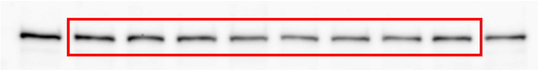

Full unedited image for Supplemental Figure 4_Me1

70kDa

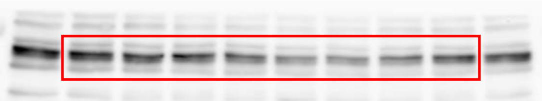

Full unedited image for Supplemental Figure 4_α-tubulin

55kDa

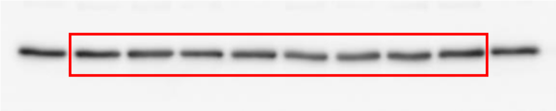

Full unedited image for Supplemental Figure 6_Glut1

55kDa

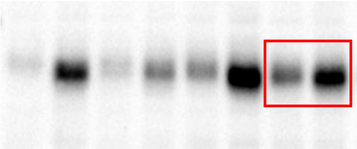

Full unedited image for Supplemental Figure 6_α-tubulin

55kDa

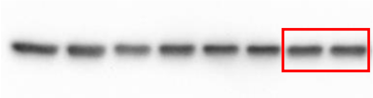

Full unedited image for Supplemental Figure 6_Hk1

130kDa

100kDa

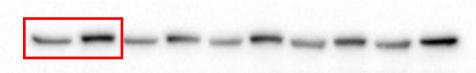

Full unedited image for Supplemental Figure 6_Pfk2

70kDa

55kDa

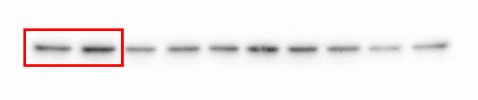

Full unedited image for Supplemental Figure 6_α-tubulin

55kDa

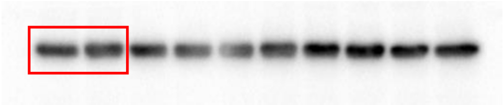

Full unedited image for Supplemental Figure 6_Glut4

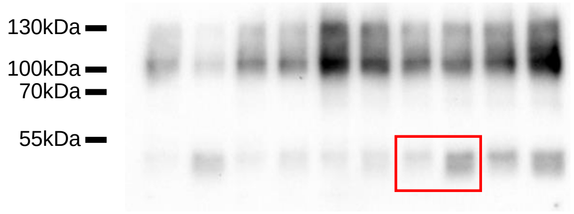

Full unedited image for Supplemental Figure 6_Hk2

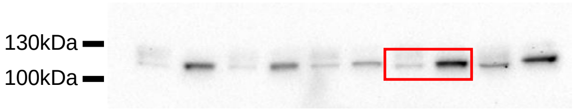

Full unedited image for Supplemental Figure 6_Pfk1

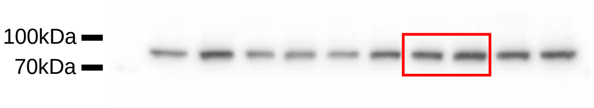

Full unedited image for Supplemental Figure 6_Eno1

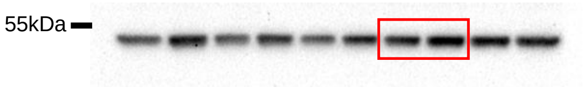

Full unedited image for Supplemental Figure 6_G6pd

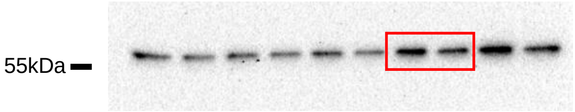

Full unedited image for Supplemental Figure 6_α-tubulin

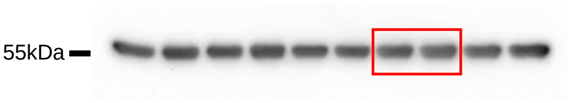

Full unedited image for Supplemental Figure 6_Gpi

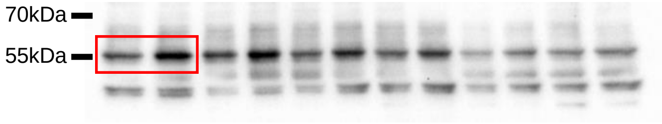

Full unedited image for Supplemental Figure 6_Aldoa

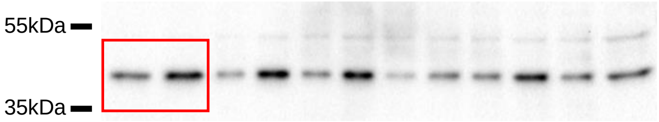

Full unedited image for Supplemental Figure 6_Ldha

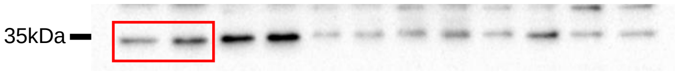

Full unedited image for Supplemental Figure 6_α-tubulin

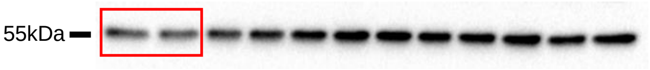

Full unedited image for Supplemental Figure 6_Gapdh

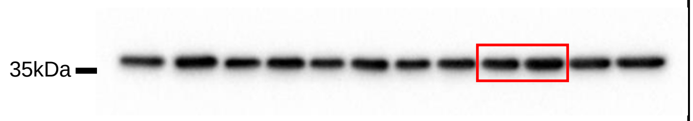

Full unedited image for Supplemental Figure 6_Pgk1

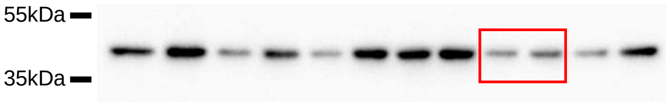

Full unedited image for Supplemental Figure 6_Pgam1

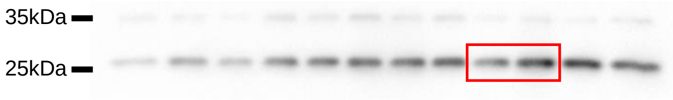

Full unedited image for Supplemental Figure 6_Pdh

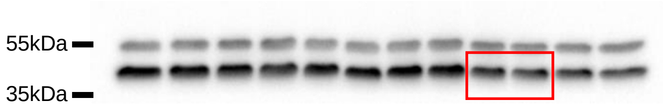

Full unedited image for Supplemental Figure 6_α-tubulin

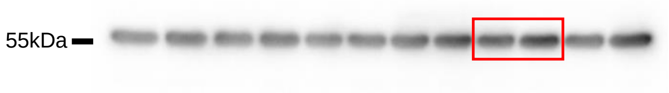

Full unedited image for Supplemental Figure 6_Pkm1

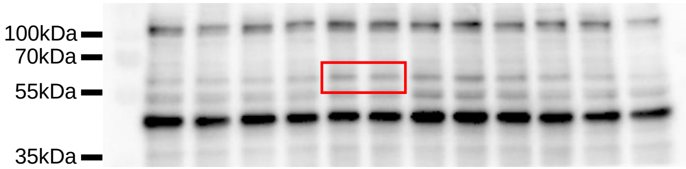

Full unedited image for Supplemental Figure 6_α-tubulin

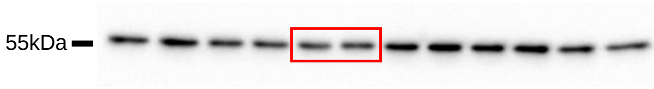

Full unedited image for Supplemental Figure 6_Pkm2

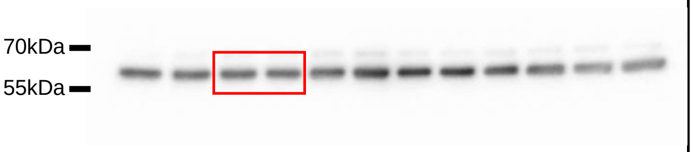

Full unedited image for Supplemental Figure 6_Pck2

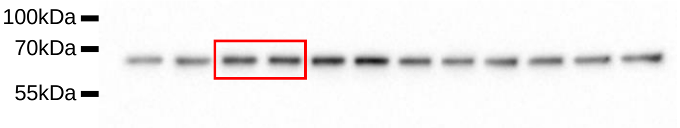

Full unedited image for Supplemental Figure 6_α-tubulin

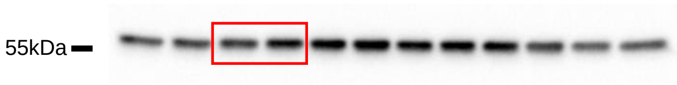

Full unedited image for Supplemental Figure 6_Pc

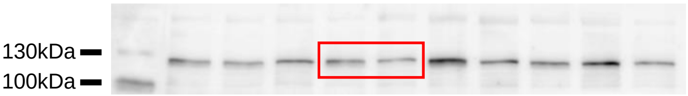

Full unedited image for Supplemental Figure 6_Me1

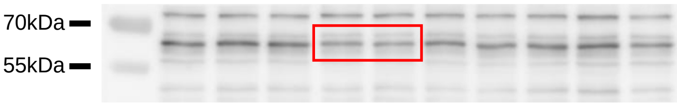

Full unedited image for Supplemental Figure 6_α-tubulin

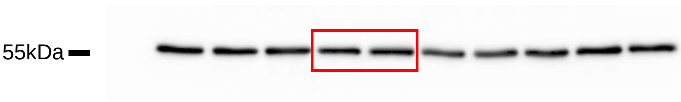

Full unedited image for Supplemental Figure 6_Pdk4

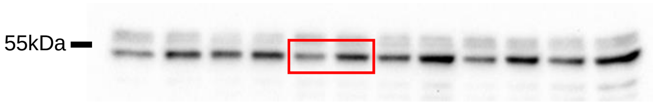

Full unedited image for Supplemental Figure 6_Gfat

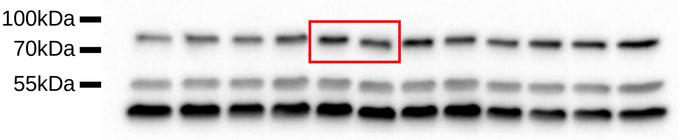

Full unedited image for Supplemental Figure 6_Gpd

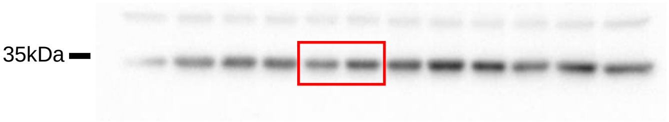

Full unedited image for Supplemental Figure 6_Phgdh

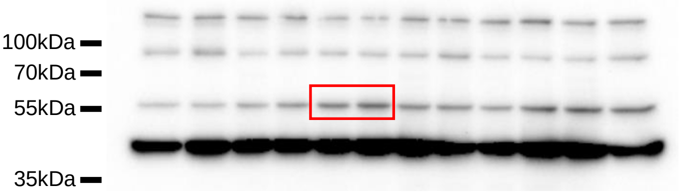

Full unedited image for Supplemental Figure 6_α-tubulin

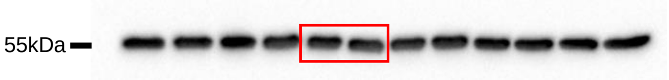

Full unedited image for Supplemental Figure 8A_Yap

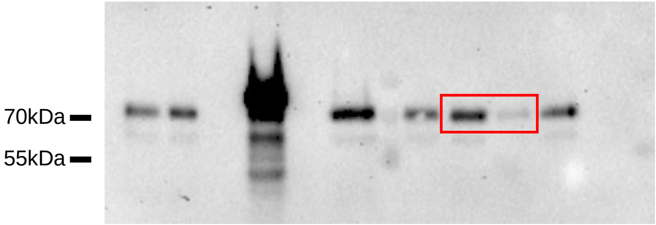

Full unedited image for Supplemental Figure 8A_α-tubulin

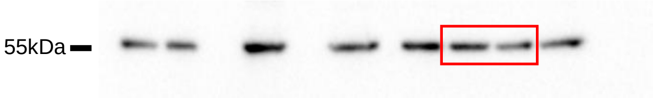

Full unedited image for Supplemental Figure 8D_GLUT1

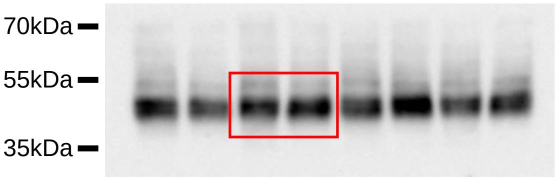

Full unedited image for Supplemental Figure 8D_Hk2

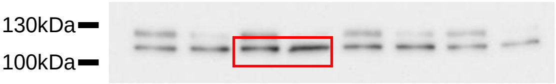

Full unedited image for Supplemental Figure 8D_Pfk1

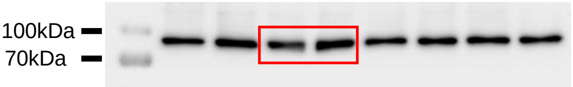

Full unedited image for Supplemental Figure 8D_Aldoa

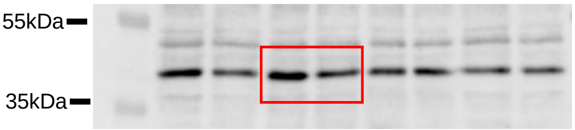

Full unedited image for Supplemental Figure 8D_Gapdh

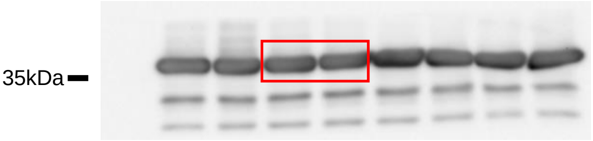

Full unedited image for Supplemental Figure 8D_Pgk1

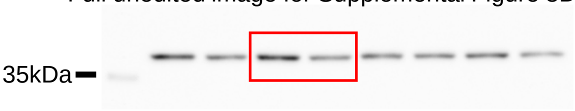

Full unedited image for Supplemental Figure 8D_Pgam1

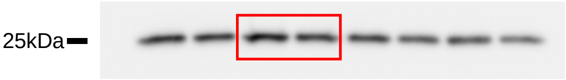

Full unedited image for Supplemental Figure 8D_Eno1

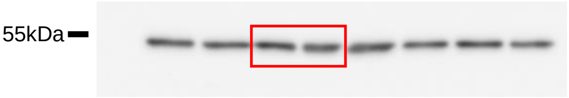

Full unedited image for Supplemental Figure 8D_G6pd

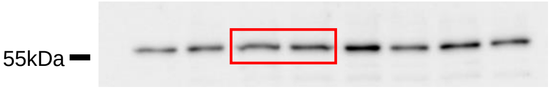

Full unedited image for Supplemental Figure 8D_α-tubulin

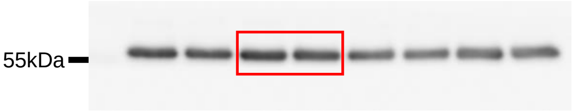

Full unedited image for Supplemental Figure 8D_Pdk4

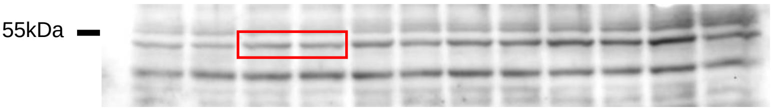

Full unedited image for Supplemental Figure 8D_α-tubulin

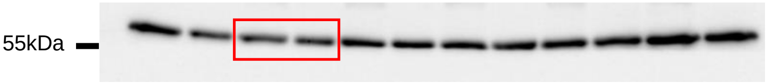

Full unedited image for Supplemental Figure 8D_GLUT4

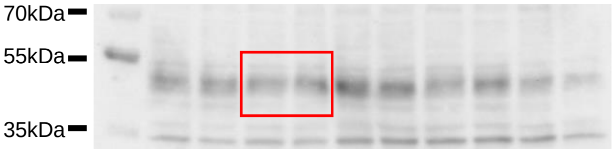

Full unedited image for Supplemental Figure 8D_Hk1

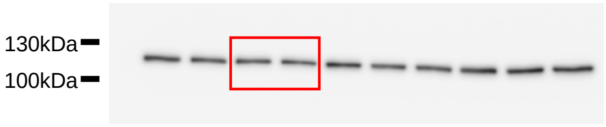

Full unedited image for Supplemental Figure 8D_Gpi

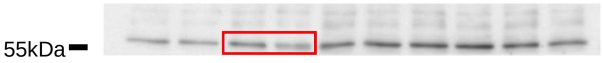

Full unedited image for Supplemental Figure 8D_Pfk2

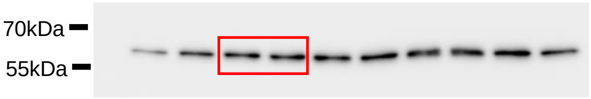

Full unedited image for Supplemental Figure 8D_Pkm1

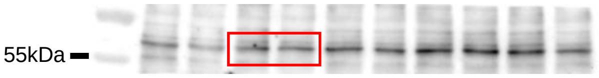

Full unedited image for Supplemental Figure 8D_Pkm2

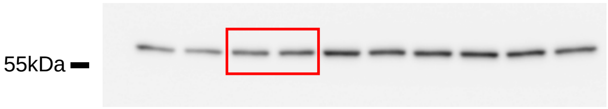

Full unedited image for Supplemental Figure 8D_Ldha

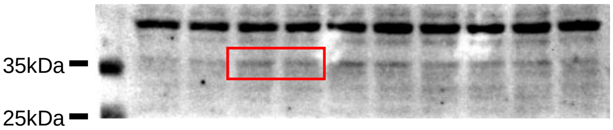

Full unedited image for Supplemental Figure 8D_Pdh

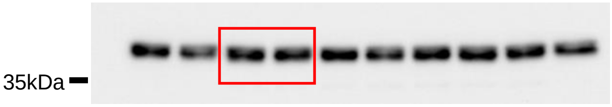

Full unedited image for Supplemental Figure 8D_Pc

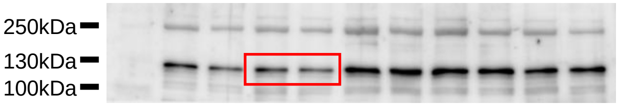

Full unedited image for Supplemental Figure 8D_Me1

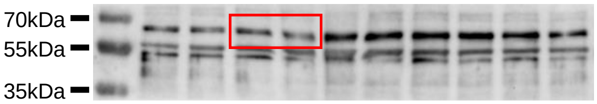

Full unedited image for Supplemental Figure 8D_Pck2

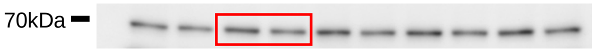

Full unedited image for Supplemental Figure 8D_Gfat

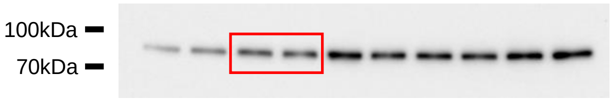

Full unedited image for Supplemental Figure 8D_Gpd

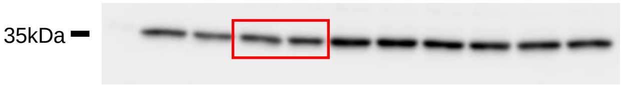

Full unedited image for Supplemental Figure 8D_Phgdh

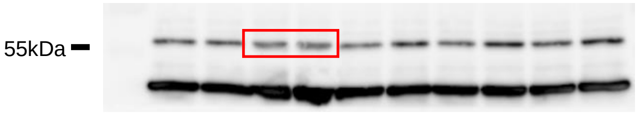

Full unedited image for Supplemental Figure 8D_α-tubulin

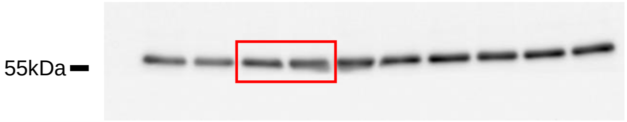

Full unedited image for Supplemental Figure 9C_CI-Ndufb8

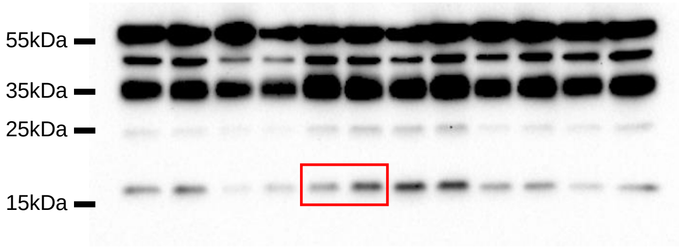

Full unedited image for Supplemental Figure 9C_CII-Sdhb

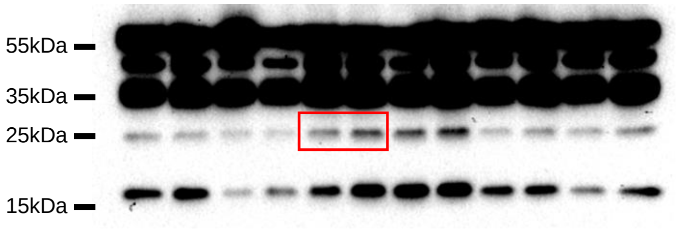

Full unedited image for Supplemental Figure 9C_CIII-Uqcrc

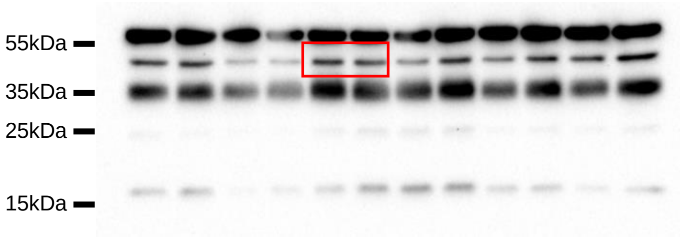

Full unedited image for Supplemental Figure 9C_CIV-Mtco1

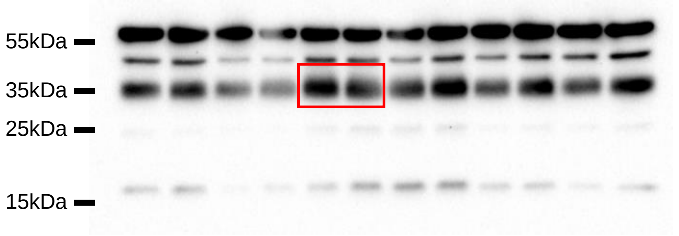

Full unedited image for Supplemental Figure 9C_CV-ATP5a

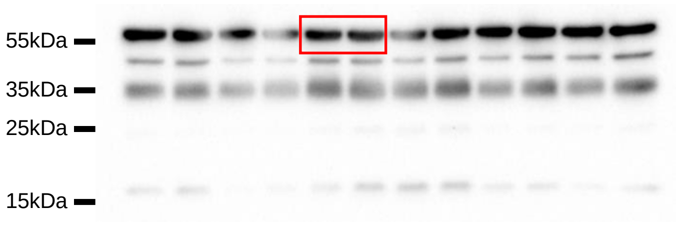

Full unedited image for Supplemental Figure 9C_α-tubulin

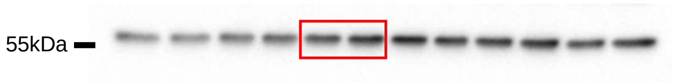

Full unedited image for Supplemental Figure 10A_Glut1

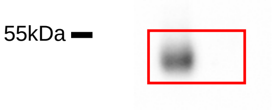

Full unedited image for Supplemental Figure 10A_α-tubulin

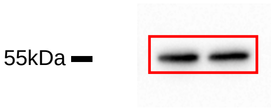

Full unedited image for Supplemental Figure 10B_Hk2

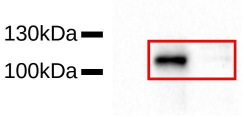

Full unedited image for Supplemental Figure 10B_α-tubulin

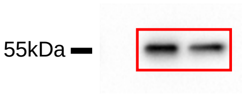

Full unedited image for Supplemental Figure 10C_Gpi

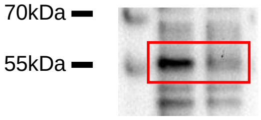

Full unedited image for Supplemental Figure 10C_α-tubulin

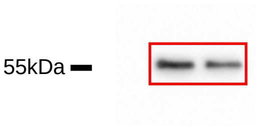

Full unedited image for Supplemental Figure 10D_Pfk1

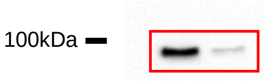

Full unedited image for Supplemental Figure 10D_α-tubulin

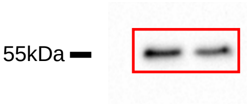

Full unedited image for Supplemental Figure 10E_Aldoa

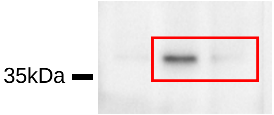

Full unedited image for Supplemental Figure 10E_α-tubulin

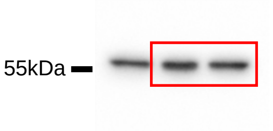

Full unedited image for Supplemental Figure 10F_Gapdh

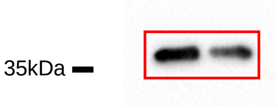

Full unedited image for Supplemental Figure 10F_α-tubulin

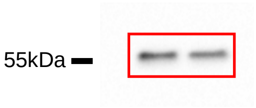

Full unedited image for Supplemental Figure 10G_Pgk1

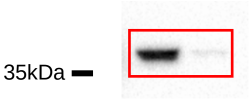

Full unedited image for Supplemental Figure 10G_α-tubulin

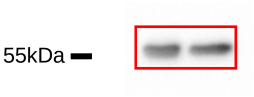

Full unedited image for Supplemental Figure 10H_Pgam1

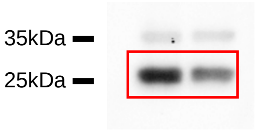

Full unedited image for Supplemental Figure 10H_α-tubulin

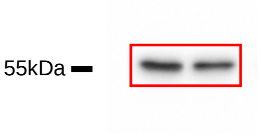

Full unedited image for Supplemental Figure 10I_Eno1

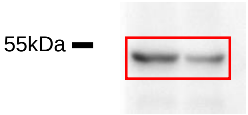

Full unedited image for Supplemental Figure 10I_α-tubulin

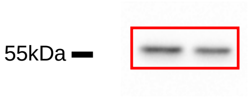

Full unedited image for Supplemental Figure 10J_Pkm1

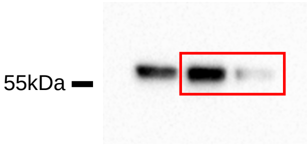

Full unedited image for Supplemental Figure 10J_α-tubulin

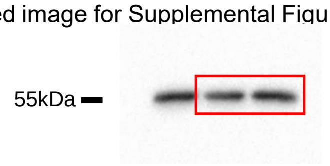

Full unedited image for Supplemental Figure 10K_G6pd

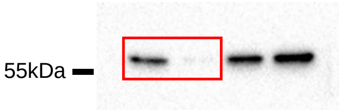

Full unedited image for Supplemental Figure 10K_α-tubulin

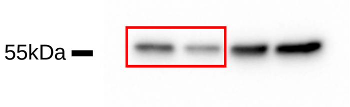

Full unedited image for Supplemental Figure 10L_Gfat

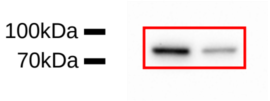

Full unedited image for Supplemental Figure 10L_α-tubulin

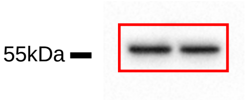

Full unedited image for Supplemental Figure 10M_Gpd1

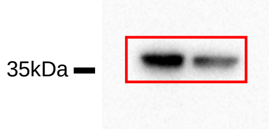

Full unedited image for Supplemental Figure 10M_α-tubulin

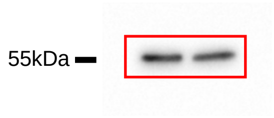

Full unedited image for Supplemental Figure 10N_Phgdh

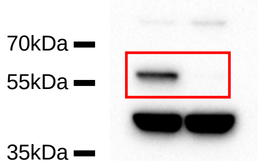

Full unedited image for Supplemental Figure 10N_α-tubulin

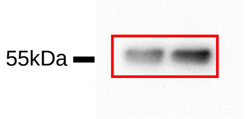

Full unedited image for Supplemental Figure 10O_Pck2

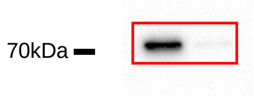

Full unedited image for Supplemental Figure 10O_α-tubulin

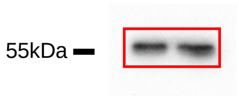

Full unedited image for Supplemental Figure 11A_ YAP

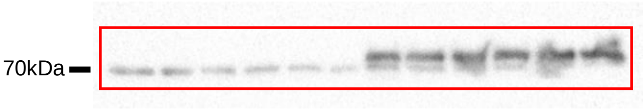

Full unedited image for Supplemental Figure 11A_ H3K4me3

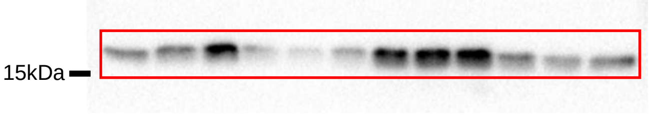

Full unedited image for Supplemental Figure 11A_ H3

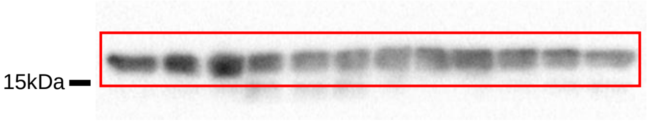

Supplement: Supplemental data [file jci-132-150595-s036.pdf]
